# Optimal control of spin systems

**DOI:** 10.1126/sciadv.aeh1978

**Published:** 2026-09-16

**Authors:** Steffen J. Glaser, Christiane Koch, Niels Chr. Nielsen, Dominique Sugny, Dieter Suter

**Affiliations:** ^1^ Technical University of Munich, School of Natural Sciences, Lichtenbergstrasse 4, 85748 Garching, Germany.; ^2^ Munich Center for Quantum Science and Technology (MCQST), Schellingstrasse 4, 80799 München, Germany.; ^3^ Munich Quantum Valley (MQV), Walther-von-Dyck-Strasse 6, 85748 Garching, Germany.; ^4^ Freie Universität Berlin, Fachbereich Physik and Dahlem Center for Complex Quantum Systems, Arnimallee 14, 14195 Berlin, Germany.; ^5^ Interdisciplinary Nanoscience Center and Department of Chemistry, Aarhus University, Gustav Wieds Vej 14, 8000 Aarhus C, Denmark.; ^6^ Université Bourgogne Europe, CNRS, Laboratoire Interdisciplinaire Carnot de Bourgogne ICB UMR 6303, 21000 Dijon, France.; ^7^ Technische Universität Dortmund, Dortmund, Germany.

## Abstract

Quantum optimal control has become a central tool for designing high-performance pulse sequences in magnetic resonance and quantum technologies. Early developments in NMR relied on heuristic composite and shaped pulses or analytical approaches such as average Hamiltonian theory. The introduction of modern optimal control algorithms enabled the systematic optimization of thousands of control parameters, greatly expanding the achievable performance and scope of pulse engineering. This review summarizes key developments in optimal control of spin and pseudo-spin systems and highlights major applications in magnetic resonance, in quantum technologies, and in the control of atoms and molecules.

## INTRODUCTION

The first 100 years following the discovery of spin have witnessed impressive progress from the initial conception of the idea to careful experimental investigations (*1*, *2*) and new technologies. The ability to manipulate and detect electron spins and nuclear spins has led to today’s widespread use of nuclear spin-based medical magnetic resonance imaging (MRI) (*3*) However, other equally important spin-based magnetic resonance technologies are being used worldwide in chemical, biochemical, and pharmaceutical research, which are far less well known to nonexperts. This includes the fields of nuclear magnetic resonance (NMR) (*4*, *5*) and electron paramagnetic resonance (EPR or ESR) spectroscopy (*6*), which provide physical and chemical information of molecules and materials with atomic resolution. Applications of NMR, MRI, and EPR range from being an indispensable tool in synthetic chemistry to the study of molecular structures and enzyme dynamics in biochemical processes, which are crucial for understanding and diagnosing diseases and for the design of new drugs to cure them, to the development and optimization of solar cells and CO_2_ capturing materials, and industrial process control.

The main developments leading to the currently available sophisticated methods of magnetic resonance for applications in imaging and spectroscopy include the following three main pillars. (i) The first pillar aims to achieve a thorough understanding, measurement, and theoretical modeling of the relevant spin physics, i.e., the spin Hamiltonian representing the types and strengths of interactions among spins and of spins with their environment. (ii) The second pillar concerns the development of magnet technology to efficiently create ultrastrong and extremely stable superconducting electromagnets with fields of up to tens of tesla and the development of electronic devices to precisely modulate the amplitude and phase of NMR–radio-frequency (RF) pulses and EPR-microwave (MW) pulses on the sub-microsecond and the sub-nanosecond timescale, respectively. (iii) The third pillar is formed by the development of powerful methods to reliably control the states and dynamics of spins with extremely high fidelity to ensure the best possible performance of available instruments, taking into account their experimental constraints and imperfections. This review focuses on the third pillar at the theoretical, experimental, and applied levels.

The field of magnetic resonance has a long history of developing methods to achieve better control of spin dynamics, dating back more than eight decades. This was driven by important applications in many different fields and has led to Nobel prizes in physics (Isidor Isaac Rabi, 1944; Felix Bloch and Edward Purcell, 1952), chemistry (Richard Ernst, 1991; Kurt Wüthrich, 2002), and medicine (Paul Lauterbur and Peter Mansfield, 2003) (*7*–*9*).

In every field of science and engineering, controlling the evolution of a system toward a target state is an important and particularly intriguing challenge. As soon as a specific goal is set, we can attempt to reach this goal in an efficient way. Depending on the available resources, this can be a very challenging task. One very notable and well-studied example is the Apollo project: Bringing a team of astronauts to the moon and back was one of the most ambitious scientific and technological projects that had been attempted up to that point. Perhaps more challenging, but certainly less successful examples are the control of social phenomena such as traffic control or economic processes. However, the general approach is similar: Identification of the target and the available resources allows one to search for the combination of parameters that best achieves the objective. Historically, achieving this goal was largely based on trial and error, but in the 1950s, optimal control theory was developed as a scientific foundation based on a generalization of the calculus of variations for controlled systems (*10*). In the 1960s, Pontryagin and his co-workers provided, with the Pontryagin maximum principle (PMP), a rigorous mathematical framework for optimal control theory (*11*–*13*). This pioneering mathematical result led to a series of numerical optimization methods that are used in many different fields, ranging from economics to electronics and space mechanics (*14*–*16*).

Quantum control of spins goes back to the work of Rabi in 1937 (*1*), who solved the driven two-level problem. Further milestones were the development of NMR in the 1940s, the Ramsey experiment (*17*), the spin echo sequence (*18*), the first multiple-pulse sequences in the 1950s (*19*), the development of Average Hamiltonian Theory in the 1960s (*20*) for the computation of the effective Hamiltonian created by a multiple-pulse sequence, and the systematic cancellation of unwanted Hamiltonian terms. Later came the introduction of the product operator formalism (*21*) in the 1980s, greatly facilitating the understanding and spin engineering of advanced NMR pulse sequences. All of these contributed to making NMR so successful (*4*, *5*, *22*) and initiated similar developments in the coherent control of molecular dynamics (*23*–*26*).

**Fig. 1.**
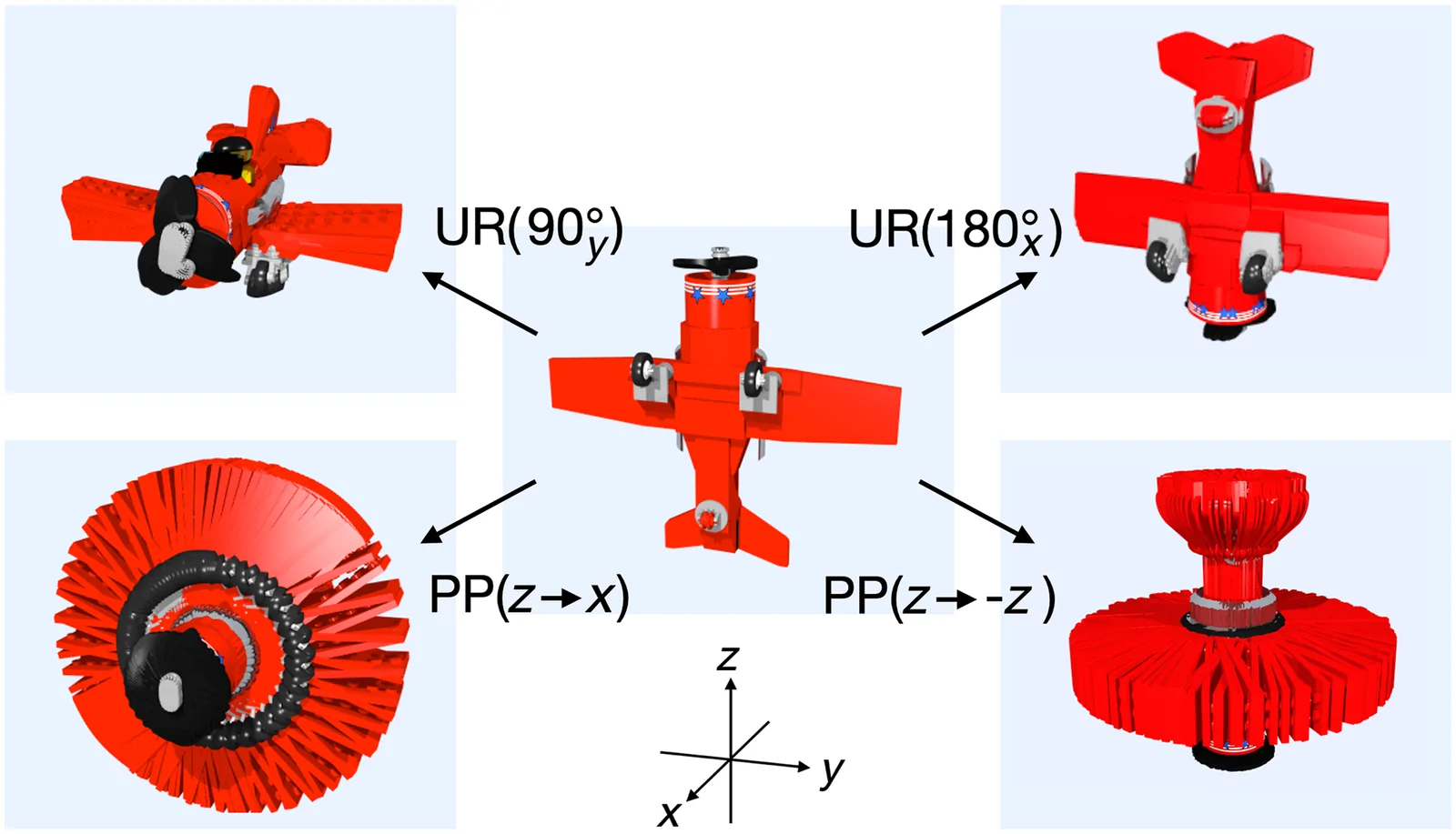
Universal rotation (UR) and point-to-point (PP) pulses. The different properties of UR and PP pulses are illustrated by the effect of numerically optimized broadband 90° and 180° pulses on a large number of toy planes, each corresponding to a different offset frequency, that were initially perfectly aligned.

In the 1970s and 1980s, composite pulses for error-tolerant spin rotations were introduced (*5*, *27*–*29*). Composite pulses were often designed based on symmetry principles to cancel errors (*30*, *31*). Composite pulses can be classified based on their desired effect (*28*, *29*). In addition to other classes, here we highlight two specific pulse types, which play a central role in many applications of optimal control applications:

Pulses that produce a specified rotation for any initial Bloch vector were originally termed “class A” pulses (*28*). Today, they are more commonly referred to as “universal rotation pulses,” a name that more clearly conveys their overall physical effect (*32*). In contrast, composite pulses that are designed to rotate a specific initial point on the Bloch sphere to a specific target point are commonly called point-to-point (PP) pulses (*32*). Originally, they were termed class B2 pulses (*28*). They are often sufficient in practical applications, and they have an additional degree of freedom (see Fig. 1), which, in practical applications, translates into substantially shorter pulse durations (see the “Liquid-state NMR spectroscopy” section).

Numerically optimized composite and shaped pulses for uncoupled spins were introduced in the 1980s (*33*–*35*). Further milestones were the numerical optimization of shaped pulses for coupled spins (*36*, *37*) and numerical optimizations of multiple-pulse sequences for homonuclear and heteronuclear Hartmann-Hahn transfer of polarization in liquids (*38*–*45*) for total correlation spectroscopy (TOCSY) (*40*, *42*, *45*) and tailored correlation spectroscopy (TACSY) (*38*, *41*, *43*, *44*). Other approaches involving adiabatic pulses (*46*, *47*) were also developed during this period. These methods, which are based on specific control mechanisms, can be characterized as intuitive or heuristic. In contrast to modern optimal control approaches, NMR pulse design was largely confined to the optimization of a few discrete parameters: flip angles and phases in composite pulses, or coefficients of simple analytic parametrizations—such as Fourier expansions (*48*), spline functions (*37*), or Gaussian pulse cascades (*49*)—in the case of shaped pulses.

In parallel with this mainly liquid-state NMR-driven development, a large number of advanced solid-state NMR pulse sequences were developed, mainly using average Hamiltonian theory (*20*, *50*, *51*). Among many, these included cross-polarization methods (*52*, *53*), advanced homonuclear decoupling methods (*54*–*58*), a variety of so-called dipolar recoupling methods (*50*, *58*–*62*), and methods like MQMAS (*63*) for quadrupolar nuclei, many of these in combination with magic-angle spinning (MAS) (*64*).

More elaborate pulse engineering methods inspired by optimal control theory were introduced in the mid-1980s (*23*, *65*, *66*), marking the birth of quantum optimal control. The idea was to transpose and adapt the powerful mathematical and numerical tools to quantum dynamics of both spin systems but also atoms, molecules, and more generally chemical reaction dynamics. At that time, the development of quantum optimal control was limited both numerically, in the design of optimal controls for large and complex quantum systems, and experimentally, in the implementation of computed controls. Remarkable progress in pulse-shaping systems and numerical computation methods in the early 2000s marked a turning point for quantum optimal control. The introduction of simple, efficient optimal control algorithms for arbitrary quantum systems composed of uncoupled or coupled spins, such as GRAPE (GRadient Ascent Pulse Engineering) (*67*), led to the rapid deployment of these approaches in subsequent years. In parallel, the stringent requirements of emerging quantum technologies have spurred further advances in optimal control methodology and its application to quantum information carriers across various platforms (*68*–*70*). The efficiency of optimal control algorithms made it possible to increase the number of optimized control parameters from a few tens to tens of thousands or more, i.e., by several orders of magnitude (*71*, *72*). Spin systems were a major application in which quantum optimal control has shown its impact due to the very good agreement between theory and experiment, and the various challenges that needed to be solved (*22*). Notable results range from magnetic resonance spectroscopy (MRS) to imaging in both solid- and liquid-state experiments. Today, the use of quantum optimal control is becoming essential for performing basic operations in quantum technologies. Once again, spin systems in nitrogen-vacancy (NV) centers and other platforms are a key area of application for these techniques.

These elements of the historical development of optimal control demonstrate the fundamental role that spin systems play in this process. This review aims to describe some of the key outcomes of this shared history and also addresses current and future challenges in this area that quantum optimal control can help overcome. The paper is organized as follows. We briefly recall in the “Basics of Spin Control” section the basic tools used to describe spin dynamics. The “Methods” section focuses on the optimal control methods developed in this field. Applications of magnetic resonance, ranging from imaging modalities to liquid- and solid-state NMR spectroscopy and EPR and dynamic nuclear polarization (DNP), are discussed in the “Applications in Magnetic Resonance and Quantum Technologies” section. The “Applications in Atoms and Molecules” section shows how such techniques have been applied to the control of atoms and molecules when they are described as pseudo-spin systems. Concluding remarks and prospective views are given in the “Outlook” section.

## BASICS OF SPIN CONTROL

### Statement of the control problem

Control refers to our ability to manipulate dynamical systems in the desired way. In quantum physics, the control knobs are typically classical electromagnetic fields, and the control targets can be the implementation of a specific dynamics, from given initial to certain final states, but also the minimization or maximization of observables or related quantities such as signal-to-noise ratios. Mathematically, the control problem is formalized in terms of (i) the equation of motion, describing the system dynamics, including the interaction of the system with the external field, and (ii) a suitable figure of merit (FOM) capturing the control target. The latter is often called the cost function or optimization target functional. Optimal control then typically refers to the use of a generalization of the calculus of variation to solve the control problem by treating the control target as a functional of the time-dependent shape of the external electromagnetic field (*68*).

Magnetic resonance, in all its forms, involves manipulating electronic and nuclear spins by interacting with a magnetic field (*5*). This makes it a field in which control techniques can be used, with a multitude of applications extending from the improvement of resolution and sensitivity in spectroscopy to quantum computing. Quantum control is a branch of control theory that aims to perform specific operations on quantum systems using shaped external electromagnetic fields (*68*). This approach has evolved rapidly over the last 20 years to become mature and flexible and can now be implemented on any quantum platform (*68*, *69*). Quantum control is currently an extensive collection of analytical and numerical techniques that can be used to design fast, efficient, and robust protocols while taking experimental limitations and constraints into account. Here, we focus on a specific family of control problems that are well suited to magnetic resonance, in which the system dynamics is governed by a set of first-order differential equations. A brief description of the derivation and the conditions under which these equations of motion can be used is provided below.

### Control of uncoupled spin-1/2 particles

In this section, we review the theoretical principles on which the control of a spin-1/2 particle is based (*4*, *5*, *22*), as described in (*73*). Spin is an intrinsic property of a quantum system. The spin operator, denoted by the vector quantity $\vec{S}=\left(S_{x},S_{y},S_{z}\right)$, exhibits the same properties as angular momentum in quantum mechanics. It satisfies the same commutation relations, i.e., $\left[S_{k},S_{\ell }\right]=i\hbar \varepsilon_{k\ell m}S_{m}$ and $\left[{\vec{S}}^{2},S_{z}\right]=0$, where $\varepsilon_{k\ell m}$ is the Levi-Civita symbol. Because ${\vec{S}}^{2}$ and $S_{z}$ commute, a common basis of eigenvectors, denoted ∣s,m〉, can be introduced such that

$${\vec{S}}^{2}|s,m\rangle =s\left(s+1\right)|s,m\rangle ,S_{z}|s,m\rangle =m|s,m\rangle$$

where −s≤m≤s and *s* is the value of the considered spin. A key physical difference from angular momentum is that *s* is not necessarily an integer, but can also be a half-integer. Below, we consider the simplest case for which s=1/2. The corresponding Hilbert space has a dimension of 2, and the spin operator is given in terms of the Pauli matrices, $\vec{S}=\frac{1}{2}\vec{\sigma }$ with $\vec{\sigma }=\left(\sigma_{x},\sigma_{y},\sigma_{z}\right)$. The spin system has two degenerate energy levels, which can be separated by an external magnetic field via the Zeeman effect. The interaction between the spin and the magnetic field $\vec{B}$ can be expressed as $H=-\gamma \vec{S}\cdot \vec{B}$, where γ is the gyromagnetic ratio. For a static field along the *z* axis, $\vec{B}=B_{0}{\vec{u}}_{z}$, the Hamiltonian reads $H_{0}=-\gamma B_{0}S_{z}$, where $\omega_{0}=-\gamma B_{0}$ is the fundamental (angular) frequency associated with this two-level quantum system.

In magnetic resonance, the observable interest is the magnetic moment denoted $\vec{M}$, which corresponds to the quantum expectation value of the spin operator up to the factor γ, $\vec{M}=\gamma \langle \vec{S}\rangle$. The dynamics of the magnetic moment are governed by the Bloch equation, which can be directly derived from the Schrödinger equation in the case of a pure state (*73*). It can be shown that $\dot{\vec{M}}=\gamma \vec{M}\times \vec{B}$, where $B_{x}\left(t\right)$ and $B_{y}\left(t\right)$ are two time-dependent magnetic fields, each of which is described by two timescales. One timescale corresponds to a fast oscillation at the carrier frequency ω, while the other is associated with the slow evolution of the amplitudes over time. The Bloch equation describes the mechanical torque that the magnetic field exerts on the magnetic moment. This action corresponds to rotations in real space, or unitary operations in Hilbert space. In the absence of dissipation effects, the system evolves on a sphere known as the Bloch sphere. In the presence of only the $B_{0}$ field, the magnetic moment rotates at frequency $\omega_{0}$ around the *z* axis. The Bloch equation can be simplified by considering the corresponding rotating frame. When the carrier frequency ω is chosen to be in quasi-resonance with $\omega_{0}$ and the magnetic field intensity is low enough, the rotating wave approximation enables us to discard all terms involving fast oscillations and retain only those with the slow timescale. In the rotating frame, we lastly obtain the following dynamical equations

$$\left(\begin{array}{c} {\dot{M}}_{x}\\ {\dot{M}}_{y}\\ {\dot{M}}_{z} \end{array}\right)=\left(\begin{array}{c} \Delta \omega M_{y}-\omega_{y}M_{z}\\ -\Delta \omega M_{x}+\omega_{x}M_{z}\\ \omega_{y}M_{x}-\omega_{x}M_{y} \end{array}\right)+\left(\begin{array}{c} -M_{x}/T_{2}\\ -M_{y}/T_{2}\\ \left(M_{0}-M_{z}\right)/T_{1} \end{array}\right)$$ (1)

where $\Delta \omega =\omega_{0}-\omega$ is the resonance offset and $M_{0}$ is the equilibrium state of the magnetization. The first term on the right-hand side represents the effect of the control amplitudes $\omega_{x}\left(t\right)$ and $\omega_{y}\left(t\right)$, which are directly related to the magnetic field amplitudes in the *x* and *y* directions. The second term describes the interaction with the environment in terms of the longitudinal and transverse relaxation rates, $1/T_{1}$ and $1/T_{2}$, respectively. This dynamical evolution can also be subject to nonlinear effects, such as radiation damping, which can be viewed as an additional magnetic field generated by the current induced in the receiver coil (*74*, *75*).

In a standard magnetic resonance experiment, the detected signal is produced by an ensemble of spins rather than by a single spin, resulting in macroscopic magnetization. In the case of a homogeneous ensemble of spin 1/2 particles, the magnetic field can be assumed to be constant throughout the sample. The macroscopic magnetization is therefore also a solution of Eq. 1. However, it is generally not possible to completely eliminate such inhomogeneities, particularly with regard to precise control procedures. These must be taken into account to improve the description of the experimental system. In a first approximation, the inhomogeneities of the static field $B_{0}$ can be taken into account by considering identical spins subjected to a stronger transversal dissipative rate $1/T_{2}^{*}$, with $T_{2}^{*}<T_{2}$. If this approach is insufficient, microscopic modeling is necessary, in which the resonance effect, for example, is a function of the spatial position of the spin in the sample. Note that the same applies to the magnetic field in the (x,y) plane. The control amplitudes, $\omega_{x}\left(t\right)$ and $\omega_{y}\left(t\right)$, are then multiplied by an inhomogeneous factor, depending on the spin. In summary, an accurate derivation involves moving from a three-dimensional (3D) dynamical system given by Eq. 1 to a 3*N*-dimensional dynamic where *N* is the number of spins used to discretize the inhomogeneities of the longitudinal and transverse magnetic fields. Simultaneous control of an ensemble of Bloch equations is performed to achieve robust control in the presence of experimental inhomogeneities. From a theoretical perspective, the total dimension of the system scales linearly with the number of spins, which limits the increase in computational time as *N* becomes larger. It is also worth mentioning that the numerical codes can be easily parallelized, because the different Bloch equations are uncoupled. The importance of robustness in controlling spin systems was quickly recognized in magnetic resonance, where different methods ranging from adiabatic to composite pulses (*27*, *28*) were developed. This issue also led to new results in optimal control theory, from both mathematical (*76*–*79*) and numerical perspectives (*80*, *81*). A classical stability analysis has recently revealed a remarkable connection between the ensemble control achieved by composite pulses and caustics (*82*).

**Fig. 2.**
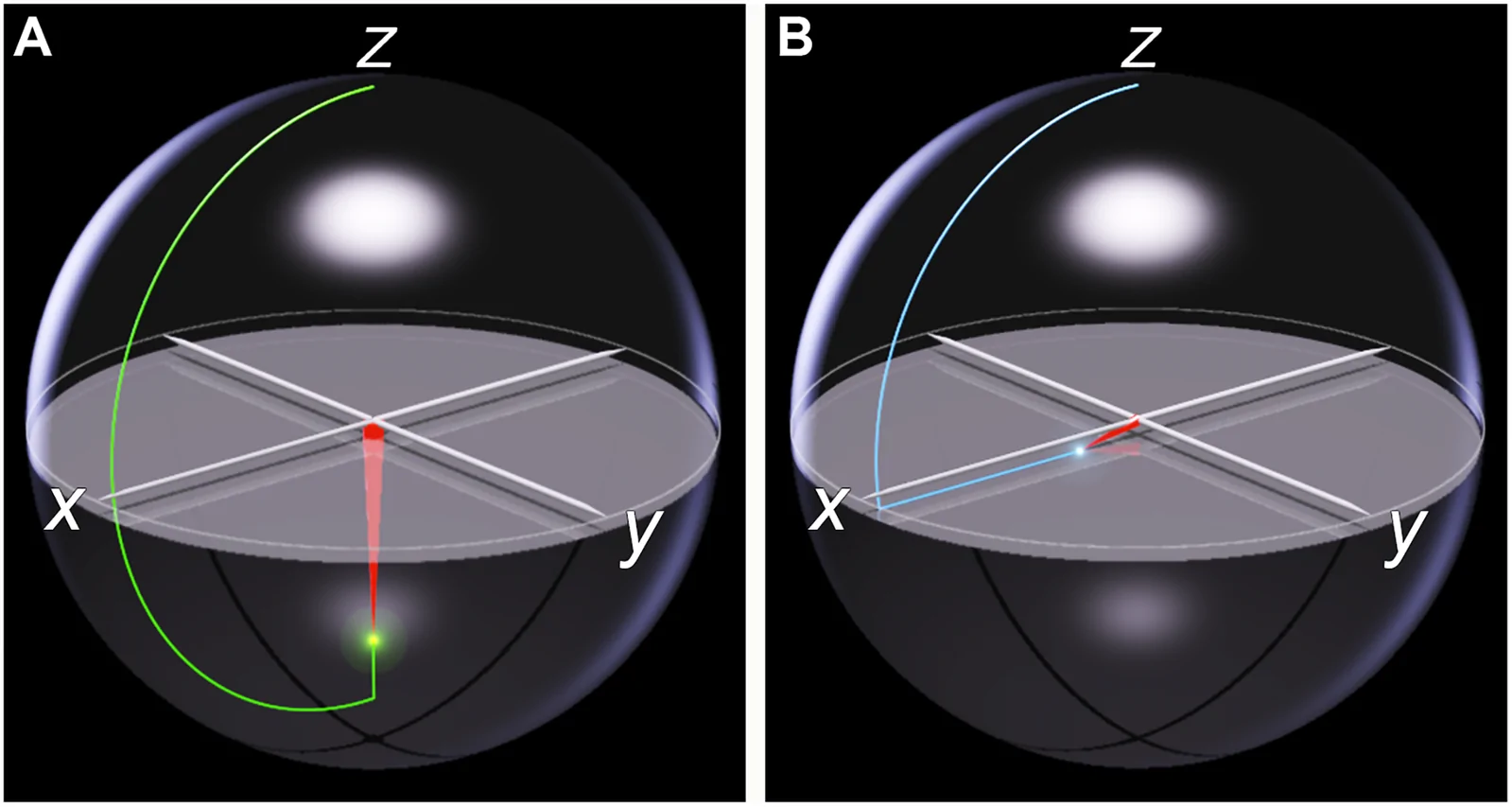
Conventional versus time-optimal saturation pulses based on $T_{1}$ and $T_{2}$ relaxation. Comparison of the trajectories of the Bloch vector to bring the Bloch vector from the *z* axis to the origin of the Bloch sphere using (**A**) a simple inversion recovery–type strategy and (**B**) the time-optimal strategy (*86*) with a trajectory that stays in the magic plane (shown in gray) as long as possible.

For uncoupled spins (without or with relaxation), the magnetization vector (also known as Bloch vector) representation provides a global view of the spin dynamics that is both exact and intuitive, as described below. To acquire an intuitive understanding of the global description of the dynamics, the different terms (offset, pulses, $T_{1}$, and $T_{2}$) in the Bloch equations can be visualized by individual and combined vector fields (*83*). On the basis of the combined vector fields of $T_{1}$ and $T_{2}$, an important, apparently previously unknown geometric structure, the so-called magic plane, was found only 15 years ago, which is fundamental to the control problem to bring the Bloch vector as fast as possible from any initial point to any target point of the Bloch ball. The magic plane is parallel to the equatorial plane, intersecting the *z* axis at

$$z=-\frac{T_{2}}{2\left(T_{1}-T_{2}\right)}$$

(see Fig. 2). For example, when starting from the north pole, the time-optimal strategy to reach the center of the Bloch ball as fast as possible is to flip the Bloch vector to the magic plane, and apply just enough pulse amplitude to keep the vector in that plane—where it shrinks most rapidly under the effect of both $T_{1}$ and $T_{2}$—until the *z* axis is reached. This is followed by a delay during which the Bloch vector is left to relax under the effect of $T_{1}$ to the center of the Bloch ball (*84*–*86*). This previously unknown solution is exact in the ideal case of unbounded pulse amplitudes. An interesting result concerns the evolution of the ratio between this minimum time and the time required to perform a standard inversion recovery protocol. It can be shown that this ratio approaches zero as the ratio $T_{2}/T_{1}$ approaches zero. For $T_{2}\ll T_{1}$ and a sufficiently large control, the optimal control strategy therefore results in an infinite reduction in spin saturation time. In the realistic case of bounded pulse amplitudes, the analysis is mathematically more involved and requires advanced tools of geometric optimal control theory, but the magic plane still plays a central role. For more details, including the intriguing analytical form of the optimal pulse shape, see (*84*, *85*).

This illustrates a feature of geometric optimal control: The solution is described as an optimal trajectory of the spin state, from which the optimal pulse shape is derived. Thus, although experimentally the optimal pulse shape determines the trajectory, conceptually the optimal trajectory defines the pulse shape. More examples of this recurring feature are described in the “Liquid-state NMR spectroscopy” section on relaxation-optimized coherence transfer (*87*–*89*).

**Fig. 3.**
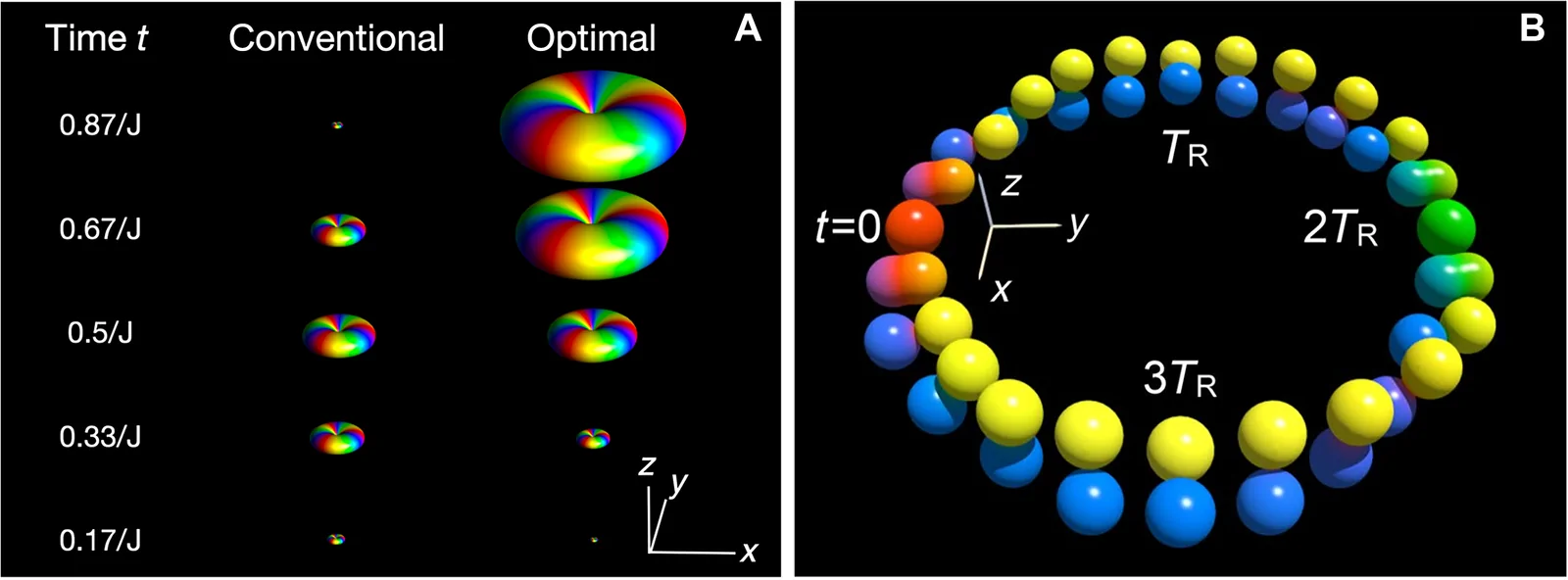
Illustrating the dynamics of density operators and unitary propagators using the DROPS representation. (**A**) The use of the DROPS representation to illustrate the excitation of triple-quantum coherence using conventional and optimal-control pulse sequences [adapted from (*276*) with the permission of AIP Publishing]. (**B**) The DROPS visualization of the propagator evolution during a pulse sequence based on the so-called tennis racket effect [reproduced from (*401*) with permission, Springer Nature]. At t=0, the propagator is the identity operator (represented by a red sphere), which, after two 180° rotations, turns again into the identity operator but with a negative global phase factor (represented by a green sphere).

A canonical and highly useful compact and intuitive visualization of the full density operator of coupled spin systems is the BEADS representation (*90*), which is a special case of general phase space representations of the density operator of uncoupled and coupled spins (*91*–*93*). It was developed based on the DROPS representation (*94*, *95*) and enables visualization and quantification of spin polarizations, single- and multiple-quantum coherences (see Fig. 3A), and (in the context of quantum information applications) even entanglement between spins. DROPS and BEADS representations are not limited to visualizing the density operator but can also be used to visualize arbitrary operators, such as Hamiltonians or propagators (see Fig. 3B). It is interesting to note that the DROPS and BEADS visualizations of propagators also clearly show the global phase of propagators, which plays an important role in the case of broadband UR pulses (see the “Liquid-state NMR spectroscopy” section). In addition to mapping, e.g., simulated density operators or propagators to the corresponding 3D DROPS or BEADS representations, it is also possible to directly measure them using experimental tomography approaches (*96*–*99*). The DROPS and BEADS representations have been implemented in the free SpinDrops (*100*) and QuBeads (*101*) apps for the interactive visualization of the effects of arbitrary pulse/control sequences. In addition, to visualize the full density operator dynamics under an optimal control pulse, it is also possible to map the essential dynamics into simplified graphical trajectories of the magnitude of selected subspaces of states (*102*).

### Control of coupled spin systems

**Fig. 4.**
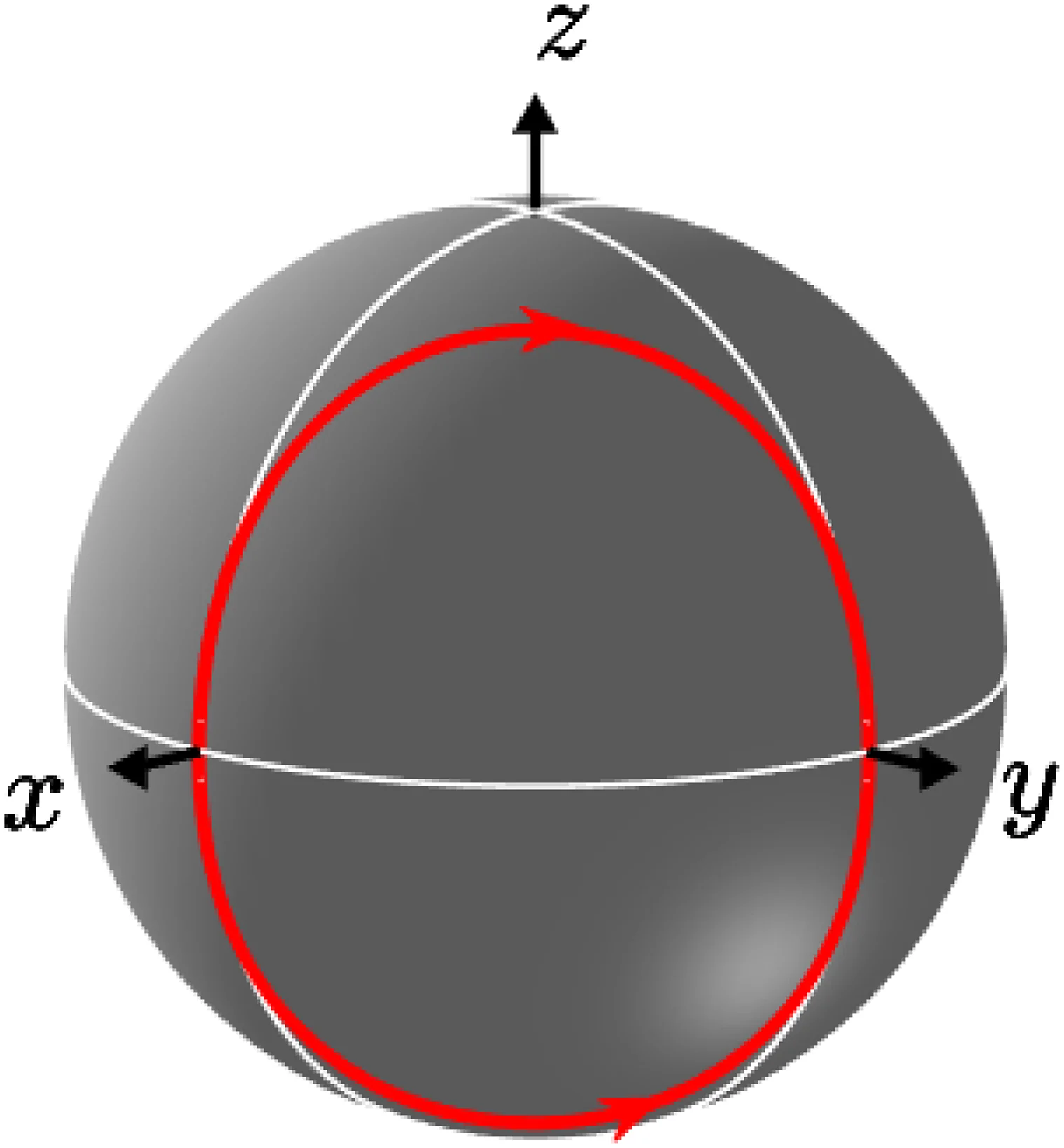
Time-optimal trajectories of the Bloch vector. Plot on the Bloch sphere of the two time-optimal trajectories bringing the system from the *x* to the *y* axis (red solid lines) (*140*, *157*). The white solid lines represent the three great circles.

In both MRS of molecules and quantum information processing, spins are generally not isolated but coupled to other spins. As a result, the dimension of the control problem grows exponentially with the number of coupled spins. The state of coupled spin systems is typically described by the density operator formalism (*4*, *5*, *22*, *103*). Hence, compared to uncoupled spins, coupled spin systems exhibit substantially richer and more complex dynamics and control behavior as illustrated in the “Applications in Magnetic Resonance and Quantum Technologies” section. In particular, it is no longer possible to display the full evolution of the density operator in terms of the trajectory of magnetization vectors (also known as Bloch vectors), which was very useful in the design of the first composite pulses and which provided a global view of the spin dynamics in the case of uncoupled spins (see also Fig. 4).

The pulse shape itself can be visualized in a number of ways, but an especially useful family of visualizations for the analysis of complex optimal control pulses are joint time-frequency representations, particularly their phase-sensitive variants (*104*).

## METHODS

Since the pioneering work of Pontryagin and his co-workers in the 1960s, rigorous and powerful tools have been developed in the field of optimal control theory. In the past decades, these procedures have been adapted to spin and, more generally, quantum systems. They now form a comprehensive and flexible toolbox that can be applied to a wide range of theoretical and experimental situations (*68*, *69*). While optimal control theory typically refers to the set of tools for solving the control problem by determining the shapes of the external fields, the question of whether a solution to the control problem exists is answered by controllability analysis (*105*). We start our methods review in the “Controllability” section with a brief introduction to the Lie algebraic concepts used to this end. In the context of state-to-state transfer by unitary operations, it is relevant to mention unitary bounds on spin dynamics (*106*–*111*). On one hand, these bounds provided clear information on the maximum achievable transfer, and on the other, they were a strong motivator for later use of optimal control in NMR as a means to reach the maximum transfer by pulse sequences under conditions of unitary operation.

Control synthesis, the actual core of optimal control theory, can be carried out with a large range of methods. Before selecting a suitable method, an even more fundamental question concerns the choice of cost function or optimization target functional. Popular choices are the state transfer from an initial state to a desired final state or the implementation of a desired unitary operation. In these cases, the cost function is simply obtained by matching (i.e., projecting) the state or evolution obtained from the time evolution under the control onto the desired target state or operator (*68*, *112*). However, it is important to realize that modern optimal control applications increasingly benefit from the development of more specialized cost functions that are closely adapted to very concrete, actual experimental requirements, which hugely improve the performance of experiments and which vastly broaden the range of applications (see the “Applications in Magnetic Resonance and Quantum Technologies” section).

For NMR and MRI applications, more advanced, experimentally relevant cost functions were designed to minimize phase errors created by short, broadband excitation pulses (*113*), to create a desired (for example linear) phase profile of excited spins as a function of offset frequency (*114*–*116*), and to enforce matched (but arbitrary) offset-dependent Euler angles of pulses in single-scan cooperative pulse sequences (*117*–*119*). Further special cost functions were designed to track the evolution of experimentally relevant parts of the density operator during a control sequence. This makes it possible to design broadband optimal-control decoupling sequences to achieve fully decoupled signals (*72*, *120*) or partially decoupled signals with a residual splitting that has a desired dependence on the offset frequency of the coupled spin (*121*, *122*). The tracking approach was further generalized for robust dynamical decoupling sequences by tracking the evolution of the propagators instead of the evolution of the density operator (*123*). Another special cost function has been developed to improve the signal-to-noise ratio per unit time (*124*–*126*). Even more advanced cost functions have been created to optimize entire pulse sequences for relaxation-dispersion experiments (*127*), to design novel pulse sequences for chemical exchange saturation transfer (CEST) (*128*), and for the optimization of so-called fingerprinting experiments in MRI (*129*). Another specialized cost function for MRI applications is used to optimize imaging contrast based on different relaxation parameters of different tissues (*130*). In the context of solid-state NMR and DNP, cost functions have been created to optimize pulses within the framework of symmetry-based dipolar recoupling experiments (*131*), repeated periodic pulse sequences (*132*, *133*), and optimization of density operator–inspired FOM functions (*134*).

In the field of quantum technology and quantum information processing, specialized cost functions have been developed to maximize the distinguishability between two states evolving under the same control (*135*–*137*). Other choices are to maximize or minimize an expectation value, for example, squeezing (*138*) and cost functions for the optimization of shaped laser pulses for the creation of high-fidelity quantum gates for a qubit—formed by two electronic energy levels of a trapped atom—while minimizing the overall effect of the shaped laser pulses on the motional state of the atom (*139*).

Control synthesis methods can be divided into two broad categories based on the dimension of the dynamical system. The first group, known as geometric methods, aims to solve the optimal equations analytically, or at least with very high numerical precision (*140*). The second group comprises numerical optimization algorithms that iteratively design the external control (*68*). Geometric methods have specific advantages, such as providing a geometric description of optimal dynamics and proving global optimality, from which the physical limits of the control procedure can be deduced. However, they are intrinsically limited to low-dimensional systems. By contrast, the key strength of numerical algorithms is their flexibility, which enables them to be adapted to a variety of control problems, or to specific experimental constraints, and uncertainties. The “Geometric optimal control” and “Numerical methods” sections describe recent theoretical results on control synthesis for spin systems.

### Controllability

The controllability of a quantum system can be determined, under the assumption that no relevant constraints (for example, on the control time or the maximum power of the external field) exist, thanks to the bilinearity of the control problem. It is then possible to establish necessary and sufficient conditions for the controllability of closed and finite-dimensional quantum systems (*141*). These conditions are based on the dimension of the dynamical Lie algebra of the system. Separating the Hamiltonian into (field-free) drift and the operators coupling to the external fields, the dynamical Lie algebra is the vector space comprising these operators as well as all of their successive commutators. The Lie algebra thus consists of the generators of the evolution, and any evolution operator can be generated in finite time when the Lie algebra dimension is maximum and the controls are unconstrained (*142*, *143*). Geometrically, the Lie algebra is the tangent space to the group of all unitaries (of a given Hilbert space dimension *N*), and the full rank guarantees that evolution in any direction is possible. Controllability analysis can be extended to coherent evolutions in infinite-dimensional Hilbert spaces and to open quantum systems (*68*, *69*). In the latter case, the controllability of systems with Markovian dynamics is well understood (*144*), whereas rigorous controllability analysis of non-Markovian open quantum systems remains, by and large, uncharted territory. This lack of rigorous results is relevant mostly for applications in which one seeks to actively exploit the environment; whereas for systems with near-unitary time evolution as in many quantum technology applications, it is sufficient to analyze controllability for the coherent dynamics.

Owing to the finite size of their Hilbert spaces, spin systems have been used as a benchmark problem in controllability. The fact that the Hamiltonian terms are expressed as a product of Pauli operators makes them well-suited to this kind of investigation. For example, for networks of spin-1/2 particles, a relationship between network structure, available controls, and controllability could be shown (*145*–*148*). Another relevant mathematical question concerns the controllability of an ensemble control of spins (*77*, *78*), for which solutions have been given in specific situations (*149*, *150*). This problem is mathematically complex due to the infinite dimension of the controlled system. Moving beyond spin-1/2, controllability can be proven analytically by generalizing the notion of Pauli operators and exploiting the properties of quantum angular momentum in terms of so-called Vandermonde matrices that enable inductive proofs (*151*). This way, one can show that arbitrary rotational subsystems of both linear and asymmetric top rotors, resonantly driven by electric fields, are evolution-operator controllable, provided sufficiently many combinations of (resonant) frequencies and polarization directions are used (*152*, *153*). Here, rotations in physical space become isomorphic to spin thanks to the (controlled) truncation of the a priori infinite-dimensional Hilbert space of quantum rotors. In summary, the controllability analysis allows for identifying the control resources (types of external fields) needed to control spin systems. Once this is done, optimal control can be used to derive the shapes of these fields.

### Geometric optimal control

In geometric optimal control, an optimal control problem is solved using the geometric properties of the dynamical system or advanced analytical tools to derive solutions to optimal equations (*15*, *140*, *154*–*156*). The geometric structure of spin systems makes them well suited for this type of study.

A first general approach is based on the PMP, which converts the optimal control problem into a generalized Hamiltonian system that depends on a control parameter and an adjoint state. The latter plays the role of a Lagrange multiplier to satisfy dynamical constraints. In this framework, solving a control problem involves finding a specific Hamiltonian trajectory that satisfies given boundary conditions and a maximization condition to derive the optimal control. As an illustrative example of this general approach, we present the geometric and analytical solutions to an optimal control problem for a spin-1/2 particle below [see (*140*, *157*) for a rigorous mathematical derivation]. Here, only a heuristic description is given. A similar approach is also proposed in (*158*). The spin dynamic is governed by the Bloch equation $\frac{d}{\mathrm{dt}}\vec{M}=\vec{M}\times \vec{\omega }$, where $\vec{M}=\left(x,y,z\right)$ is the normalized Bloch vector with $x^{2}+y^{2}+z^{2}=1$ and $\vec{\omega }=\left(\omega_{x},\omega_{y},0\right)$ corresponds to the two control parameters along the *x* and *y* directions. The goal of the control is to transfer the system from the state (1,0,0) to the state (0,1,0) in minimum time denoted $t_{f}$ with the constraints $\omega_{x}^{2}+\omega_{y}^{2}=1$. The Pontryagin Hamiltonian $H_{P}$ can be expressed as

$$H_{P}=\vec{p}\cdot \frac{d\vec{M}}{\mathrm{dt}}=\vec{p}\cdot \left(\vec{M}\times \vec{\omega }\right)=\vec{\omega }\cdot \left(\vec{p}\times \vec{M}\right)$$

where $\vec{p}$ is the adjoint state that also satisfies the Bloch equation, $\frac{d\vec{p}}{\mathrm{dt}}=\vec{p}\times \vec{\omega }$, and with the additional normalization $H_{P}=1$. Introducing the angular-like vector $\vec{L}=\vec{p}\times \vec{M}$, it is straightforward to show that $\frac{d\vec{L}}{\mathrm{dt}}=\vec{L}\times \vec{\omega }$. The optimal control maximizes at any time *t* the Hamiltonian $H_{P}$. They are given by $\omega_{x}=L_{x}$ and $\omega_{y}=L_{y}$ with the relation $L_{x}^{2}+L_{y}^{2}=1$. Using the Bloch equation satisfied by $\vec{L}$, we then obtain that $L_{z}=\ell$ is a constant and that the optimal controls are given by

$$\omega_{x}=L_{x}=\pm \text{sin}\left(\ell t\right),\omega_{y}=L_{y}=\mp \text{cos}\left(\ell t\right)$$

where we use the relation $L_{x}\left(0\right)=0$. Because $L_{y}\left(t_{f}\right)=0$, we also have $t_{f}=\frac{\pi }{2\ell }$. The Bloch equation for $\vec{M}$ can be integrated based on the control expressions. We have $z\left(t\right)=\pm \frac{\text{sin}\left(\sqrt{1+\ell^{2}}t\right)}{\sqrt{1+\ell^{2}}}$. With condition $z\left(t_{f}\right)=0$, we arrive at $\ell =\frac{1}{\sqrt{3}}$ and a minimum time equal to $t_{f}=\frac{\sqrt{3}}{2}\pi$. The corresponding optimal trajectories are represented on the Bloch sphere in Fig. 4. It is worth comparing this time-optimal solution with a naive approach of first applying a $90{{}^{\circ}}_{y}$ pulse followed by a $90{{}^{\circ}}_{x}$ pulse, to bring the Bloch vector from the *x* axis to the *z* axis and then from the *z* axis to the *y* axis. The total time is twice the duration of a 90° pulse. Here, this is a time of π because the maximum pulse amplitude is equal to 1. We deduce that the minimum time is only $\frac{\sqrt{3}}{2}≃0.87$ times the duration of the naive approach. This approach has been used in a variety of spin systems for state-to-state transfer in both closed (*140*, *159*–*161*) and open dynamics (*84*, *86*, *88*, *162*, *163*), but also for SU(2) operations (*164*, *165*) or in coupled spins (*166*, *167*). Recent studies have demonstrated the applicability of this approach to higher-dimensional situations. For example, it can be used to design optimal and robust solutions (*76*, *79*, *168*–*173*) or optimal and selective (*174*, *175*) pulses.

Another geometric method closely related to controllability analysis involves using the properties of the system’s dynamical Lie algebra. The Cartan decomposition enables the evolution operator to be expressed as a product of factors, each of which depends on an element of the Lie algebra. When the controls are unbounded, this approach provides a lower bound of the minimum time required to reach the target state. This procedure has been successfully applied to coupled spin chains (*166*, *176*–*179*). Additional details about the application of geometric optimal control to spin systems are given in the “Liquid-state NMR spectroscopy” section.

### Numerical methods

A variety of numerical optimization methods have been developed for designing control protocols in spin and quantum systems. Such procedures had been known in this field since the 1980s (*65*, *66*, *180*), but a renewed interest emerged at the beginning of the 2000s with the advent of numerical facilities and the development of electronic devices that can shape electromagnetic pulses over spin dynamics timescales. A breakthrough idea was the introduction of GRAPE in 2005 (*67*), a gradient-based algorithm dedicated to spin dynamics that uses piecewise constant controls. GRAPE is an extension of an optimization algorithm (*65*) that was originally developed for uncoupled spin systems and has been adapted to coupled spins. GRAPE and its various formulations such as its second-order generalization (*181*), referred to below as GRAPE, are currently among the most widely used optimization algorithms in both the spin and quantum communities. Other efficient methods, such as Krotov’s approach (*182*–*186*), CRAB (*187*), and GOAT (*188*), were then developed to improve the optimization procedures in specific cases. The key distinction is whether the algorithm uses gradient (and possibly also higher order derivative) information (*67*, *181*–*186*, *188*) or whether it merely evaluates the target functional (*187*). Readers interested in the subject can find a detailed classification of the algorithms in recent review papers (*68*, *69*). A mathematical formulation of quantum numerical algorithms can be found in (*189*).

We first present a simple argument, as described in (*158*), to explain how such algorithms work. Gradient-based algorithms such as GRAPE or Krotov’s method can be directly derived from the PMP. We illustrate this by considering the example given in the “Geometric optimal control” section. Fixing the control time $t_{f}$, we define the “fidelity” as $F=\vec{M}\left(t_{f}\right)\cdot {\vec{M}}_{f}$ to measure the distance of $\vec{M}\left(t_{f}\right)$ to the target state ${\vec{M}}_{f}=\left(0,1,0\right)$ (*F* is not a proper distance measure but sufficient for the purpose of optimization). For simplicity, we assume that there is no constraint on the control. The maximization condition of $H_{P}$ then becomes $\frac{\partial H_{P}}{\partial \vec{\omega }}=0=\vec{p}\times \vec{M}$ such that the optimality equations can be expressed as

$$\frac{d\vec{M}}{\mathrm{dt}}=\vec{M}\times \vec{\omega };\frac{d\vec{p}}{\mathrm{dt}}=\vec{p}\times \vec{\omega };\vec{p}\times \vec{M}=0$$

with boundary conditions $\vec{M}\left(0\right)={\vec{M}}_{0}=\left(1,0,0\right)$ and $\vec{p}\left(t_{f}\right)=\frac{\partial F}{\partial \vec{M}\left(t_{f}\right)}={\vec{M}}_{f}$. This system of nonlinear coupled differential equations with both initial and final conditions can be solved using an iterative algorithm based on the gradient of the fidelity *F* with respect to the control. The gradient can be written directly in terms of the Pontryagin Hamiltonian $H_{P}$ as $\frac{\partial H_{P}}{\partial \vec{\omega }}=\vec{p}\times \vec{M}$.

A standard first-order iterative algorithm can be described as follows [see (*158*) for details]. First, we initialize the iteration using a guess control ${\vec{\omega }}_{0}\left(t\right)$. In step *k* of the algorithm with a control ${\vec{\omega }}_{k}$, we propagate the state $\vec{M}$ from ${\vec{M}}_{0}$ forward and the adjoint state $\vec{p}$ from ${\vec{M}}_{f}$ backward. The control ${\vec{\omega }}_{k+1}$ is obtained from ${\vec{\omega }}_{k}$ as

$${\vec{\omega }}_{k+1}={\vec{\omega }}_{k}+\varepsilon \left(\vec{p}\times \vec{M}\right)$$

where ε is a sufficiently small positive parameter. In such conditions, the algorithm converges toward a local optimal solution of the problem. Several ready-to-use implementations of such iterative algorithms exist, and we provide a brief description of the software available that can be used to derive pulse shapes for the control of spin systems.

A comprehensive numerical software package for magnetic resonance is Spinach, an open-source Matlab package (*190*), with many different functionalities that can be used in MRI, EPR, or NMR. Spinach includes optimal control algorithms. The widely used open-source software package SIMPSON (*191*) initially developed for solid-state NMR simulations offers a comprehensive set of optimal control procedures (*192*), also lately including operator-splitting applicability and optimal control for EPR and DNP (*193*). Also, focusing on pulsed DNP, optimal control numerical methods exploiting control of effective Hamiltonians by optimizing periodic sequences have been presented (*132*). Open quantum systems with Markovian dynamics can be simulated using the QuTiP Python package (*194*), which is widely used in the quantum technologies community. QuTiP natively includes basic implementations of GRAPE and CRAB, whereas the krotov Python package is built on top of QuTip (*186*). For Krotov’s method, an implementation in Julia is also available, which includes automatic differentiation. Another powerful software for open- and closed-loop optimizations featuring several algorithms is the Python library QuOCS (*195*), which has been successfully applied to NV centers. While originally not developed for optimal control, the SpinDrops software (*100*) allows for implementing control pulse sequences and generating optimal protocols using GRAPE while also providing a pedagogical tool to visualize spin dynamics.

## APPLICATIONS IN MAGNETIC RESONANCE AND QUANTUM TECHNOLOGIES

Before looking at specific applications in MRS in later sections, in the “Magnetic resonance imaging” section, we focus on applications of optimal control methods in MRI. In the vast majority of MRI applications, the spins of the hydrogen nuclei in water are excited and detected. Because of the symmetry of the H_2_O molecule, couplings between nuclear spins can be neglected, resulting in an effective two-level system that can be conveniently described using the Bloch equation (see the “Basics of Spin Control” section). However, even in this case, it turns out to be highly advantageous to actually use the Liouville–von Neumann equation for the optimal control–based design of refocusing pulses, to exploit the fact that (in contrast to standard rotation operators) the unitary time-evolution operator carries global phase information, which is crucial for the efficient optimization of broadband or band-selective or refocusing pulses (see the “Magnetic resonance imaging” and “Liquid-state NMR spectroscopy” sections for more details). In the “Liquid-state NMR spectroscopy” section, we consider the case of liquid-state NMR, where typically more complex molecules with correspondingly more complex spin systems are investigated. However, compared to solid-state NMR, spin-spin couplings are relatively small and can be neglected in some applications of optimal control–based pulse design. In other optimal control–based pulse design problems, the presence of spin-spin couplings enables the spectroscopist to steer the spin dynamics in specific ways, e.g., by transferring polarization or coherence in a well-defined manner between spins or by exciting specific orders of multiple-quantum coherence. In several nontrivial use cases of liquid-state NMR, the corresponding Hamiltonian is rich, but still simple enough to not only allow for numerical optimal control–based pulse optimizations but also apply approaches of geometric control theory to even provide a full analytical understanding of the optimal pulses. In the “Solid-state NMR spectroscopy” section, we look at optimal-control applications of solid-state NMR in which typically a powdered sample is studied. This is quite challenging due to relatively large spin-spin couplings, the need to do powder averaging over many crystallite orientations, and a defined temporal modulation of the spin-spin coupling and resonance frequencies by mechanically rotating the solid sample at high spinning speeds. The “EPR and DNP” section addresses recent use of optimal control in EPR and DNP, where the control mainly addresses MW manipulation of electron spins. Electron spin interactions are typically orders of magnitude larger than nuclear spin interactions, with the consequences of very large nuclear-electron spin systems and fast relaxation calling for operation with intense MW pulses at the nano- to microsecond timescale. In such cases, it may prove beneficial to optimize effective Hamiltonians rather than state-to-state transfer among density operators in huge spin systems.

In the “Quantum technology approaches” section, recent applications of optimal control to quantum technologies are discussed with a focus on NV centers not only for quantum sensing and computing, but also for a range of experimental platforms based on spins and pseudo-spins. We describe how optimal control procedures can mitigate the effects of experimental uncertainties and noise to enhance the precision of various elementary operations.

### Magnetic resonance imaging

MRI is a key example of a complex system where optimal control may radically affect method development. In its basic version, it is spin system–wise simple, in the sense that it typically may be conducted in a spin-1/2 Bloch sphere or density operator setup, and yet highly challenging as measurements need spatial encoding, is influenced by sample-dependent external and RF field inhomogeneities, and is often limited in terms of measurement time, moving objects, instrumentation, and safety regulations. The application of optimal control in the design of pulse sequences for MRI dates back to the 1980s, with Conolly *et al.* (*65*) designing frequency-selective pulse sequences. Analytical solutions using hyperbolic secant pulses were also derived for selective population inversion (*196*). Optimal control has later been widely used for the design of MRI experiments, including focus on spatial selectivity, contrast enhancement, singlet-state excitation, and combinations with artificial intelligence.

An important aspect of MRI is to have RF pulses that can provide accurate manipulation of spins in the presence of static ($B_{0}$) and RF ($B_{1}$) field inhomogeneities, relaxation, and imperfect gradients while having sufficiently large flip angles to be efficient. The first challenges were initially addressed through optimal control design of RF pulses with arbitrary excitation profiles based on the Bloch equation (*65*). Later, this was extended by optimal control pulses compensating for $T_{1}$ and $T_{2}$ effects in the presence of RF inhomogeneity (*197*), and practical applications on optimal control developed MRI pulse sequences simultaneously providing robustness to $B_{0}$ and $B_{1}$ inhomogeneities while considering timing and relaxation effects (*198*). The important combined consideration of timing, relaxation, and RF inhomogeneity was later, in the context of NMR, addressed in more detail by incorporating specifically relaxation and RF inhomogeneity into the optimal control cost function (*199*, *200*). This has been followed up by numerous studies including, for example, the development of slice-selective broadband refocusing pulses to reduce chemical shift artifacts (*201*, *202*), delayed spin echoes in the limit of weak dephasing (*115*, *203*), optimal CEST pulses (*128*), optimal fingerprinting experiments (*129*), pulses for constant gradient elastography (*204*), the design of optimal pulses for multislice or slice-selective excitation (*205*, *206*), large-flip angle pulses for high-field MRI (*207*), and time-optimal pulses taking into account gradient imperfections (*208*).

**Fig. 5.**
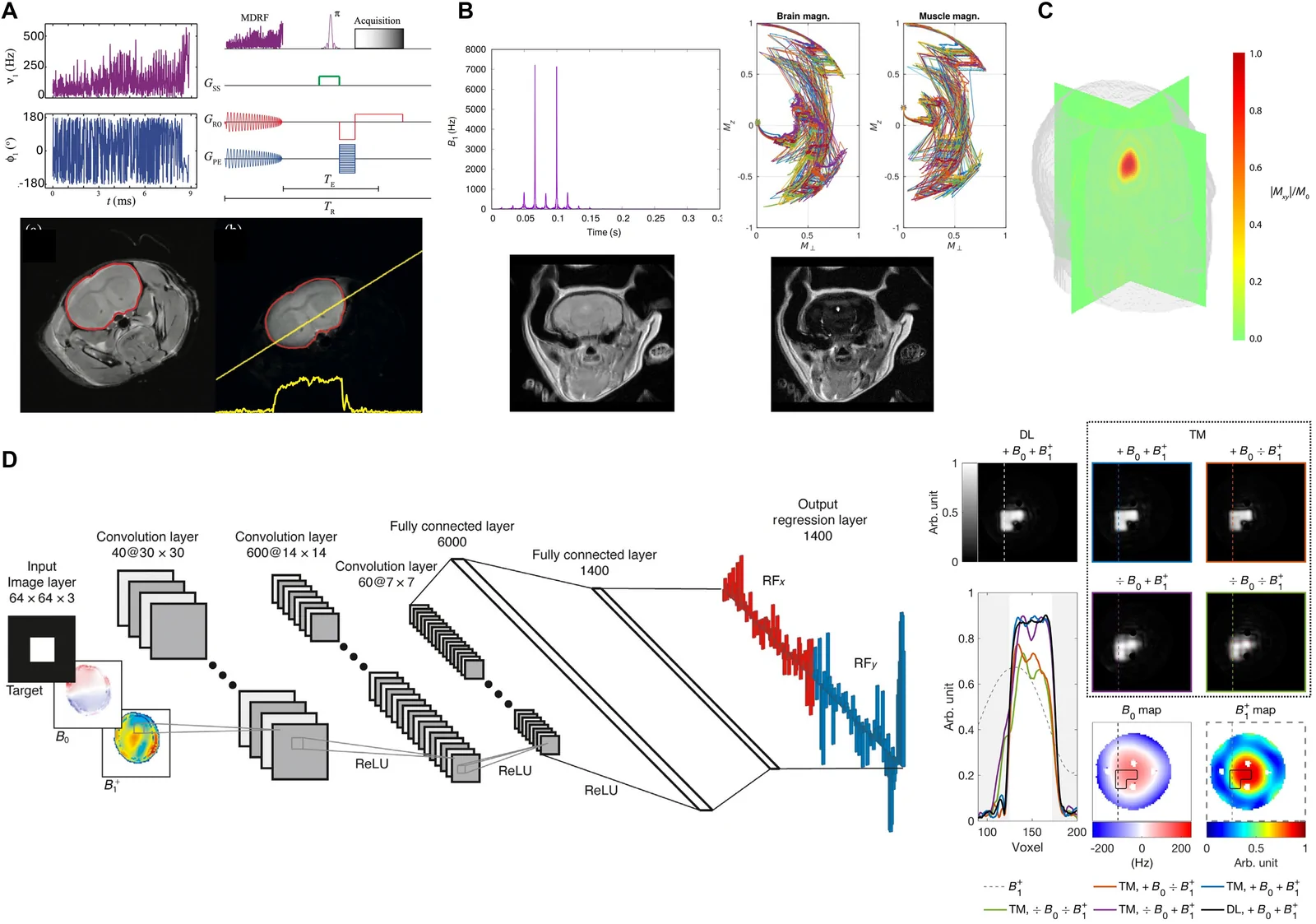
Examples of optimal control design of MRI experiments. (**A**) Spatial selection of part of mouse brain at 17.4 T for improved spatial resolution and ability to quantify species in well-defined volumes (*184*). Adapted from (*184*), Copyright 2012 AIP Publishing, https://doi.org/10.1063/1.4739755. (**B**) Radio-frequency (RF) field, magnetization trajectories, and mouse brain MRI images without (left) and with (right) contrast optimization (*218*). Reproduced from (*218*) with permission from Elsevier. (**C**) Visualization of magnetization components in spatial region excited using optimal control SAR-controlled MRI (*220*). Reproduced from (*220*) with permission from John Wiley and Sons. (**D**) The principle of deep learning– and optimal control–based real-time MRI (*231*). Reproduced from (*231*) with permission from John Wiley and Sons.

Spatial selectivity is another important aspect of MRI aimed at reducing the field of view and thereby improving spatial resolution or selectively exciting specific areas for quantification. Optimal control represents a highly versatile tool in the design of spatially selective MRI methods. In 2009, Grissom *et al.* (*209*) demonstrated the design of spatial selective MRI experiments by least-squares optimization of pulses within the small–tip angle approach. Later, Krotov quasi–Newton-based optimal control procedures were used to design spatial-selective pulses for MRI (*184*) as demonstrated in Fig. 5A by selective excitation of a region of a mouse brain at 16.4 T. This approach was later extended to combinations with DNP (*210*) and real-time spatially selective experiments (*211*). Optimal control has, in the context of spatial section, been used in several contexts, including, for example, the use of multislice multiband parallel RF excitation (*212*), active control of the spatial MRI phase distribution (*116*), and 3D spatially selective refocusing pulses for reduced field-of-view imaging (*213*).

**Fig. 6.**
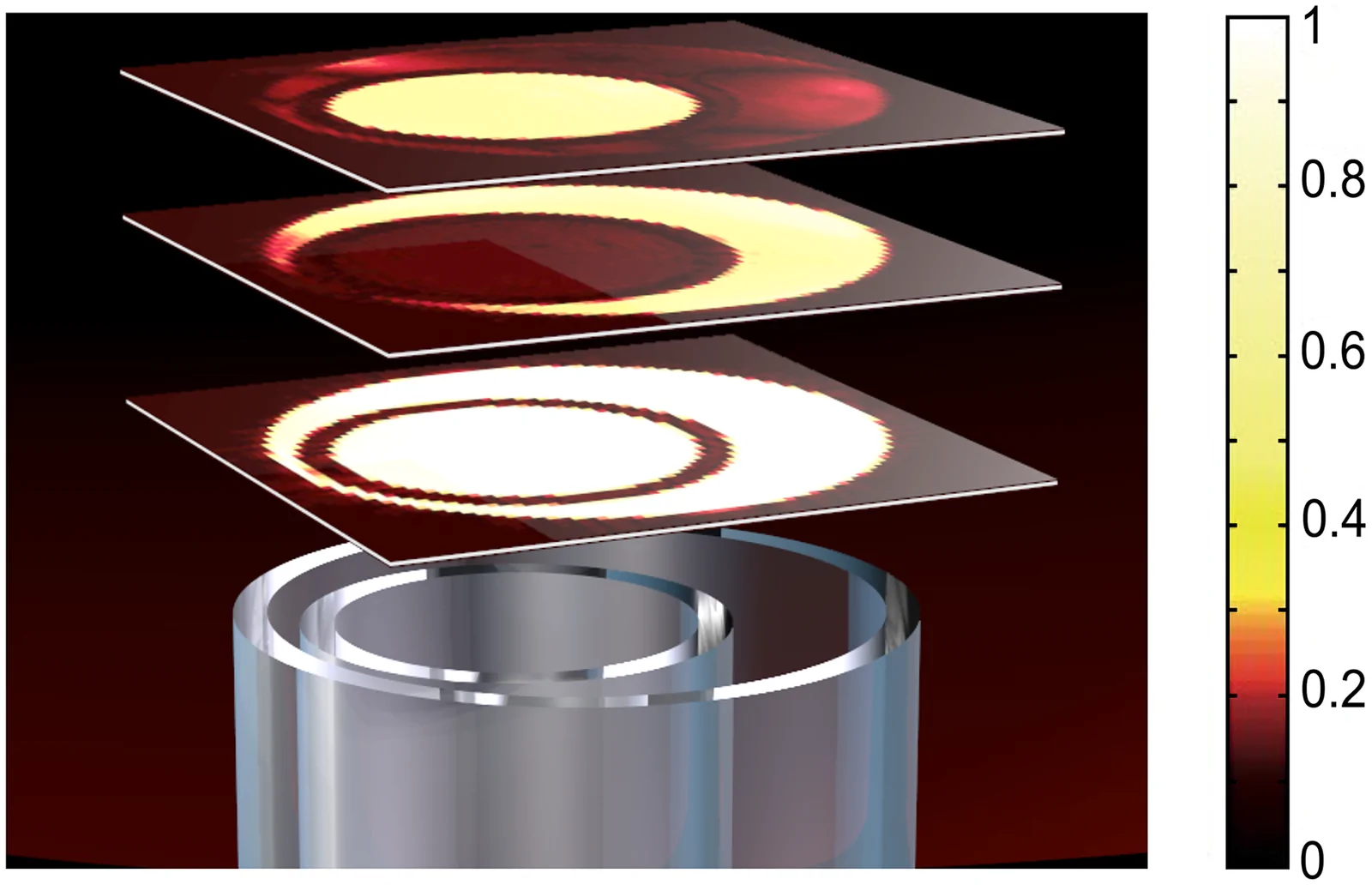
Optimal MRI contrast pulses based on different $T_{1}$ and $T_{2}$ relaxation rates. The figure shows experimental MRI microimaging results of in vitro tests of optimized imaging contrast pulses based on both $T_{1}$ and $T_{2}$ rates (inner volume: $T_{1}=$ 1800 ms, $T_{2}$ = 260 ms; outer volume: $T_{1}$ = 1400 ms, $T_{2}$ = 60 ms). Adapted from (*130*) with permission, Springer Nature. The color scale shows the pixel intensity relative to the maximum intensity of the reference image without contrast (shown at the bottom).

MRI can use optimal control to determine the maximum achievable contrast based on criteria like relaxation time differences or diffusivity (*130*, *214*, *215*), thus finding the corresponding physical limits. The optimal pulse sequence is used as a preparation scheme before image acquisition. To understand the physical limits of imaging contrast, optimal contrast pulses were derived analytically using geometric optimal control for the idealized scenario of negligible $B_{0}$ and $B_{1}$ inhomogeneities (*15*, *130*, *216*, *217*) (see also the “Liquid-state NMR spectroscopy” section). In addition, saturation pulses that are tolerant to realistic ranges of these inhomogeneities were optimized numerically using GRAPE and experimentally demonstrated in vitro (*130*) and in vivo (*218*) as exemplified in Figs. 5B and 6. A suboptimal solution with a limited number of control parameters was proposed in (*219*) to reduce the computation time of the optimal control.

A matter of importance in practical applications of MRI is the specific absorption rate (SAR), which addresses the potential risk of local temperature elevations induced by the highly nonhomogeneous distribution of RF absorption rates. Current patient safety guidelines include strict SAR limitations that have to be enforced for every patient. As such, this is an issue in the design of large flip-angle pulses and spatial or contrast-enhancing pulse sequences for patient-involved MRI. This applies also to optimal control design of MRI sequences, which in turn may represent the best way to cope with it, by incorporating SAR into the cost function (*220*–*222*). An example of a magnetization profile from a SAR-constrained optimal control optimization is shown in Fig. 5C.

Long-lived singlet states have unusually long spin polarization lifetimes (*223*), with great perspectives for MRI and MRS and their combinations with hyperpolarization technology (*224*). The method requires molecules with strongly coupled, near-equivalent spins and methods enabling transfer in and out of the long-lived spin states used to store polarization protected from fast longitudinal relaxation. It has been demonstrated (*225*) that this effect may be used to markedly enhance dissolution DNP (*226*)–enhanced MRI sensitivity. Transfers in and out of the singlet states may favorably be accomplished using optimal control pulse sequences compatible with typical MRI scanner performance (*227*). Singlet-state MRI, with optimal control optimized pulses, may also be used to accurately target spin molecular signals in MRI and MRS (*228*) or to directly excite specific molecules for spectroscopic imaging (*229*, *230*).

Last, we want to address the combination of optimal control and deep learning. Such combinations are highly interesting in the context of learning and self-adapting principles in pulse sequence engineering. When addressing MRI, the gain may be even bigger in the sense that optimal control is not sufficiently fast to real-time develop pulse sequences taking into account the (time varying) influence of the object on $B_{0}$ and $B_{1}$ inhomogeneities. It has been demonstrated that the use of neural networks and deep self-supervised learning protocols may be valuable in the design of MRI pulse sequences (*231*–*235*). Among these, we address here attention to those combining deep learning with optimal control pulse sequence design (*231*, *232*). As illustrated in Fig. 5D, the learning network trains (left) on a huge number of optimal control pulse sequences prepared for different 2D geometries and $B_{0}$ and $B_{1}$ inhomogeneity profiles. In this setup, the trained convolutional network predicts optimal field inhomogeneity–compensated 2D RF pulses (right) with the quality of optimal control experiments in less than 10 ms, which is essential for clinical practice.

### Liquid-state NMR spectroscopy

In the field of liquid-state NMR, many systematic analytical and numerical optimal-control studies have been conducted to establish, for the first time, the physical limits of pulse performance. On the one hand, in many cases, this provided spectacular performance improvements compared to what had been possible before, and some of them will be highlighted in the following. On the other hand, this resulted in many new physical insights and a much deeper understanding of optimal pulses and their mechanisms. We start with the simple but still highly nontrivial case of uncoupled spin pulses, with and without relaxation, before moving on to examples of optimal control results for coupled spin systems in liquid-state NMR. For simplicity, in the following, the term pulse will denote any control pulse (i.e., RF pulse), including composite pulses, shaped pulses, and optimal-control pulses.

#### Pulses for uncoupled spins without relaxation

We begin this section with the problem of finding efficient broadband excitation, inversion, and refocusing pulses. More precisely, broadband excitation pulses are designed to rotate an initial Bloch vector from the *z* axis as close as possible to the *x* axis for a specified range of offset frequencies in a bandwidth that is large compared to a given upper bound of the pulse amplitude. Hence, such an excitation pulse is a special case of a PP pulse. Similarly, an inversion pulse is also a PP pulse that transfers *z* to −*z*, whereas a $180_{x}^{\circ }$ refocusing pulse is a universal rotation (UR) pulse that for any initial Bloch vector effects a 180° rotation around the *x* axis (in the quantum technology literature, such a $180_{x}^{\circ }$ pulse is simply known as X gate); see the Introduction and Fig. 1 for illustrative examples of the effect of robust broadband inversion and refocusing pulses.

The goal of time-optimal robust control is to find the shortest possible pulse that achieves a desired fidelity for a given maximum nominal pulse amplitude, for a given bandwidth, and for a specified range of scaling factors for the nominal pulse amplitude.

In the early 2000s, the first numerical optimal-control studies [without (*158*) and with (*236*) imposed upper limits for the nominal pulse amplitude] were conducted. This resulted in broadband excitation pulses, which were very promising and substantially exceeded the performance of the best conventional composite or shaped pulses that were known at the time. In 2004, a systematic numerical optimal-control study was published that established the performance limits of broadband excitation pulses (*80*) for bandwidths of up to five times a maximum nominal pulse amplitude of 10 kHz, for a range of pulse-amplitude scaling factors of up to ±20% and for pulse durations of up to about 700 μs (corresponding to about seven times the inverse of the maximum nominal pulse amplitude). The most important result for practical applications was that for the same bandwidth and the same maximum pulse amplitude, the obtained time-optimal broadband inversion pulses are about an order of magnitude shorter than conventional composite and shaped pulses that have comparable performance. Even further reductions of pulse durations were possible by using an adapted cost function that makes it possible to limit the spread of the phase errors of the excited Bloch vectors over the offset bandwidth by a slight (experimentally irrelevant) loss of signal amplitude (*113*).

**Fig. 7.**
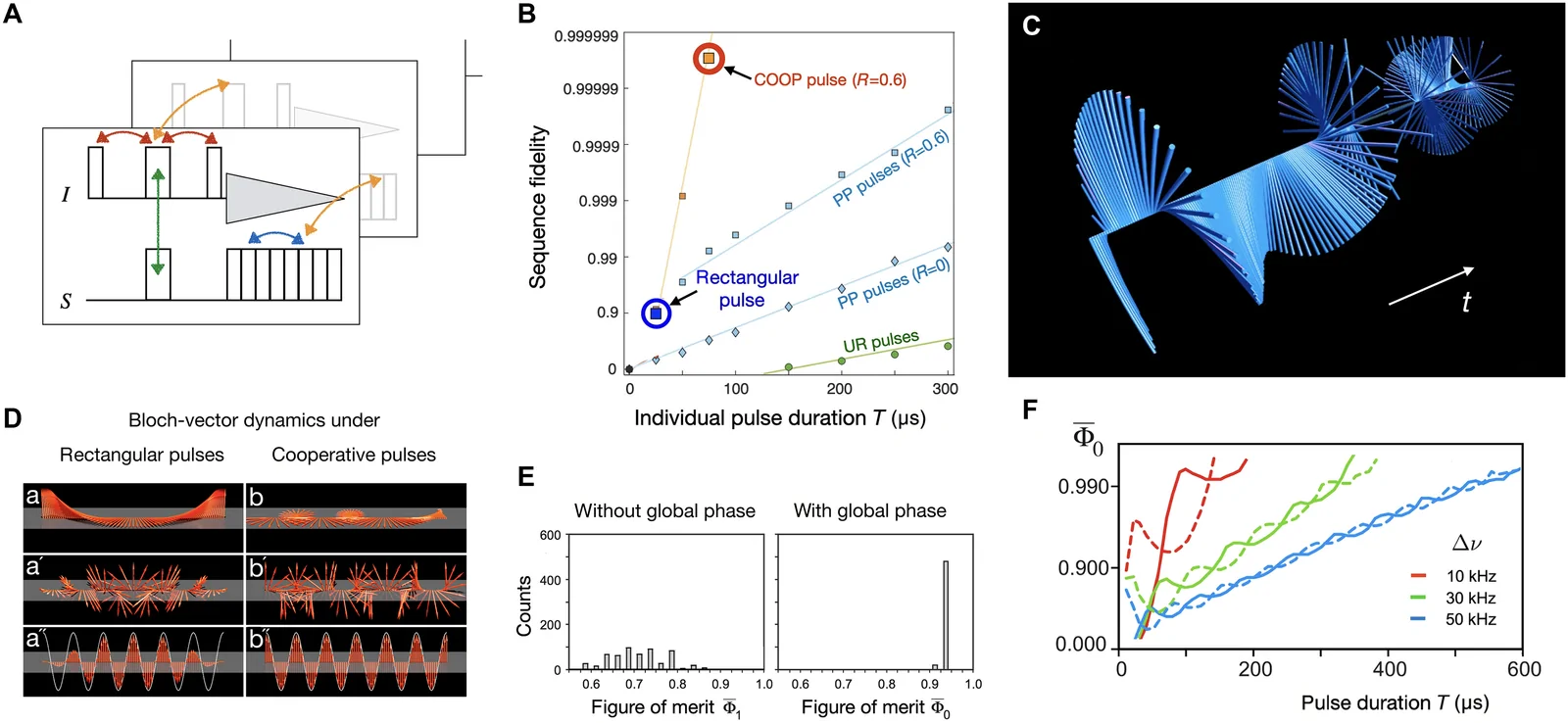
Examples of single-spin optimal-control pulse families with and without pulse cooperativity, and with and without consideration of the global phase of universal-rotation (UR) pulses. (**A**) Potential cooperativity effects between different pulses of a typical NMR experiment are indicated by arrows: between homonuclear pulses (red), within a multiple-pulse sequence (blue), between heteronuclear pulses (green), and between pulses in multiple scans (orange). (**B**) Scaling of broadband Ramsey sequence fidelity as a function of the duration *T* of the individual pulses for different pulse families. Adapted from (*117*), licensed under CC BY 3.0 (https://creativecommons.org/licenses/by/3.0/). (**C**) Ramsey sequence based on optimal control–based COOP excitation and flip-back pulses with constant amplitude and smoothly varying phase, indicated by the varying orientation of the radial bars (*119*). Adapted from (*402*) with permission. (**D**) Snapshots of the offset-dependent Bloch vector orientations are shown during a Ramsey sequence consisting of rectangular pulses (left column) or of COOP pulses (right column). Adapted from (*117*) with permission. (**E**) Histograms showing the distribution of quality factors of broadband UR(180°) pulses optimized using figures of merit, which are respectively insensitive (left panel) or sensitive (right panel) to the global phase of the pulse propagator. Adapted from (*81*) with permission from Elsevier. (**F**) Maximum quality factors reached for broadband universal UR(90°) pulses as a function of pulse duration *T* for three different offset ranges Δν [adapted from (*81*) with permission from Elsevier]. Dashed and solid curves correspond to optimizations for a target global phase factor of 1 and −1, respectively.

Furthermore, in typical multidimensional NMR experiments, pulses are not isolated but appear in combination with other pulses (see Fig. 7A). This opens the possibility to optimize these pulses in parallel to compensate each other’s imperfections without requiring the individual pulses to be perfect, leading to the concept of optimal control–enabled single-scan cooperative pulses (*117*). In the case of the frequently used Ramsey-type building block consisting of a flip-down and a flip-up 90° pulse separated by a delay, this has led to another order of magnitude reduction in the pulse duration of the individual excitation and flip-back pulses (*117*, *119*) (see Fig. 7B). The performance of the COOP pulses with a phase slope *R* = 0.6 is outstanding (red circle): Compared to a standard rectangular pulse (blue circle), it is only three times longer but provides an infidelity that is four orders of magnitude smaller. Figure 7C shows a representation of the smooth phase modulation of a COOP Ramsey sequence. Figure 7D presents snapshots of the offset-dependent orientation of the Bloch vectors (offset range: 70 kHz, pulse amplitudes: 10 kHz) during a Ramsey sequence consisting of two rectangular pulses (left column, a-a″) or consisting of two simultaneously optimized COOP (*R* = 0.6) pulses (right column, b-b″). Snapshots of the offset-dependent Bloch vectors are shown after the excitation pulse (first row) and after the second pulse before (second row) and after (third row) the application of a final zero-quantum filter. As shown in panel b″, the final COOP response perfectly matches the target Ramsey fringe pattern indicated by the white curve (*117*).

Hence, with the help of optimal control pulses, for important applications of practical interest, it became possible to reduce the duration of excitation pulses not just by one but by two orders of magnitude compared to conventional composite pulses. From a conceptional point of view, it is interesting to note that a detailed analysis (based on the offset-dependent Euler angles of the two pulses) of Ramsey-COOP pulses has led to the discovery of a novel class of pulses called ST pulses, that play a central role in Ramsey-COOP experiments, where ST denotes “Symmetric offset dependence of Euler angle α with an optional Tilt” (*117*).

In addition to single-scan COOP pulses for the Ramsey building block [e.g., in stimulated echo experiments or in excitation-evolution-flipback 2D NMR sequences (*117*)], the concept has also been successfully applied to spin-echo sequences (*118*). It turns out that the concept of single-scan pulse cooperativity is essentially identical to the concept of global pulse sequence compensation that was already introduced in the 1980s (*28*, *29*, *237*) and which can now be fully exploited using optimal-control techniques. We note in passing that the concept of optimal control–based mutual cancellation of pulse errors can also be exploited in experiments with multiple scans (see orange arrows in Fig. 7A). Such optimal control–based multiscan cooperative pulses can be viewed as a generalization of conventional phase cycling (*4*), with potential applications in MRI and quantum technology (*238*, *239*).

Optimal control–based excitation pulses have reached unprecedented bandwidths of the covered offset range. Conversely, optimal-control pulses can also provide pulses with extreme robustness with respect to pulse-amplitude scalings (*240*). However, it is also possible to simultaneously cover a very large offset range as well as a large range of pulse-amplitude scaling (*241*). The ability of numerical optimal-control algorithms to optimize thousands of pulse parameters even makes it possible to design so-called pattern pulses, which can create arbitrary excitation patterns as a function of offset frequency and pulse amplitude scaling (*71*), considerably extending the limits of conventional approaches to design frequency offset-selective pulse responses. Both from a theoretical and practical perspective, it is interesting to note that the problem of broadband spin excitation can be mapped to the simpler problem of controlling an ensemble of harmonic oscillators, as demonstrated in (*242*).

In many cases, it is sufficient [and, for some applications, even desirable (*114*)] to create broadband excitation pulses that bring the Bloch vector into the equatorial plane of the Bloch sphere with a phase (corresponding to the angle between the *x* axis and the final Bloch vector) that increases linearly as a function of offset. The detailed performance of such excitation pulses with linear target phase was systematically studied as a function of the phase slope (*114*). Positive phase slopes correspond to an ideal excitation pulse with a defined additional offset evolution period, whereas negative slopes correspond to prefocused pulses, which result in the refocusing of all Bloch vectors at the end of a well-defined delay after the end of the pulse (*114*), eliminating the need for an additional refocusing pulse to form a spin echo. In this context, an intriguing question is, by how far can the echo be pushed away from the end of the pulse? Intuitively, it seemed to be clear that the largest achievable delay between the end of the pulse and the echo should be limited by the pulse duration. While this is true for pulses with a flip angle of 90°, it turns out that for prefocused pulses with a smaller flip angle, it is in fact possible to push the echo much further away from the end of the pulse (*115*), with interesting applications in MRI (*115*, *203*). While in (*114*) the target slope of the offset-dependent linear phase was varied systematically to study in detail how the achievable pulse fidelity depends on the slope, in practice, it is also possible to optimize the slope during the optimization of the excitation fidelity to achieve the best possible performance. This yields very short excitation pulses with linear offset-dependent phase of the final Bloch vector, minimizing spectral artifacts in the presence of large homonuclear spin-spin couplings (*243*).

Even shorter excitation pulses (or larger bandwidths for the same pulse duration) can be obtained if the only condition is to bring the Bloch vector in the offset frequency range of interest to the equator of the Bloch sphere, without any constraints for the offset-dependent phase of the final Bloch vector. In the nomenclature of (*28*), such excitation pulses are called class B3 pulses. Such optimal-control pulses have been designed for applications in EPR spectroscopy (*244*) and also for ultrabroadband 1D and 2D NMR applications (*245*).

Beyond broadband excitation pulses, optimal control–based broadband *z* to −*z* inversion pulses have also been systematically studied in (*246*). However, compared to the excitation problem, broadband inversion is relatively easy to achieve, and conventional approaches already provided very good pulses, such that the remaining gains of optimal control are notable but less spectacular than in the case of broadband excitation pulses. For a detailed comparison of optimal control and conventional composite and shaped pulses, see (*80*). In addition to broadband excitation and inversion pulses with constraint maximum pulse amplitudes, systematic studies were also performed for these pulses with constraints of the average pulse power and total energy of the pulses (*246*).

Optimal control pulses were optimized not only for excitation and inversion pulses but also for the important class of broadband universal 90° and 180° rotation (UR) pulses; see (*81*) for a detailed study and a thorough discussion of interesting properties, such as preferred symmetries of these pulses. This symmetry analysis revealed an unexpectedly simple construction principle for assembling a UR pulse with a desired rotation angle from special PP pulses (*32*). In particular, it turns out that this construction principle makes it possible to assemble a broadband 180° UR pulse based on a broadband *z* to *x* PP pulse.

In (*247*) it was shown how this construction principle can be used to eliminate phase errors of broadband refocusing pulses and how relaxing conservative limits on peak pulse amplitude for short time periods can help to boost pulse performance without causing hardware problems. However, as pointed out in (*81*), depending on the applications, it is still highly useful to be able to efficiently optimize UR pulses directly because pulses based on the construction principle result in very good UR pulses but not always the best possible pulses. Therefore, it is still highly useful to be able to efficiently optimize UR pulses directly.

For the task to optimize robust UR pulses, it is important to understand the decisive role of the global phase of the unitary operator (also known as the propagator) that represents the desired UR rotation. This subtle but important effect has been analyzed and discussed (*81*, *201*), but it does not seem to have entered common awareness, and this review would not be complete without addressing this point: As the global phase of SU(2) pulse propagators (*248*) has no effect on experimentally measurable observables, it is generally regarded as irrelevant, and it is typically assumed that it can simply be ignored. Therefore, it is tempting to choose a cost function in which the global phase is eliminated to “simplify” pulse optimizations. However, as illustrated in Fig. 7E, this approach can result in a large number of suboptimal local maxima of the FOM, which severely reduces the chance to find high-fidelity pulses. [Note that the same problem occurs if UR pulses are not optimized in SU(2) but in SO(3), i.e., if instead of complex unitary 2 × 2 matrices, real-valued orthogonal 3 × 3 rotation matrices are optimized, which also do not carry the global phase information.] Fortunately, most of the suboptimal solutions are eliminated if a cost function is chosen that is sensitive to the global phase of the propagator and if the same target value of the global phase factor is chosen for all offset frequencies (*81*). [For definitions of UR cost functions based on SU(2) propagators—with and without accounting for the global phase—and for their relation with cost functions based on SO(3) rotation operator or on the simultaneous optimization of three orthogonal PP transfers, see (*112*) and (*81*).] This effect is a result of the fact that in broadband GRAPE optimizations of UR pulses, a large number of cost function evaluations for different offset frequencies are averaged to yield the total cost function that is optimized. Hence, if the global phase is not taken into account, different parts of the offset range may converge to different global phase factors, namely, ±1 in SU(2). However, this leads to a poor value of the total cost function (and hence results in an undesirable local optimum) because within the control-parameter space under consideration, there is no continuous path connecting an SU(2) propagator *U* to −U while avoiding both *I* and −I, resulting in an unfavorable value of the local FOM. Enforcing a well-defined single global phase factor for the desired offset bandwidth avoids these local maxima. Except for the case of UR(180°) pulses, the optimization should be repeated for the two possible global phase factors ±1 to find the best pulse, which is a negligible price to pay for the elimination of a large number of local maxima. For more details, see (*81*, *201*). This is a notable example of how the spinor property (*248*) of half-integer-spin representations of SU(2), in which a 360° rotation is represented by −*I* rather than *I*, can be leveraged to enable optimal control algorithms to find substantially better pulses for practical applications.

As shown in (*81*), for increasing pulse durations, optimal broadband pulses alternate between pulse families with global phase factors of 1 and −1 in a systematic way (see Fig. 7F). The role of the global phase of propagators is more intricate in coupled spin systems, as in this case, more than two possible global phase factors exist, which correspond to pulse families with different minimal durations to achieve a desired fidelity (*249*). For the relatively simple—but still highly nontrivial—case of time-optimal control for a specific offset frequency (i.e., without offset robustness) and bounded pulse amplitude, the effect of the global phase of the propagator has been analytically elucidated using a detailed geometric optimal control analysis (*165*).

For MRI applications, slice-selective, highly broadband UR refocusing pulses were optimized, which create substantially smaller chemical-shift displacement artifacts (*250*). It is also possible to roughly limit the bandwidth of optimal control pulses indirectly by using low-pass filters (*201*, *251*, *252*) on the actual pulse shape. For applications in protein spectroscopy, multiple-band–selective pulses were developed for homonuclear decoupling without Bloch-Siegert shift (*253*).

To simplify the use of optimal control pulse sequences in liquid-state NMR, in (*254*), a first set of highly robust and high-fidelity PP 90° excitation and PP 180° inversion, UR 90° and UR 180° refocusing pulses was optimized for ^1^H and for ^13^C pulses, which can replace all 90° and 180° hard pulses in a given pulse sequence for improved performance. With a focus on applications to small to medium-sized molecules with relatively long relaxation times, each pulse has the same duration of 1 ms, such that they can be easily stacked and assembled. More recently, extended sets of optimal control pulses were designed (*255*, *256*), which is complemented by a computational framework with a highly efficient GRAPE implementation for on-the-fly pulse optimizations (*256*). Furthermore, sets of optimal control pulses for ultrahigh magnetic fields of 28 T corresponding to a proton Larmor frequency of 1.2 GHz were developed (*257*, *258*).

In the “Methods” section, the use of the PMP was illustrated for a seemingly simple exemplary toy problem how to transfer a magnetization vector (i.e., the Bloch vector) as fast as possible (i.e., in minimum time) from the *x* axis to the *y* axis if the pulse amplitude cannot exceed a given maximum value (i.e., if the control amplitude is bounded). Our intuition (which turns out to be correct in this case) tells us that we should use the full pulse amplitude for the entire time, and hence, we can reduce the problem to the task of finding the optimal pulse phase as a function of time. We can quickly come up with two quite obvious solutions that realize this PP *x* to *y* control transfer. One solution could be to use a constant pulse phase of 45°, i.e., to rotate the magnetization vector around the unit vector $\left(\vec{x}+\vec{y}\right)/\sqrt{2}$. Note that the magnetization vector must complete a rotation of 180° around this axis; i.e., the duration of this approach is simply the duration $T_{180}$ of a 180° pulse with the maximum allowed pulse amplitude.

The second quite obvious solution is to first rotate the magnetization vector from the *x* axis to the *z* axis by a $90_{-y}^{\circ }$ pulse, followed by a $90_{-x}^{\circ }$ pulse that flips the spin down to the *y* axis. This solution has the duration of two 90° pulses, i.e., it also takes the duration $T_{180}$ of a 180° pulse. In light of these considerations, it might be tempting to assume that the minimum time for the PP(x→y) problem is $T_{180}$. However, this is the point where simple intuition ends and where the PMP comes to the rescue: As shown in the “Methods” section, the optimal solution of this control problem has a duration of only $\sqrt{3}/2\;T_{180}\approx 0.87\;T_{180}$! Furthermore, the PMP formalism also allows us to analytically derive the corresponding time-dependent phase of the optimal pulse. It is this power of the PMP that makes it possible to analytically analyze many nontrivial optimal control problems and to prove global optimality. Many empirical findings based on numerical optimal control algorithms were complemented by important analytical geometric optimal control studies of broadband and selective PP and UR pulses based on the PMP (*79*, *169*, *175*, *259*–*262*). For example, an interesting and unexpected finding in systematic numerical GRAPE optimizations of amplitude-limited broadband pulses was the fact that for short pulse durations, the optimized controls correspond to simple phase-alternating composite pulses, but at a critical pulse duration, a transition occurs to pulses with smooth phase modulation (*80*, *81*). This empirical phenomenon could be reproduced in analytical studies of model systems and traced back to the fact that optimal first-order offset compensation only requires phase-alternating pulses, whereas higher-order corrections require smooth phase modulation (*262*). Hence, if pulses are numerically optimized for durations that are longer than the minimum time required for second-order offset compensation, smooth phase modulation is also found for GRAPE pulses.

#### Uncoupled spins with relaxation

Let us now revisit the problem of broadband excitation in the presence of relaxation, i.e., with nonnegligible longitudinal and transverse relaxation rates $1/T_{1}$ and $1/T_{2}$. Intuitively, it seems to be clear that the best solution in the presence of relaxation should simply be the shortest possible solution in the absence of relaxation. Therefore, it may come as a surprise that often relaxation-optimized pulses can in fact be longer than the time-optimal solutions in the absence of relaxation. This is also the case for broadband excitation pulses that accommodate relaxation, as clearly demonstrated both theoretically and experimentally in (*199*).

Geometric optimal control studies of uncoupled spins in the presence of relaxation have focused on the problem of how to reach the center of the Bloch ball in minimum time. This problem of (complete) saturation of spins has already been introduced in the “Basics of Spin Control” section and has led to the discovery of the previously unknown magic plane in the Bloch sphere, which plays a central role in the analytical and numerical analysis of this control problem (*84*, *85*, *263*, *264*). In the course of these studies, it was also demonstrated that the time-optimal saturation problem can even be solved in the presence of relaxation and radiation damping (*75*, *86*, *216*). In subsequent studies (*15*, *130*, *215*–*219*, *265*), it was analyzed analytically and numerically how to saturate one spin species (with given $T_{1}^{A}$ and $T_{2}^{A}$ values) while maximizing the length of the magnetization vector of a second spin species (with different relaxation times $T_{1}^{B}$ and $T_{2}^{B}$) if both spins are subject to the same control pulse (or sequence of pulses) in an optimal way. In liquid-state NMR, such pulses are useful for suppressing signals from irrelevant compounds in a mixture while maximizing the signals from compounds of interest. In MRI, such pulses can be used to enhance imaging contrast. Further analytical optimal control studies focused on the important problem of maximizing the achievable signal-to-noise ratio per unit time (*124*–*126*).

#### Coupled spins without relaxation

By the end of the 1990s, efficient analytical and numerical tools had been developed to establish the maximum possible transfer efficiency $\eta_{\text{max}}$ for any unitary state-to-state transfers between *Hermitian* operators (e.g., from $I_{1x}$ to $I_{2x}$) (*106*) and also between non-Hermitian operators [e.g., from $I_{1}^{-}=\left(I_{1x}-iI_{1y}\right)$ to $I_{2}^{-}=\left(I_{2x}-{\mathrm{iI}}_{2y}\right)$] (*109*, *111*). This was a major achievement, because it made it possible to compare the efficiency of the best known pulse sequences for a desired transfer with the corresponding $\eta_{\text{max}}$ to see if the physical efficiency limit has already been achieved or if there is still room for improvement (*266*). However, at that time, most numerical methods for optimizing pulse sequences—including downhill simplex, genetic algorithms, evolutionary algorithms, and simulated annealing—were largely heuristic and often required extensive computational resources and could handle only a small number of pulse-sequence parameters. Furthermore, it was still unclear whether and how one could determine the minimum time $\tau^{*}$ to reach $\eta_{\text{max}}$ and the maximum achievable transfer efficiency as a function of transfer time *T* for $0<T<\tau^{*}$.

Analytical optimal control methods (*166*, *176*, *267*) and optimal control–based numerical algorithms, such as GRAPE (*112*), proved to be exactly the right tools to address these open questions. The curve η(T) as a function of the pulse duration *T* is called the TOP curve (short for time-optimal pulse curve) from which also the maximum achievable transfer efficiency $\eta_{\text{max}}$ and the minimum time $\tau^{*}$ to achieve the maximum transfer can be obtained. To gain a deeper understanding of the fundamental physical limits of the achievable transfer efficiency, the analytical form of such TOP curves is highly desirable. However, the analytical description of the TOP curves remained elusive until ideas from geometric optimal control were brought to bear on NMR: In the seminal paper (*176*) on time-optimal control in spin systems, it was shown how the concept of the canonical Cartan decomposition (KAK) of SU(4) into local and nonlocal components can be used to find the minimum time for any desired unitary operator to be experimentally realized in two-spin systems (e.g., consisting of two heteronuclear spins) and how to realize it in terms of (fast) selective spin rotations and periods of (slow) coupling evolution. These results lastly made it possible to derive analytical TOP curves η(T), the optimal pulse sequence for any duration *T*, the maximum achievable transfer efficiency $\eta_{\text{max}}$, and the minimum time $\tau^{*}$ to achieve it.

**Fig. 8.**
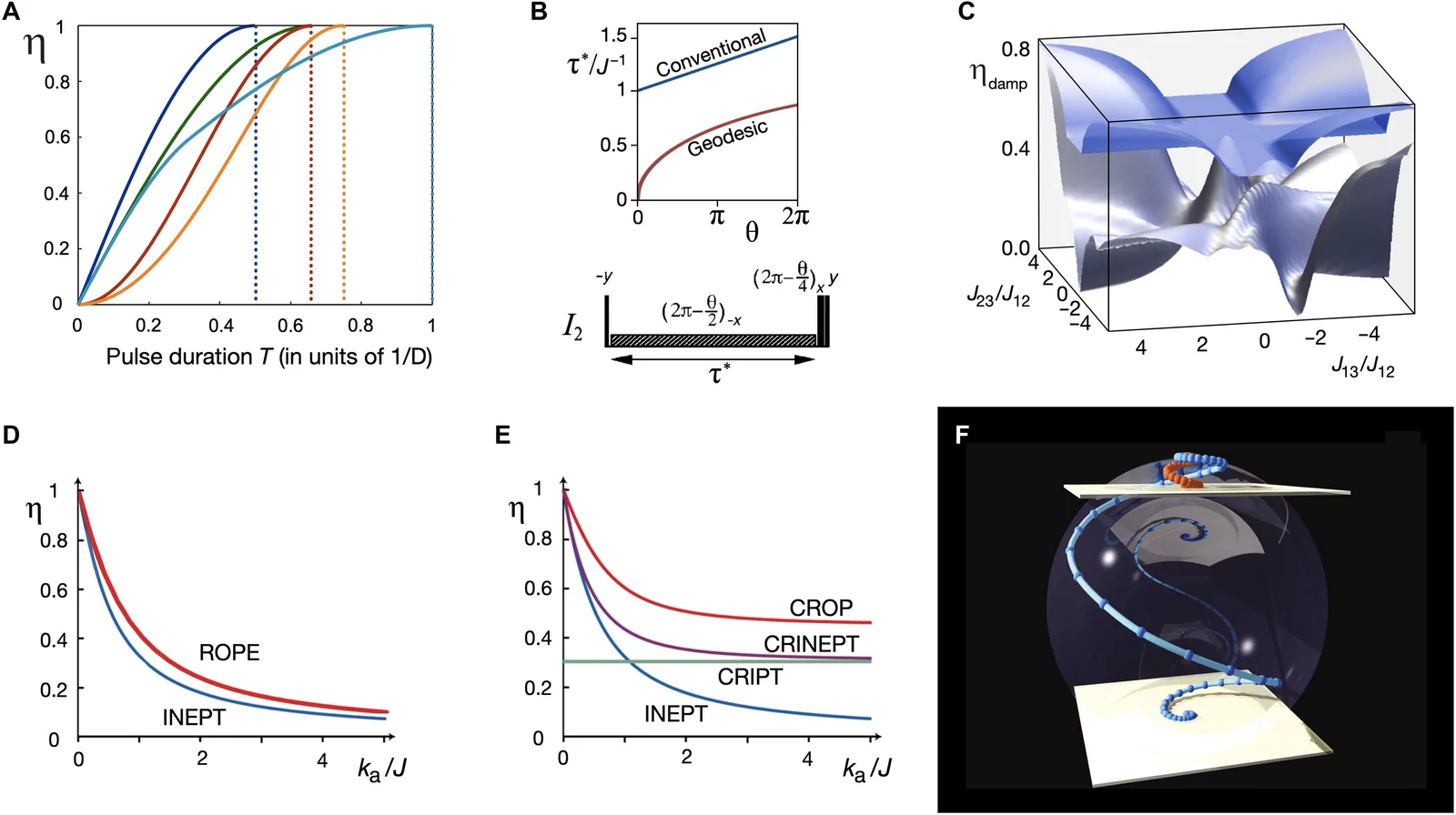
Examples of time-optimal and relaxation-optimized control pulses for multispin systems. (**A**) Analytical time-optimal pulse (TOP) curves showing the maximum achievable transfer efficiency as a function of pulse duration *T* for the case of two dipolar coupled spins. Adapted from (*267*) with permission from Elsevier. The curves represent the transfers $I_{x}\to S_{x}$ (red), $I^{-}\to S^{-}$ (orange), $I_{x}\to 2I_{z}S_{x}$ (dark blue), $I^{-}\to 2I_{z}S^{-}$ (light blue), $I_{x}S_{\beta }\to I_{\beta }S_{x}$, and $I^{-}S_{\beta }\to I_{\beta }S^{-}$ (green). (**B**) Analytical TOP curves of a conventional pulse sequence (*273*) and the time-optimal geodesic pulse sequence (*166*) for the generation of a propagator $U=\text{exp}\left(-\mathrm{i\theta}I_{1z}I_{2z}I_{3z}\right)$ [adapted from (*166*) with permission]. The geodesic pulse sequence applied to spin $I_{2}$ is shown below [adapted from (*166*) with permission]. (**C**) Efficiency of polarization transfer $I_{1z}\to I_{2z}$ in a homonuclear three-spin system as a function of the relative coupling constants $J_{13}$/$J_{12}$ and $J_{23}$/$J_{12}$ for numerically optimized pulses (blue surface) compared to TOCSY transfer (gray surface) (*42*, *281*). (**D**) Relaxation-optimized transfer from in-phase to antiphase coherence. In the absence of cross-correlated relaxation (i.e., in the case of a vanishing cross-correlated relaxation rate $k_{c}$), a comparison of the transfer efficiency η of the conventional INEPT (*283*) and of the optimal control–based ROPE (*87*) sequence is shown as a function of the ratio between the auto-correlated relaxation rate $k_{a}$ and the *J* coupling. Both INEPT and ROPE are only driven by *J* coupling. Adapted from (*87*) with permission from Elsevier. (**E**) For the same transfer as in (D), even larger transfer efficiencies $\eta \left(k_{a}/J\right)$ can be achieved for nonvanishing $k_{c}$ rates. For the case of $k_{c}/k_{a}=0.75$, η curves are shown for INEPT (uses only *J* transfer), CRIPT (*285*) (only $k_{c}$ driven), CRINEPT (both *J* and $k_{c}$ driven), and the optimal control–based CROP (*88*) sequence (optimally driven by both *J* and $k_{c}$). Adapted from (*88*) with permission, Copyright (2003) National Academy of Sciences, USA. (**F**) Optimal CROP trajectories of the expectation values of the two doublet components $\vec{I}S^{\alpha }$ (red curve) and $\vec{I}S^{\beta }$ (blue curve) for the ratio $k_{c}/k_{a}=0.95$. The distance between the two horizontal planes intersecting the final point of the red and blue trajectories corresponds to the achieved transfer efficiency $\eta_{\text{CROP}}$.

A relatively simple example is the coherence-order–selective in-phase transfer ($S^{-}$ to $I^{-}$) in a heteronuclear spin system consisting of two spins *S* and *I* with a coupling constant *J*. For this case, it is well known that the minimum time for the full transfer is $\tau^{*}=1.5/J$ (*41*), which is achieved by a heteronuclear isotropic mixing sequence. Hence, to achieve the best possible transfer efficiency for $\tau <\tau^{*}$, it appears to be reasonable to assume that the best transfer sequence would still be heteronuclear isotropic mixing. However, as shown in (*176*) [and as experimentally confirmed in (*268*)], this is not the case and the optimal solution achieves up to 12.5% larger transfer efficiency. For many transfers of both practical and theoretical interest, the detailed analytical forms of TOP curves (and corresponding plots) are explicitly summarized and plotted for different coupling Hamiltonians in (*267*). Figure 8A shows examples of characteristic TOP curves for the case of two homonuclear spins with a dipolar coupling Hamiltonian.

The idea of Cartan decomposition can be iteratively generalized beyond systems consisting of more than two spins (*177*). This approach can still create efficient control sequences, which makes it interesting for quantum compilation (*177*, *269*) (see also the “Quantum technology approaches” section). For two coupled spins, the space of nonlocal propagators, obtained by removing all selective single-spin rotations from SU(4), is a so-called Riemannian symmetric space. (This means that the geometry is exceptionally regular: The coupling Hamiltonian generates geodesics that can be characterized analytically, and every nonlocal operation can be expressed in a simple canonical form.) However, for more than two spins, this geometric regularity disappears: The corresponding quotient space no longer has the symmetry properties required for such a canonical reduction, and the time-optimal control problem becomes substantially more complex, such that the generalized Cartan decomposition usually does not result in a time-optimal solution (*177*).

For the case of three coupled heteronuclear spins, it was shown how analytical time-optimal coherence transfer sequences (*166*, *270*) can be derived using the concept of so-called sub-Riemannian geodesics (*166*, *271*, *272*). This results in substantially shorter pulse sequences compared to conventional approaches. For example, in a linear three-spin chain with equal couplings $J_{12}=J_{23}$ between neighboring spins and $J_{13}=0$, the coherence-order–selective in-phase transfer $I_{1}^{-}\to I_{3}^{-}$ takes only $\sqrt{3}/2\approx 86.6\%$ and the implementation of a SWAP(1,3) operation takes only $1/\sqrt{3}\approx 57.7\%$ of the duration of the conventional approach. The most marked gain can be found for the experimental simulations of the time evolution under an effective trilinear coupling term (also known as a three-body or three-particle interaction) on a quantum computer, where, in the limit of very small evolution steps, the ratio between the durations of the time-optimal realization (*166*, *272*) and the conventional method (*273*) tends to 0; i.e., in this limit, the time-optimal approach becomes infinitely faster (see Fig. 8B).

Further in-depth analytical (*167*, *274*, *275*) and numerical (*254*, *257*, *276*–*280*) studies of time-optimal quantum gates, polarization transfers, generation of multiple-quantum coherence, heteronuclear pairs of simultaneously applied refocusing pulses with *J*-coupling evolution taken into account, and of bilinear filter elements have been performed in systems consisting of up to five spins.

The GRAPE algorithm makes it possible as well to numerically explore the physical limits of the maximum achievable transfer efficiency η(T) for any desired coherence or polarization transfer task (defined by the initial operator and the desired target operators) and also provides the pulse sequence that achieves this efficiency (*112*). The previously derived analytical TOP curves provide ideal benchmarks, which are closely matched by the GRAPE-based TOP curves (*112*). However, GRAPE can also handle more complex problems for which currently no analytical solutions are known, such as the optimal design of robust polarization-transfer sequences that work for a range of offset frequencies and pulse amplitude scalings and in the presence of experimental pulse constraints.

For the general case of three-spin systems with arbitrary coupling constants, the efficiency of polarization transfer was systematically studied (*277*) using the GRAPE algorithm and compared to the TOCSY transfer efficiency as a function of the relative coupling constants $J_{13}$/$J_{12}$ and $J_{23}$/$J_{12}$ (*42*, *281*) (see Fig. 8C). For larger spin systems consisting of up to five spins, further GRAPE-based studies for the most efficient transfer of polarization (*278*) and the generation of maximum multiple-quantum coherence (*276*) have been performed. Last, a new analytical approach for efficient (but not time-optimal) coherence transfer through linear spin chains of arbitrary length has been proposed (*166*).

#### Coupled spins with relaxation

A major achievement of geometric optimal control was the thorough analysis and understanding of the physical limits of polarization and coherence transfer in heteronuclear two-spin systems, both in the absence (*88*, *162*) and in the presence (*88*, *89*, *282*) of cross-correlated relaxation. This is of particular interest for multidimensional liquid-state NMR spectroscopy of large biomolecules, where transverse relaxation rates are much larger than longitudinal relaxation rates and can exceed the size of the *J* coupling. This fact can be exploited in relaxation-optimized coherence transfer schemes by storing coherence as longitudinal polarization and only bringing it gradually into the transverse plane for the transfer in a well-defined way, instead of using hard 90° pulses. Contrary to the common belief that coherence transfer in the presence of relaxation should be as fast as possible, suitably designed longer pulses can, in fact, achieve higher transfer efficiencies.

Conventional INEPT-type (Insensitive Nuclei Enhanced by Polarization Transfer) transfer schemes (*283*) solely rely on *J* coupling–driven transfer of in-phase coherence (e.g., $I_{x}$) to antiphase coherence (such as $2I_{x}S_{z}$ or $2I_{y}S_{z}$), which, in the absence of relaxation, has a transfer efficiency η of 100% and takes a transfer time of 1/(2J), where *J* is the coupling constant between the two spins. With increasing transverse relaxation rate, the INEPT transfer efficiency rapidly decreases, even if the transfer time is adjusted to $t_{\text{INEPT}}^{*}=1/\left(\mathrm{\pi J}\right){\text{cot}}^{-1}\left(k_{a}/J\right)$ for the best compromise between *J* transfer and relaxation loss due to the transverse auto-correlated relaxation rate $k_{a}$ (see Fig. 8D). Could it be possible to achieve better transfer efficiencies using other transfer schemes, or is using the INEPT transfer scheme already the best one can do (in the absence of cross-correlated relaxation)? In the early 2000s, the answer to this important question was unknown; determining the physical limit of coherence-transfer efficiency in the presence of relaxation was still an open problem.

In a seminal paper (*87*), this problem was lastly solved fully analytically (i.e., without any numerical optimization) using geometric optimal control theory via the Hamilton-Jacobi-Bellman equation (*284*) in 2003. The corresponding optimal transfer scheme is called ROPE (short for relaxation optimized pulse element). An analytical formula for the maximum ROPE transfer efficiency was derived, which turned out to have an unexpectedly simple form: $\eta_{\text{ROPE}}=\sqrt{1+\xi^{2}}-\xi$, where $\xi =k_{a}/J$ is the ratio between the transverse auto-correlated relaxation rate $k_{a}$ and the *J* coupling (see Fig. 8D). In the limit of large ξ, the ratio $\eta_{\text{ROPE}}/\eta_{\text{INEPT}}$ approaches a value of e/2=1.359; i.e., compared to INEPT, an up to 36.9% sensitivity gain can be achieved by using ROPE instead. For the optimal in-phase to in-phase transfer, e.g., $I_{x}\to S_{x}$, a factor of up to ${\left(e/2\right)}^{2}=1.847$ larger efficiency is found when using the ROPE scheme compared to refocused INEPT, corresponding to an improvement of the signal-to-noise ratio by almost 85%. The optimal ROPE pulse sequence can be analytically derived based on the optimal transfer trajectory, which is characterized by the condition that the ratio $\langle 2I_{y}S_{z}\rangle /\langle I_{x}\rangle$ needs to be held at a specific constant value throughout the transfer, and this constant value unexpectedly just happens to be $\eta_{\text{ROPE}}$.

In 2003, the optimal polarization-transfer problem could also be resolved for the case of a nonvanishing cross-correlated relaxation rate $k_{c}$ (resulting from DD-CSA cross-correlated relaxation, where DD is short for dipole-dipole and CSA stands for chemical shift anisotropy). The best conventional approach that is solely $k_{c}$ driven is CRIPT (Cross-Relaxation Induced Polarization Transfer) (*285*), which is more efficient than INEPT for $k_{a}>J$ (see Fig. 8E). The hybrid CRINEPT (Cross-Relaxation Induced INEPT) (*286*) uses both *J* and $k_{c}$ driving and exceeds the transfer efficiencies of INEPT and CRIPT for all values of $k_{a}/J$ (see Fig. 8E). However, as shown both theoretically and experimentally (*88*), the CRINEPT transfer efficiency can still be substantially further improved by the analytically derived optimal control–based CROP (cross-correlated relaxation optimized pulse) sequence. The derived analytical expression for the optimal transfer efficiency is $\eta_{\text{CROP}}=\sqrt{1+\zeta^{2}}-\zeta$, with $\zeta =\sqrt{\left(k_{a}^{2}-k_{c}^{2}\right)/\left(J^{2}+k_{c}^{2}\right)}$. In the limit where the cross-correlated relaxation rate $k_{c}$ becomes identical to the auto-correlated relaxation rate $k_{a}$, the CROP sequence reaches a transfer efficiency of 100%.

Because of the need to selectively invert the narrower of the two spin *I* doublet components (cf. Fig. 8F), it was initially not clear if a broadband implementation of the CROP sequence is possible because conventional refocusing methods for offset evolution eliminate either *J* coupling or cross-correlation effects. However, the discovery (*89*) of a refocusing element, the so-called STAR (specific trajectory-adapted refocusing) echo, indeed makes it possible to refocus the effect of offset evolution without reducing the driving effects of *J* and $k_{c}$ in the CROP approach.

For applications where $k_{c}$ is much larger than *J* (such as in very large biomolecules), it is also interesting to consider the best possible transfer efficiency that is achievable if only $k_{c}$ driving is used, as in the conventional CRIPT sequence. As shown in (*282*), the CRIPT transfer can be substantially further improved by using the optimal control–based TROPIC (transverse relaxation optimized polarization transfer induced by cross-correlation) sequence. This transfer scheme was successfully implemented for ^1^H-^15^N coherence transfer in a study of the large 800-kDa homo-tetradecameric protein GroEL, where experimentally an average sensitivity enhancement of 20 to 25% was found compared to CRIPT (*282*).

For polarization transfer along a spin chain, the efficient transfer of spin order is an important building block. For a chain of three spins, the spin-order transfer $2I_{1z}I_{2z}\to 2I_{2z}I_{3z}$ was analyzed and optimized in the presence of fast transverse auto-correlated relaxation rates (*163*). The resulting SPORTS-ROPE (spin order transfer with relaxation optimized pulse element) element provides a substantially improved transfer efficiency compared to the conventional concatenated INEPT scheme.

### Solid-state NMR spectroscopy

Relative to liquid-state NMR, solid-state NMR is complicated by, and at the same time blessed with, the direct presence of anisotropic nuclear spin interactions. The duality of this aspect is that, left unaltered, anisotropic nuclear spin interactions may broaden spectra beyond interpretation, while the same interactions may carry highly detailed and important information about the structure, dynamics, and interactions of molecules. Controlling these two aspects optimally has triggered the development of hardware with MAS now reaching rotation frequencies in the 160-kHz range using 0.4-mm rotors and spin engineering of pulse sequences that selectively turn off and on (often referred to as recoupling) anisotropic nuclear spin interactions. The latter has, to a large extent, been driven by effective (or average) Hamiltonian theory introduced to solid-state NMR by Haeberlen and Waugh in the 1960s (*20*) and later advanced to cope more efficiently with high-order perturbations (*50*) and even present in exact form, as exact effective Hamiltonian theory (EEHT) (*51*, *287*). Lately, single-spin vector effective Hamiltonian theory has proven useful in such spin engineering (*288*, *289*). The challenge of optimizing experiments under consideration of many (anisotropic) nuclear spin interactions, sample spinning, selective decoupling, and recoupling in multiple-dimensional experiments for applications in sciences spanning from materials science to structural biology renders spin engineering in solid-state NMR a wonderful playground for optimal control capable of handling a large number of variables in an optimization process.

To illustrate the complexity, yet the opportunities, the nuclear spin Hamiltonian may, in the context of solid-state NMR, be described in the high-field approximation by the Hamiltonian

$$H_{\lambda }\left(t\right)=C^{\lambda }\left\{{\left(R_{0,0}^{\lambda }\right)}^{L}T_{0,0}^{\lambda }+{\left(R_{2,0}^{\lambda }\right)}^{L}T_{2,0}^{\lambda }\right\}$$ (2)

where λ represents chemical shielding (CS), dipole-dipole (D), and quadrupolar coupling (Q) interactions; *C* denotes fundamental constants; and *R* and *T* represent irreducible tensor operators describing spatial and spin dependencies, respectively. The spatial tensor is typically described in its principal axis frame (P), transformed via Wigner rotations (*D*; and reduced elements *d*) via the rotor frame (*R*) to the laboratory frame (*L*) as

$${\left(R_{2,0}^{\lambda }\right)}^{L}=\sum_{m^{\prime },{m^{\prime }}^{\prime }}{\left(R_{2,{m^{\prime }}^{\prime }}^{\lambda }\right)}^{P}D_{{m^{\prime }}^{\prime },m^{\prime }}^{\left(2\right)}\left(\Omega_{\mathrm{PR}}^{\lambda }\right)d_{m^{\prime },0}^{\left(2\right)}\left(\beta_{\mathrm{RL}}\right)e^{-im^{\prime }\omega_{r}t}$$ (3)

where $\Omega_{\mathrm{PR}}^{D}$ represents Euler angles transforming the tensor from *P* to *R* (powder angles), $\beta_{\mathrm{RL}}$ denotes the angle between the rotor and the *z* axis in *L*, and $\omega_{r}$ indicates the sample spinning frequency. For further details, we refer the reader to more general descriptions of solid-state NMR, e.g., in (*191*, *290*, *291*).

Taking the dipolar coupling as an example, we obtain

$$H^{D}\left(t\right)=\sum_{m=-2}^{2}\omega_{D}^{\left(m\right)}e^{\mathrm{im}\omega_{r}t}\left(3I_{\mathrm{iz}}I_{\mathrm{jz}}-I_{i}\cdot I_{j}\right)$$ (4)

with $\omega_{D}^{\left(m\right)}=b_{\mathrm{ij}}D_{0,-m}^{\left(2\right)}\left(\Omega_{\mathrm{PR}}^{D}\right)d_{-m,0}^{\left(2\right)}\left(\beta_{\mathrm{RL}}\right)$ and $b_{\mathrm{ij}}$ being the dipole-dipole coupling. Under MAS conditions, we exploit that $d_{0,0}^{\left(2\right)}\left(\beta_{\mathrm{RL}}\right)=\frac{1}{2}\left[3{\text{cos}}^{2}\left(\beta_{RL}\right)-1\right]$ vanishes upon setting $\beta_{\mathrm{RL}}=54.7^{{}^{\circ}}$, leading to $\omega_{D}^{\left(0\right)}=0,\omega_{D}^{\left(1\right)}=\omega_{D}^{\left(-1\right)*}=\frac{1}{2\sqrt{2}}b_{\mathrm{ij}}s_{2\beta_{\mathrm{PR}}}e^{i\gamma_{\mathrm{PR}}}$, and $\omega_{D}^{\left(2\right)}=\omega_{D}^{\left(-2\right)*}=-\frac{1}{4}b_{\mathrm{ij}}s_{\beta_{\mathrm{PR}}}^{2}e^{i2\gamma_{\mathrm{PR}}}$.

This reveals a key element in high-resolution MAS solid-state NMR detecting signals in time periods where the anisotropic interactions are averaged by MAS (through the $m\omega_{r}t$ dependency), and using so-called “recoupling” to reintroduce anisotropic interactions in other periods. The latter can be used to transfer polarization between spins or extract structural parameters from dipole-dipole coupling, anisotropic shielding, or quadrupolar coupling parameters. Notably, dipolar recoupling uses RF irradiation in synchrony with sample revolution to induce spin modulations matching (with opposite sign) one or more of the spatial $m\omega_{r}t$ modulations, thereby canceling the time dependency in Eqs. 3 and 4 and thereby averaging. This forms a very nice class of experiments, where macroscopic manipulation (represented by *R*) of the sample couples directly with the dynamics of the spins (represented by *T*). This phenomenon was initially found as so-called rotational resonance (*60*) spectral broadening and in simple “continuous wave”–like irradiation schemes (*59*, *61*) forming the starting point for so-called symmetry-based recoupling (*58*, *62*). A large variety of advanced dipolar recoupling experiments has been designed, as reviewed in (*290*, *291*), mainly using such effective Hamiltonian methods.

**Fig. 9.**
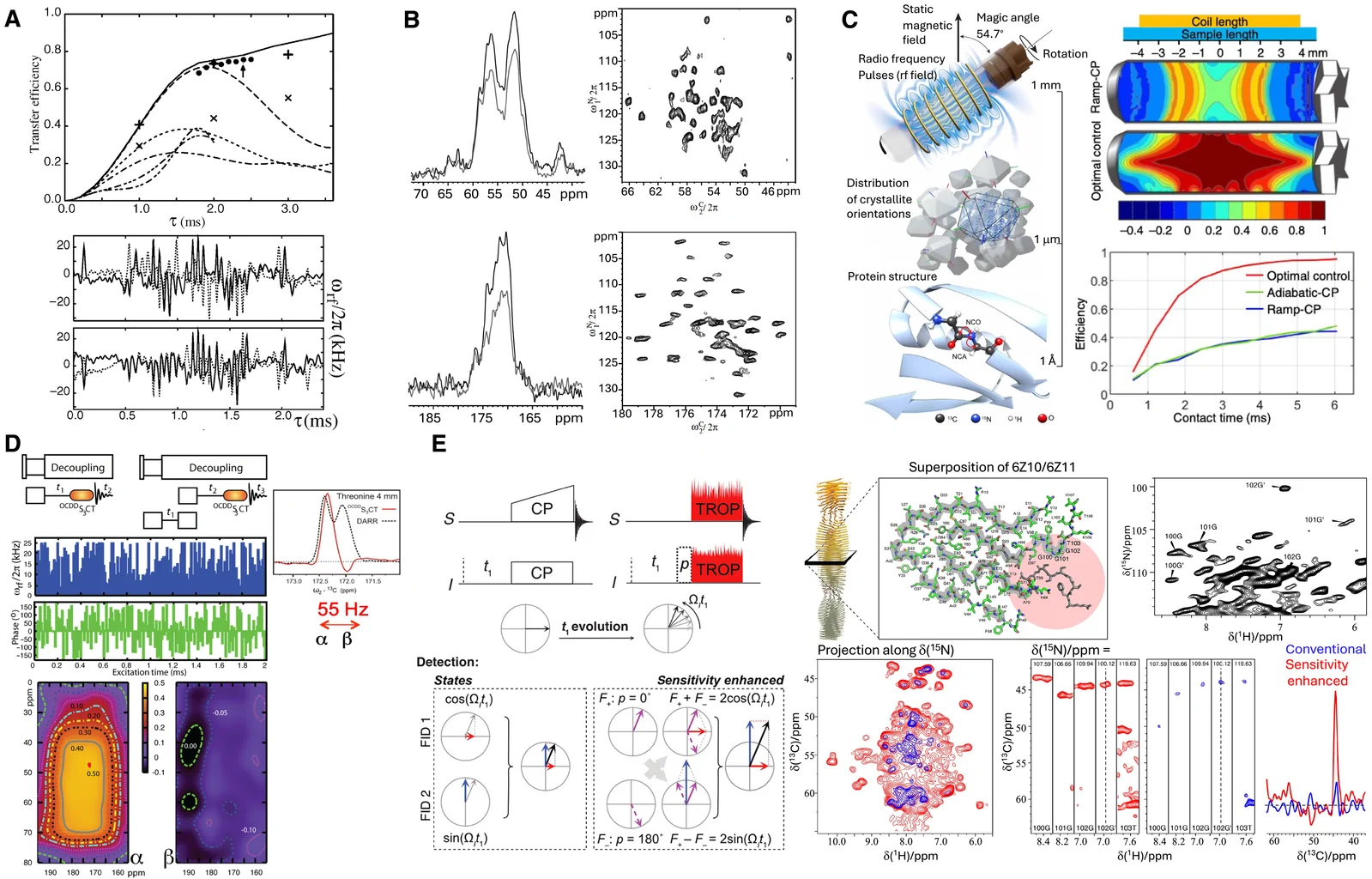
Examples of optimal control applications to solid-state NMR. (**A**) Increasing ^15^N to ^13^C_α_ polarization transfer by 50 to 100% (top) using an optimal control–derived ^OC^DCP pulse sequence (bottom) (*292*). Reproduced from (*292*) with permission. Copyright 2004 American Chemical Society. (**B**) 2D ^15^N-^13^C_α_ (top) and ^15^N-^13^C′ (bottom) obtained using ^OC^DCP (black) relative to DCP (gray) (*293*). Adapted from (*293*) with permission from Elsevier. (**C**) Optimal control taking into account spatially modulated RF inhomogeneity, schematically illustrated to the left, and inhomogeneity profile and gain in sensitivity to the right (*297*). Modified from Tosner *et al.*, *Science Advances* 7, eabj5913 (2021) (*297*) with permission from AAAS. (**D**) Optimal control doubling the resolution of ^13^C-^13^C correlation spectra by S_3_ excitation of the α-spin-state resonance of $J_{\text{CC}}$ doublets by ^OCDD^S_3_CT (*305*). Reproduced from (*305*) with permission, Copyright 2011 American Chemical Society. (**E**) Transverse mixing optimal control pulses (TROP) dipolar recoupling (*309*) simultaneously optimizing two state-state transfers (left), resulting in a $\sqrt{2}$ sensitivity enhancement (right). Adapted from (*309*) with permission, Copyright 2022 American Chemical Society.

Taking this complexity, yet very clear structure, into account, dipolar recoupling experiments taking into account all relevant internal Hamiltonians as well as experimental conditions such as available spinning, RF field strengths, RF inhomogeneity, etc., may favorably be designed using optimal control. The first applications of optimal control to solid-state NMR was in 2004 (*292*), using state-to-state optimal control with the GRAPE algorithm (*67*) to design cross-polarization (*52*) MAS (CP/MAS) experiments initially addressing ^15^N-^13^C dipolar recoupling in a so-called double cross-polarization (DCP) setup (*59*). The optimization maximized the state-to-state (ss) fidelity function

$$F_{\text{ss}}\left(t_{M}\right)=\frac{\langle \rho_{T}|\rho \left(t_{M}\right)\rangle }{\langle \rho_{T}|\rho_{T}\rangle }$$ (5)

with $t_{M}$ representing the mixing period, $\rho_{T}$ the target destination operator, and $\rho \left(t_{M}\right)=U\left(t_{M}\right)\rho \left(0\right)U^{†}\left(t_{M}\right)$, $U\left(t_{M}\right)=\hat{T}\text{exp}-i\int_{0}^{t_{M}}H\left(t\right)\mathrm{dt}$, the actual density operator at time $t_{M}$. As illustrated in Fig. 9A, such optimizations using comparable or even lower RF field strengths and polarization buildup time doubled the transfer efficiency due to much better compensation toward inhomogeneous RF fields being deleterious for long Hartmann-Hahn match periods with the RF field working unidirectional (*292*). Figure 9B demonstrates 30 to 50% gain in sensitivity in a typical biological solid-state NMR application in NCA and NCO experiments for a uniformly ^15^N, ^13^C-labeled sample of ubiquitin (*293*), as also demonstrated for large membrane proteins (*294*). To also address experiments with transfer between spins of the same spin species, optimal control has also been used to design homonuclear dipolar recoupling experiments (*295*), and for both hetero- and homonuclear cases, optimal control has been used in combinations with pulse scaffolds from composite pulses or symmetry-based recoupling (*131*, *296*).

The first applications of optimal control in solid-state NMR revealed that recoupling in cases of small dipole-dipole couplings benefited, to a large extent, from the ability of optimal control to cope with RF inhomogeneity, typically being in the order of 5 to 10% in common MAS probes. This situation applies to ^15^N-^13^C and ^13^C-^13^C spin pairs with couplings in the order of 1 and 2 kHz, respectively. This inspired rotor-spatial design of recoupling experiments in an elegant optimal control setup, taking into account also the time-dependent modulation of the RF inhomogeneity induced by an RF solenoid coil with a finite number of windings (*297*, *298*). As illustrated in Fig. 9C, this further improves the sensitivity in favor of optimal control.

Optimal control design strategies have also been used to improve the sensitivity of MAS solid-state NMR experiments on quadrupolar spin nuclei, having important applications not only in materials science with a large abundance of quadrupolar nuclei, including ^23^Na, ^27^Al, ^51^V, ^67^Zn, and ^87^Rb, but also in biology with ^2^H, ^14^N, ^17^O, etc. in focus. One example is the MQMAS experiment (*63*) for spin *I* = 3/2 nuclei involving excitation of triple-quantum coherence, which evolves in a time period before being transferred to detectable single-quantum coherence with subsequent indirect evolution before detection. An optimal control version of the MQMAS experiment demonstrates substantial gains in sensitivity of around 50% upon optimizing the involved transfer elements (*299*). Another example is the use of uniform or partial ^2^H spin labeling of protons in proteins to increase resolution or distinguish hydrophobic/hydrophilic domains of membrane proteins. Often, such experiments are performed using triple- or quadrupole-resonance probes with relatively low ^2^H RF power capability. For such applications, optimal control proves highly useful in the design of efficient ^2^H excitation pulses and ^2^H to ^13^C cross-polarization to provide substantially higher efficiency than CP experiments typically using much higher RF amplitudes (*300*). This particular case also represents one of the notable examples where the optimal control solution directly inspired the development of analytical-based pulse sequences as notably Rotor Echo Short Pulse IRrAdiaTION (RESPIRATION) RF pulses (*300*) and ^RESPIRATION^CP cross-polarization experiments with applications for both quadrupolar and spin *I* = 1/2 nuclei (*301*–*303*). Along the same lines, optimal control theory has recently been used to optimize experiments influenced by large anisotropic chemical shielding interactions (*304*).

With the experiments above taking origin in already existing methods, optimal control may obviously also be used to design experiments that so far have not been realized by other design methods. One example is the design of spin-state–selective (S_3_) solid-state NMR experiments which for homonuclear spin-pairs in uniformly ^13^C-labeled molecules only excites either $I_{i}^{-}I_{j}^{\alpha }$ or $I_{i}^{-}I_{j}^{\beta }$ coherences implying that only one of the two doublet lines (typical $J_{\mathrm{CC}}$ couplings of 30 to 60 Hz) appears in the spectrum, resulting in much improved spectral resolution as illustrated by ${}^{\text{OCDD}}S_{3}$CT experiments in Fig. 9D (*305*).

Spin dynamics optimization may also be controlled by changing the cost function to focus on effective Hamiltonians (*132*, *134*, *306*) to effectuate the desired state-to-state transfer. This may be done indirectly by optimizing the effective propagator of the spin dynamics using

$$F_{U}\left(t_{M}\right)=\langle U_{T}\left(t_{M}\right)|U\left(t_{M}\right)\rangle$$ (6)

with $U_{T}\left(t_{M}\right)=\hat{T}\text{exp}-i\int_{0}^{t_{M}}H_{T}\left(t\right)\mathrm{dt}$ being the target propagator at time $t_{M}$, where $H_{T}\left(t\right)$ is the Hamiltonian containing both internal and external interactions, and $U\left(t_{M}\right)$ being the corresponding propagator of the experiment at time $t_{M}$. Using this approach, it has been possible to present an alternative to the prevailing planar (i.e., zero- or double-quantum) operations typical in CP and dipolar recoupling experiments, namely, isotropic mixing pulse sequences as widely known from liquid-state NMR in terms of TOCSY experiments increasing sensitivity by a factor $\sqrt{2}$ (*307*). It was demonstrated in (*308*) that it is possible to increase the sensitivity in coherence transfer in solid-state dipolar recoupling by this factor using optimal control–based heteronuclear isotropic mixing relative to planar mixing. The sensitivity gain provided by coherence transfer by isotropic mixing relates to better exploitation of all phase components in the transfer process. As illustrated in Fig. 9E, this effect may alternatively be obtained by simultaneous, balanced state-to-state optimization of two coherence transfer pathways using TRansverse mixing Optimal control Pulses (TROP) dipolar recoupling sequences providing a welcome boost in sensitivity of biological solid-state NMR (*309*).

### EPR and DNP

The potential of optimal control experiment design in magnetic resonance is obviously not limited to direct nuclear spin applications such as liquid- and solid-state NMR and MRI. It may obviously also be applied in EPR and in the rapidly emerging hybrid between EPR and NMR in terms of DNP and in parahydrogen induced hyperpolarization. Considering electron spins, the potential of optimal control may be even higher than in NMR, considering the much larger magnitudes of electron-spin interactions (megahertz to gigahertz) and thereby much longer distance span (nanometer scale), with these markedly increasing electron-nuclear spin systems. Because of this further complexity, it may also provide us an opportunity to discuss aspects of experiment design other than those covered above and their perspectives in the rapidly evolving quantum era in many modalities, exploiting the high sensitivity and long-range capability of unpaired electron spins. Here, the development of fast arbitrary waveform generators with sub-nanosecond time resolution opened the door for the application of advanced optimal control pulses (*244*, *310*).

**Fig. 10.**
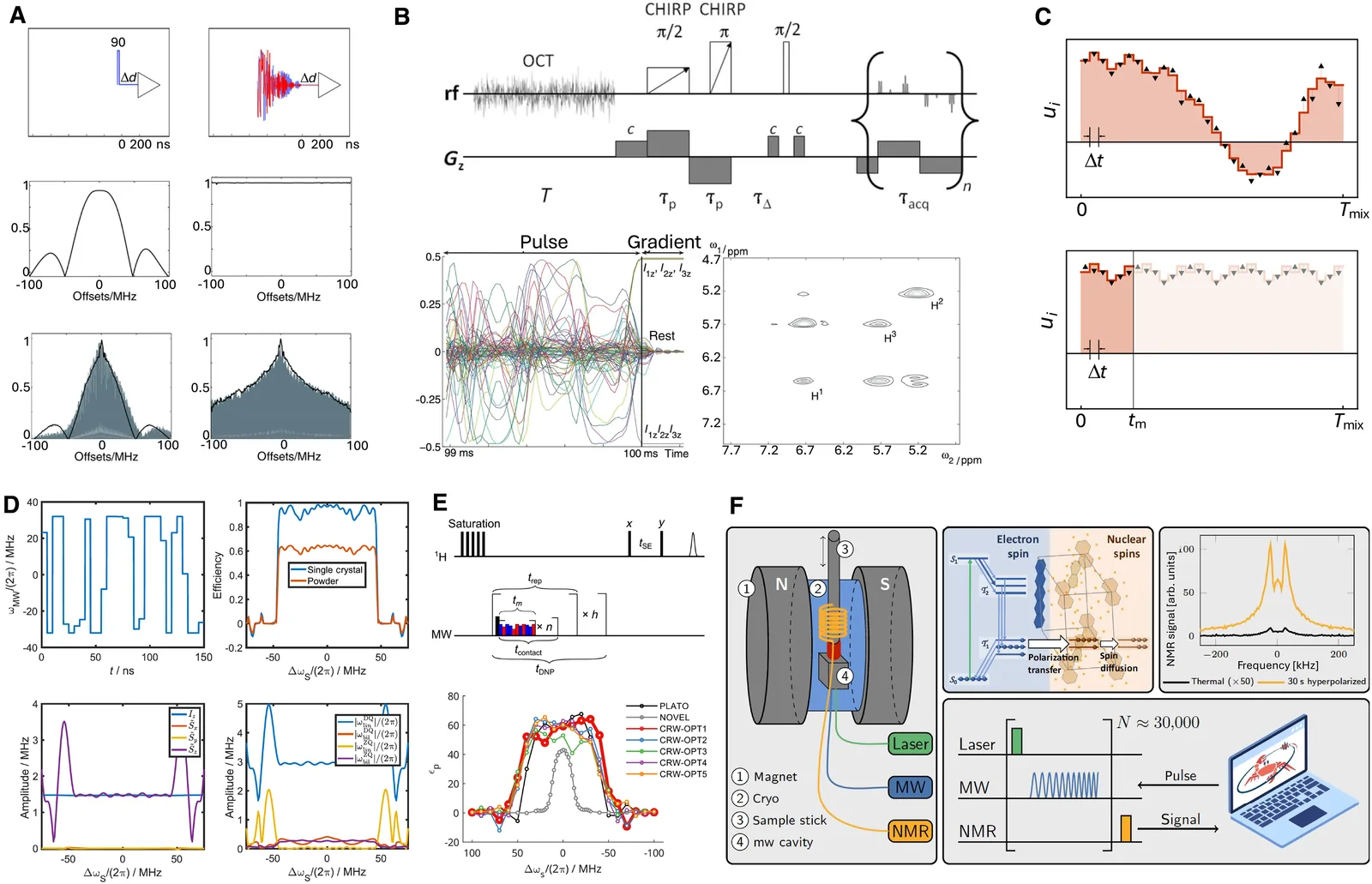
Examples of optimal control experiment design in EPR, parahydrogen-enhanced NMR, and DNP. (**A**) Pulsed excitation of EPR spectra using conventional 90° (left) and optimal control pulse excitation (*244*) with the pulse sequence in top panels, simulated excitation in the middle panels (taking into account the pulse transient response), and experimental and simulated excitation in the bottom panels (taking into account the frequency response of the used signal amplifier). Adapted from (*244*) with permission from Elsevier. (**B**) Optimal control designed conversion of parahydrogen induced two-spin longitudinal order to single-spin order by optimal control (*313*). Optimal control element (top), leading to the transfers in the bottom left panel, and experimentally demonstrated by a 2D ultrafast COSY experiment of styrene (lower right panel). Adapted from (*313*), Copyright 2012 AIP Publishing, https://doi.org/10.1063/1.3691193. (**C**) Optimization of control fields ($u_{i}$) for a full sequence (top) versus periodic elements by optimal control (*132*). Adapted from (*132*), Copyright 2025 AIP Publishing, https://doi.org/10.1063/5.0244723. (**D**) Broadband DNP cRW-OPT DNP pulse sequence (top left) with simulated (top right) offset profile, effective field (bottom left), and linear and bilinear effective Hamiltonian components (bottom right) designed by a combination of constrained random walk and nonlinear optimization (*134*). Reproduced from (*134*) with the permission of AIP Publishing. (**E**) cRW-OPT–based DNP pulse sequence (top) and experimental offset profiles in comparison with alternative experiments (*134*). Reproduced from (*134*) with the permission of AIP Publishing. (**F**) Sketch of feedback-loop setup for CRAB-based optimal control design of optically enhanced DNP experiments (*317*). Adapted from (*317*) under a CC BY 4.0 license (https://creativecommons.org/licenses/by/4.0/).

Addressing first pulsed EPR, a major challenge is the large electron spin interactions, rendering “simple” operations such as excitation of the full or even a good fraction of the EPR spectrum by MW pulses difficult with the typically available MW power. This challenge was taken up by Spindler *et al.* (*244*), designing optimal control pulse and pulse-echo experiments for EPR as demonstrated in Fig. 10A. It is evident from the figure, comparing conventional 90° pulse excitation (left column) and the optimal control pulse excitation (right column), that the optimal control approach substantially improves the bandwidth of the excitation, as here involving a sample of phenalenyl. A major challenge solved by this design was, as seen already for solid-state NMR, the presence of MW field inhomogeneity, which, for EPR, is even more pronounced and needs to be considered at the stage of spin engineering. In addition to this come substantial pulse response (or phase transient) effects (*244*, *311*). The combination of such effects, as addressed in this study, represents a great challenge for optimal control. It is envisaged that many EPR experiments may benefit from similar combinations of fast pulsed instrumentation and the design of advanced efficient pulse sequences. We note, along this line, that it has recently been demonstrated that the sensitivity of ENDOR experiments may be markedly enhanced using chirp-based adiabatic pulses (*312*).

In passing, before continuing with DNP experiments, we should also note that optimal control may also be used for the design of pulse sequences for parahydrogen-based hyperpolarization. Here, a pertinent challenge could be to convert parahydrogen-induced longitudinal two-spin order to evenly distributed single-spin polarization as addressed by Bretschneider *et al.* (*313*). As demonstrated in Fig. 10B, this may nicely be engineered by optimal control in a two-spin longitudinal spin-order $2I_{1z}I_{2z}$ to three-spin $I_{1z}+I_{2z}+I_{3z}$ Zeeman order optimization. The optimized pulse, leading to the transfers illustrated in the bottom left panel, can be implemented in an ultrafast COSY experiment (top panel) to provide the 2D spectrum for styrene shown in the lower right panel, recorded in less than 200 ms.

DNP in solids and liquids is becoming increasingly popular as a means to increase NMR sensitivity by orders of magnitude, transferring polarization from highly polarized unpaired electron spins to nuclear spins. It may also be used as an ingredient in EPR experiments to mediate contact between electron and nuclear spins. DNP represents a rich challenge for optimal control spin engineering. DNP offers many levels of challenges, including large electron-nuclear spin systems, large electron-spin involved interactions, time-differentiated electron-nuclear and nuclear-nuclear spin evolution, and instrumental challenges in terms of MW power limitations, MW homogeneity, and pulse transients. This applies in particular to the emerging area of pulsed DNP as a potential alternative to the widespread use of continuous-wave DNP, which, for instrumental reasons, is prevailing at high field.

An optimal control challenge may, in a first approach, be defined by the electron-nuclear two-spin Hamiltonian

$$H\left(t\right)=\omega_{0I}I_{z}+\Delta \omega_{S}S_{z}+{\mathrm{AS}}_{z}I_{z}+{\mathrm{BS}}_{z}I_{x}+H_{\text{mw}}\left(t\right)$$ (7)

with the first two terms describing the nuclear (*I*) Zeeman operator ($\omega_{0I}$ is the nuclear Larmor frequency) and the electron spin (*S*) offset $\Delta \omega_{S}=\omega_{S}-\omega_{\text{mw}}^{\text{carrier}}$. The third and fourth terms describe the secular and pseudo-secular hyperfine coupling with amplitudes *A* and *B*, respectively. The last term accounts for time-dependent MW irradiation on the electron spins. RF irradiation on the nuclear spins is ignored due to the large time- and amplitude-scale differences between electron and nuclear spin dynamics. In a normal optimal control setup, the pulsed MW irradiation may be described as $H_{\text{mw}}\left(t\right)=\omega_{S,x}^{\text{mw}}\left(t\right)S_{x}+\omega_{S,y}^{\text{mw}}\left(t\right)S_{y}$ with time-dependent *x*- and *y*-phase amplitudes. As described recently (*132*), optimal control may be invoked in different ways. It may optimize state-to-state transfer (cf. Eq. 5), propagator optimization (cf. Eq. 6), or, as elaborated further on in (*134*), optimization of a FOM function $F_{\text{FOM}}\left(t_{M}\right)$ (vide infra). In the last two cases, it may be advantageous to optimize the MW pulse sequences as a series of periodic elements rather than the typical “state-to-state”–type optimization of a full transfer sequence as sketched in Fig. 10C (*132*). This substantially reduces the number of variables, reduces effects from details of the spin system, and helps optimize effective Hamiltonians.

Before demonstrating specific optimal control designed DNP experiments, it is relevant to note that without RF irradiation on the nuclear spins, DNP relies on transfer mediated by the pseudo-secular hyperfine coupling (*B* in Eq. 7). This term is truncated by the nuclear-spin Zeeman term (first term in Eq. 7) unless it, in the nuclear Zeeman interaction frame, is “recoupled” by MW irradiation—very similar to MAS dipolar recoupling in solid-state NMR, as described above.

The cost function for FOM-based optimal control design of zero-quantum (p=ZQ) or double-quantum (p=DQ) effective Hamiltonians for DNP transfer experiments may be expressed as

$$\begin{array}{c} F_{\text{FOM}}^{p}\left(t_{M}\right)=\pm \langle \rho \left(0\right)|{\tilde{S}}_{z}\rangle \frac{{|\omega_{\text{bil}}^{p}|}^{2}}{{|\omega_{\text{bil}}^{p}|}^{2}+4{|\omega_{\text{lin}}^{p}|}^{2}}x\\ {\text{sin}}^{2}\left[\frac{t_{M}}{4}\sqrt{{|\omega_{\text{bil}}^{p}|}^{2}+4{|\omega_{\text{lin}}^{p}|}^{2}}\right] \end{array}$$ (8)

where $t_{M}={\mathrm{nt}}_{m}$ is the overall mixing time during which mw irradiation is invoked by repeating a basic pulse sequence element of duration $t_{m}$ *n* times to achieve maximum transfer. The factor $\langle \rho \left(0\right)|{\tilde{S}}_{z}\rangle$ ensures appropriate alignment of the initial density operator relative to the direction of the effective field (along ${\tilde{S}}_{z}$) of the MW pulse sequence. In the frame of the MW field (marked by a tilde), the linear (marked by “lin”) and bilinear (marked by “bil”) effective fields may be described as $|\omega_{\text{lin}}^{p}|$ = $|\omega_{\tilde{S}z}\mp \omega_{\mathrm{Iz}}|$ and $|\omega_{\text{bil}}^{p}|$ = $\sqrt{{\left(\omega_{\tilde{S}\mathrm{xIx}}\pm \omega_{\tilde{S}\mathrm{yIy}}\right)}^{2}+{\left(\omega_{\tilde{S}\mathrm{yIx}}\mp \omega_{\tilde{S}\mathrm{xIy}}\right)}^{2}}$ (with upper/lower signs corresponding to *p* = ZQ/DQ) and with the frequency components obtained from the total system Hamiltonian using EEHT (*51*) as described in (*134*).

In this formulation, optimal control (or nonlinear optimization) optimizes effective ZQ or DQ Hamiltonians for planar mixing. The optimization of two-spin planar effective Hamiltonians may be a great advantage in large electron-nuclear spin systems, as the effective Hamiltonian, through its bilinear character, influences all electron-nucleus spin pairs identically without needing (or capturing) the full details of the large electron-nuclear spin system. Figure 10D provides a broadband DNP pulse sequence (±x phase MW pulses), developed by optimizing the FOM function in Eq. 8 using combinations of constrained Random Walk (*134*), to provide good starting guesses, and nonlinear optimization (optimal control), along with simulated off-resonance profiles in the upper row. Below are given the amplitudes of the linear Hamiltonian components (left) and linear and bilinear Hamiltonian effective fields (right). It is evident that (i) the pulse sequence stabilizes the effective Hamiltonian over a bandwidth of 100 MHz on the electron spins and (ii) it provides the same bandwidth in numerical simulations of $S_{x}$ to $I_{z}$ transfer in a setting with a 1-MHz pseudo-secular hyperfine coupling. Figure 10E, from the same study, shows the overall pulse sequence (top) along with the offset profile obtained experimentally for OX063 trityl using the optimized cRW-OPT pulse sequences in comparison with earlier methods. We note that the cRW-OPT pulse sequences are much more broadband than the commonly used NOVEL experiment (*314*) and also more broadband than the recent PLATO experiment (*315*). In striving for the highest possible offset compensation, it is important to consider all elements of the pulse sequence. The cRW-OPT pulse sequences were designed as traditional transverse spin-lock-type cross-polarization, involving an initial 90° excitation pulse. This initial pulse may limit the broadband capability. Optimal control of periodic pulse elements has later been used to design longitudinal pulsed DNP experiments, eliminating the need for the initial excitation pulse (*133*). These studies reveal the potential of pulsed DNP as an alternative to continuous-wave technology, gradually opening new pulse sequence elements as seen in NMR spectroscopy. One recent example is the so-called DNP echoes (*316*).

Optimal control experiment design may also be used in experimental feedback-loop systems to design optimal pulse sequences as recently described by Marshall *et al.* (*317*). Such a setup requires the use of optimal control methods, such as CRAB, that only evaluate the overall efficiency rather than sampling the full density operator for optimal control processing. This represents a highly interesting approach for experiment design in cases of high instrumental sensitivity, as here ensured by optical excited DNP in a setup as shown in Fig. 10F. Extending these perspectives further, it has recently been demonstrated that the combination of artificial learning protocols and feedback-loop control can be used to design broadband DNP pulse sequences in situ directly on the DNP instrument (*318*).

Last, we should address the spin engineering–wise highly important aspect that the broadband nonlinear optimization/optimal control sequence presented in Fig. 10 (D and E) was designed by optimization of a single crystal rather than a powder, with the consequence of much faster optimizations. The only difference is a scaling factor. The validity of this approach is illustrated numerically in the top right panel in Fig. 10D and proved theoretically for FOM optimization in (*319*). In addition to this comes the important aspect that single-crystal optimized DNP sequences can be translated directly to broadband MAS dipolar recoupling applications (*319*) and even readily adapted to applications at different spinning frequencies (*320*). This has the transformative consequence that MAS dipolar recoupling sequences may be optimized on single static crystals and without the need to be optimized to different MAS frequencies, overall providing an enormous reduction in optimization time, as powder as well as spinning do not need to be involved in the optimization.

For liquid-state DNP (*321*), numerical and analytical optimal-control studies were performed on idealized model systems. These included simple two-spin systems, consisting of an electron spin coupled to a nuclear spin (*322*, *323*), and different three-spin systems, consisting of an electron spin and two nuclear spins (*324*), to explore the physical limits of nuclear polarization enhancement in liquid-state DNP.

### Quantum technology approaches

Quantum technologies such as quantum sensing and quantum computing rely on systems whose evolution follows the Schrödinger equation (*68*–*70*). The required dynamics are generated by external electromagnetic fields. To obtain reliable results, evolution must follow the ideal path with deviations that are substantially smaller than the precision with which electromagnetic control operations can be generated (*325*, *326*). It is therefore essential to design the control operations in such a way that perturbations are systematically canceled over the duration of the gate operation. The two main sources of errors are (i) experimental uncertainties such as errors in the amplitudes of control fields, and (ii) noise from uncontrollable interactions with the environment. The techniques summarized in this review article are primarily aimed at reducing the effect of experimental uncertainties, but they are also useful for suppressing some types of environmental noise. Depending on the requirements, they are often combined with other techniques, such as refocusing (echoes) or by encoding information in parts of the Hilbert space that are less affected by noise (*327*).

**Fig. 11.**
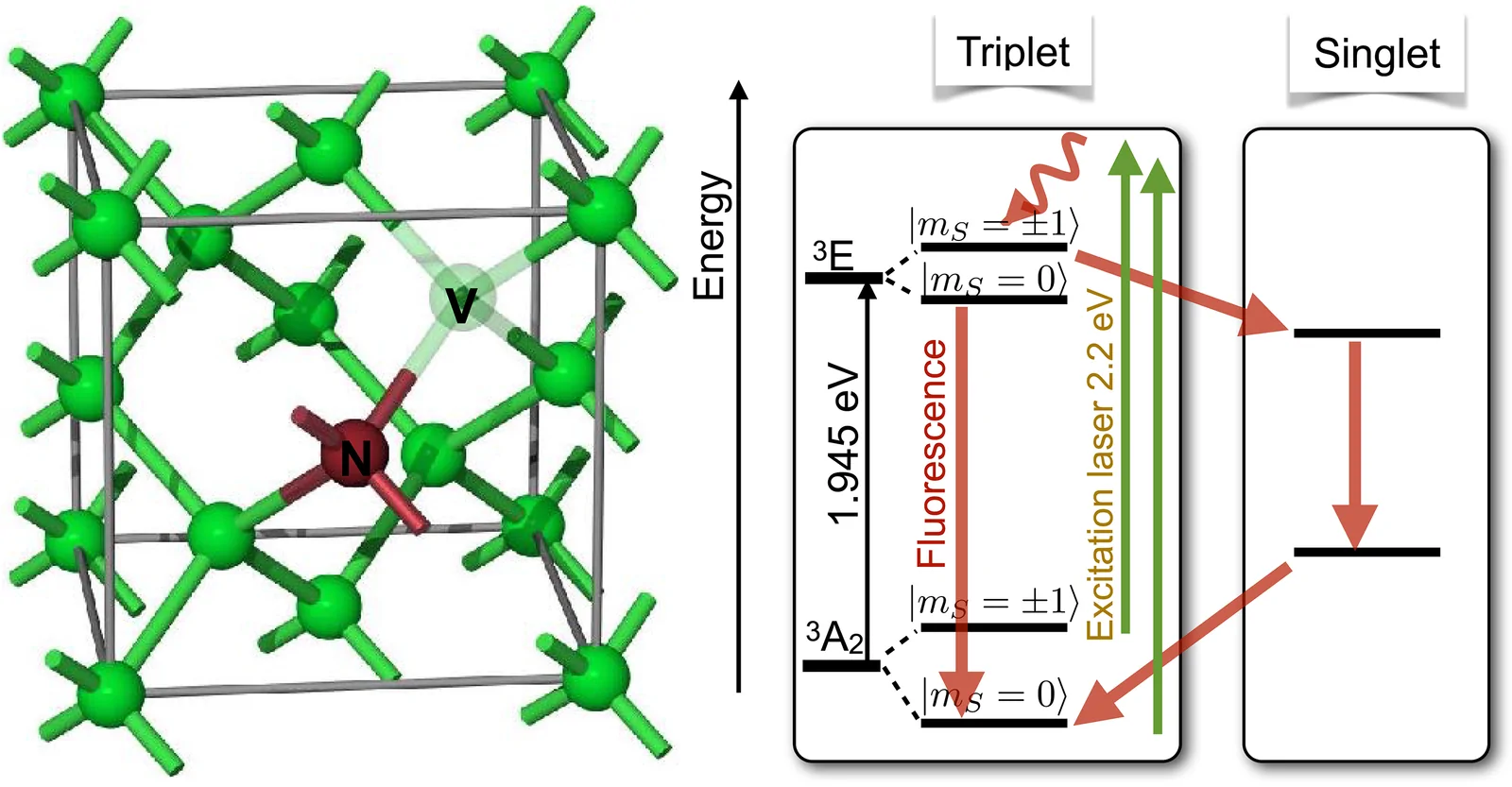
Geometric structure and energy-level scheme of a nitrogen-vacancy (NV) center in diamond. Left: NV center in a diamond lattice structure; right: relevant energy-level spectrum and transitions.

One of the most successful model systems for quantum technologies is the NV center in diamond (*306*, *328*–*330*). Figure 11 shows the geometric structure of the NV center, together with the relevant energy-level scheme. Irradiation of this center with light initializes it into a state in which most of the population is in the $m_{S}=0$ state of the electronic ground state. Because the fluorescence rate is higher when the center is initially in the $m_{S}=0$ state than in the $m_{S}=\pm 1$ state, counting fluorescence photons can also be used to read out the spin state of the center. Initialization and readout are essential steps for all quantum technologies. They are typically combined with spin control through electromagnetic fields. In addition to the electron spin, the system also includes nuclear spins: The nitrogen atom is typically a ^14^N (I=1) spin, but in many applications, isotopically enriched ^15^N (I=1/2) is implanted for specific applications. The combined spin system is controlled by resonant ac magnetic fields with frequencies near the resonances of the electron- and nuclear spin transitions.

**Fig. 12.**
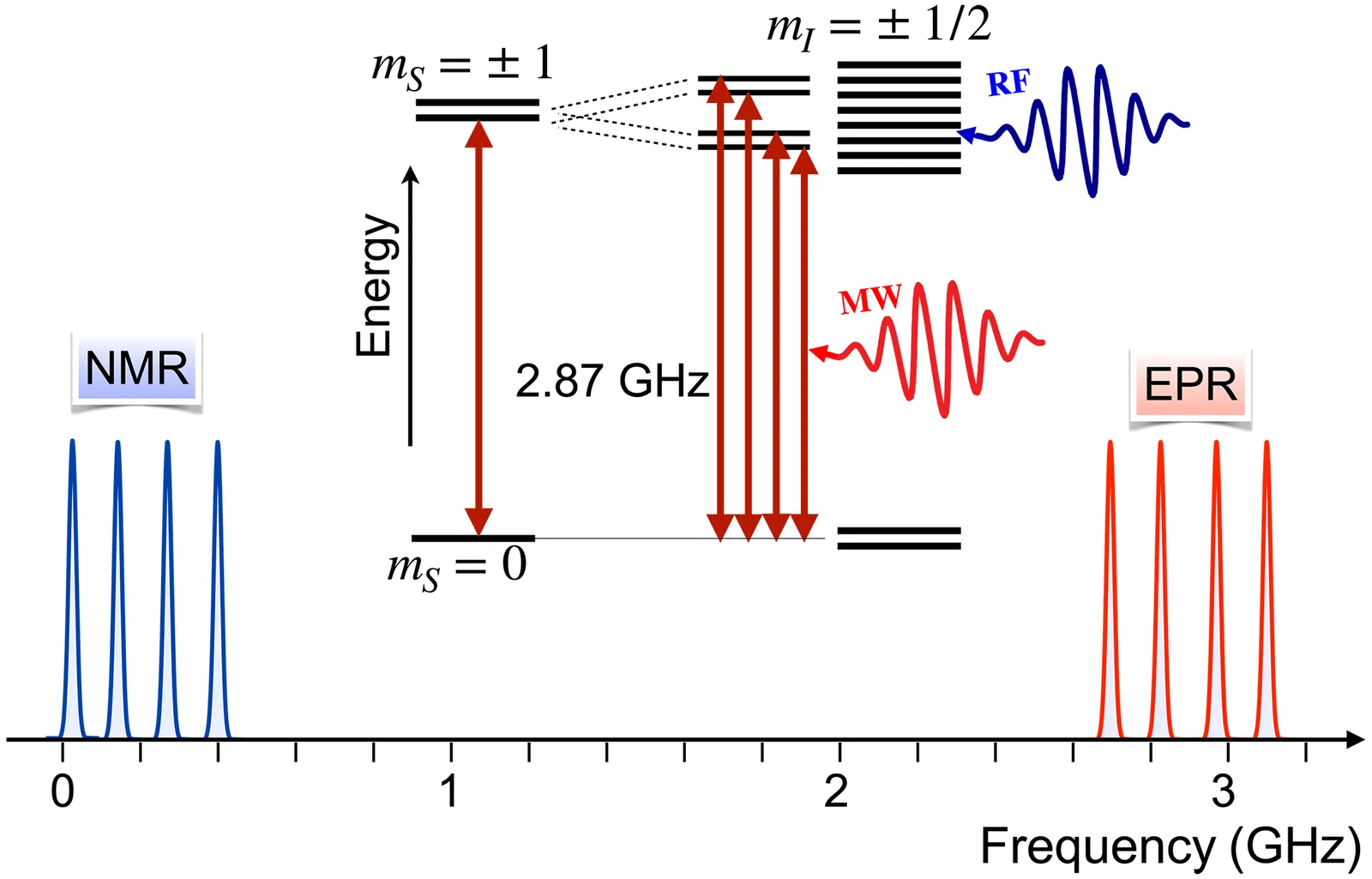
NMR and EPR transitions of an NV center in diamond. The figure shows energy levels and transition frequencies of an NV spin system, which is taken here to consist of an S=1 electron spin and an I=1/2 nuclear spin (^13^C or ^15^N).

Figure 12 shows the energy levels and the relevant frequency ranges for the case of a small magnetic field, where the transition frequencies of the electron spin are dominated by the zero field splitting and the electron Zeeman interaction, and the NMR transitions by a combination of the hyperfine interaction and the nuclear Zeeman interaction. The relevant frequencies are then in the range of ≈3 GHz and ≈1 MHz.

In many experimental setups (*329*), the ac fields are applied by a simple wire, which is fully broadband but does not provide resonant enhancement. Although the Rabi frequencies for the electron spins can still reach tens of megahertz, nuclear spin Rabi frequencies are limited to ≈1 kHz. This is often insufficient for high-fidelity quantum gate operations. In addition, long, high-power, low-frequency pulses strongly affect the phase of the electron spin coherence, even if their frequency is very far from resonance (*331*).

An approach that solves both problems (low Rabi frequency for the nuclear spin and unwanted side effects for the electron spin) is to omit the RF pulses, replacing them by indirect control operations (*332*–*334*). In this case, only MW pulses are applied to the electron spin. If the pulse sequence parameters are properly adjusted, the resulting dynamics of the electron spin drives the nuclear spins toward the required target state through the anisotropic hyperfine interaction.

The earliest spin-based demonstrations of quantum information processing used liquid-state NMR experiments on the nuclear spins of large ensembles of identical molecules (*335*). The first two-qubit (*336*) and the first three-qubit (*337*) experiments were published in 1998, followed by the first five-qubit (*338*) experiments published in 2000, which used a custom-designed and synthesized isotope-labeled molecule. The increasing complexity of the spin systems and of the pulse level control sequences motivated the search for efficient ways to implement state preparation, quantum gates, and compilation tools using optimal control techniques. The problem of state preparation was studied, e.g., in NMR systems for the flexible and efficient creation of pseudo-pure states (*239*) and in the context of the one-way quantum computation model for the time-optimal preparation of cluster states (*339*). Time-optimal realizations of quantum gates were designed using analytical (*166*, *167*, *176*, *272*, *274*) and numerical (*139*, *251*, *340*–*343*) optimal control techniques. In the presence of relaxation, it was shown that in systems with subspaces of weak decoherence, relaxation-optimized quantum gates (*344*) perform substantially better than time-optimal gates, which were optimized without taking relaxation into account. Optimal control–based pulse sequences for state preparation and for quantum gates have also been specifically designed for experimental platforms or computational architectures beyond NMR, such as superconducting qubits (*123*, *251*, *345*), trapped ions (*340*), trapped atoms (*139*), crystals doped with rare-earth ions (*252*), circuit quantum electrodynamics in one and two dimensions (*346*), and spins coupled to a cavity (*347*).

Recently, the development of the feedback GRAPE algorithm (*348*) made it possible to also use efficient optimal control–based numerical optimizations for arbitrarily strong (discrete or continuous) stochastic measurements with applications in quantum sensing, quantum communication, and quantum error correction.

Optimal control tools are also highly useful in the task of compiling a high-level description of a quantum algorithm into a sequence of executable instructions for a concrete hardware device (*349*). For the first NMR quantum computing demonstrations with just a few qubits, quantum algorithms were translated into a streamlined sequence of control pulses by hand until the first automatic quantum compilers were developed (*350*). A first step in the compilation process is to break down the quantum algorithm into possible sequences of hardware-supported quantum gates. These gates can be seen as elements of an instruction set. Using a standard restricted instruction set (consisting only of single- and two-spin quantum gates) results in quantum RISC (reduced instruction set computer) compilation. However, substantially improved performance can be achieved using quantum CISC (complex instruction set computer) compilation based on more general instruction sets. These also include many-qubit gates that are preoptimized for the hardware of interest and which can be recursively used to go beyond the system size that a classical computer can optimize, as demonstrated for the quantum Fourier transform, indirect SWAP gates, and multiply controlled CNOT gates (*349*, *351*). Furthermore, the use of time complexity rather than network complexity (such as two-qubit gate counts) was suggested to identify the best assembly of CISC building blocks (*249*).

An interesting case is the experimental implementation of *z* rotations, which are required for many standard decompositions of quantum gates. In principle, composite pulses or optimal control–based pulses of finite durations could be used to experimentally implement such *z* rotations. However, the best (and shortest) control is to avoid applying physical control pulses altogether! Following this principle, in the paper introducing the first five-qubit quantum computer (2000), it was explained and experimentally demonstrated how any *z* rotation by an angle ϕ can be implemented in zero time by a simple frame change (corresponding to a phase shift of all subsequent pulses by −ϕ), resulting in a substantial reduction of physical single-qubit operations [see appendix B.2 in (*338*)]. In 2016, this concept appears to have been independently rediscovered in the context of superconducting qubits and was popularized as “virtual Z gates” (*352*).

## APPLICATIONS IN ATOMS AND MOLECULES

Atoms and molecules are hosts of both electron and nuclear spins, and their control has been discussed above. In addition, atomic or molecular spatial degrees of freedom are often treated as so-called pseudo-spins. For example, when describing the interaction of atoms with electromagnetic radiation, the atom is often modeled as a two-level system. This is justified when the spectral width of the electromagnetic field is much narrower than the atomic transition frequencies. The two states are typically electronic states, but for very narrow-band excitation, the approximation also holds for states within the fine-structure or hyperfine manifold of the same electronic state. No matter which atomic states make up the two-level system, the model is referred to as pseudo-spin because many concepts, for example, the Bloch equations or Rabi oscillations, that were originally developed for physical spins, have been adapted to atomic and molecular physics. Besides physical spins and pseudo-spins, atoms and molecules have spatial angular momenta, such as the orbital angular momentum of electrons or the rotation of a molecule in space. No matter, whether a given degree of freedom is a spin, pseudo-spin, or spatial angular momentum, by construction, the coordinates of the corresponding operator satisfy the same commutation relations. The only difference is that the eigenvalues for spatial angular momenta cannot be half-integer and the Hilbert space dimension is large (strictly speaking even infinite). In yet another form of “pseudo-spin,” spatial degrees of freedom can be mapped onto an angular momentum degree of freedom. Popular examples are the Jordan-Schwinger representation of two coupled quantum harmonic oscillators or modeling a Bose-Einstein condensate (BEC) in a double-well potential as a (nonlinear) angular momentum.

These angular momentum degrees of freedom have become a popular resource for quantum technologies. The resulting need for precise control of atoms and molecules, including both their spin and spatial degrees of freedom, has triggered the development of advanced control protocols. Often, these have been derived with optimal control techniques. This is the focus of the present section where we discuss ways to control degrees of freedom that correspond to quantum mechanical angular momentum, from atomic pseudo-spins to molecular rotations and their coupling with “true” physical spins, i.e., electron or nuclear spins.

### Control of atomic pseudo-spins

**Fig. 13.**
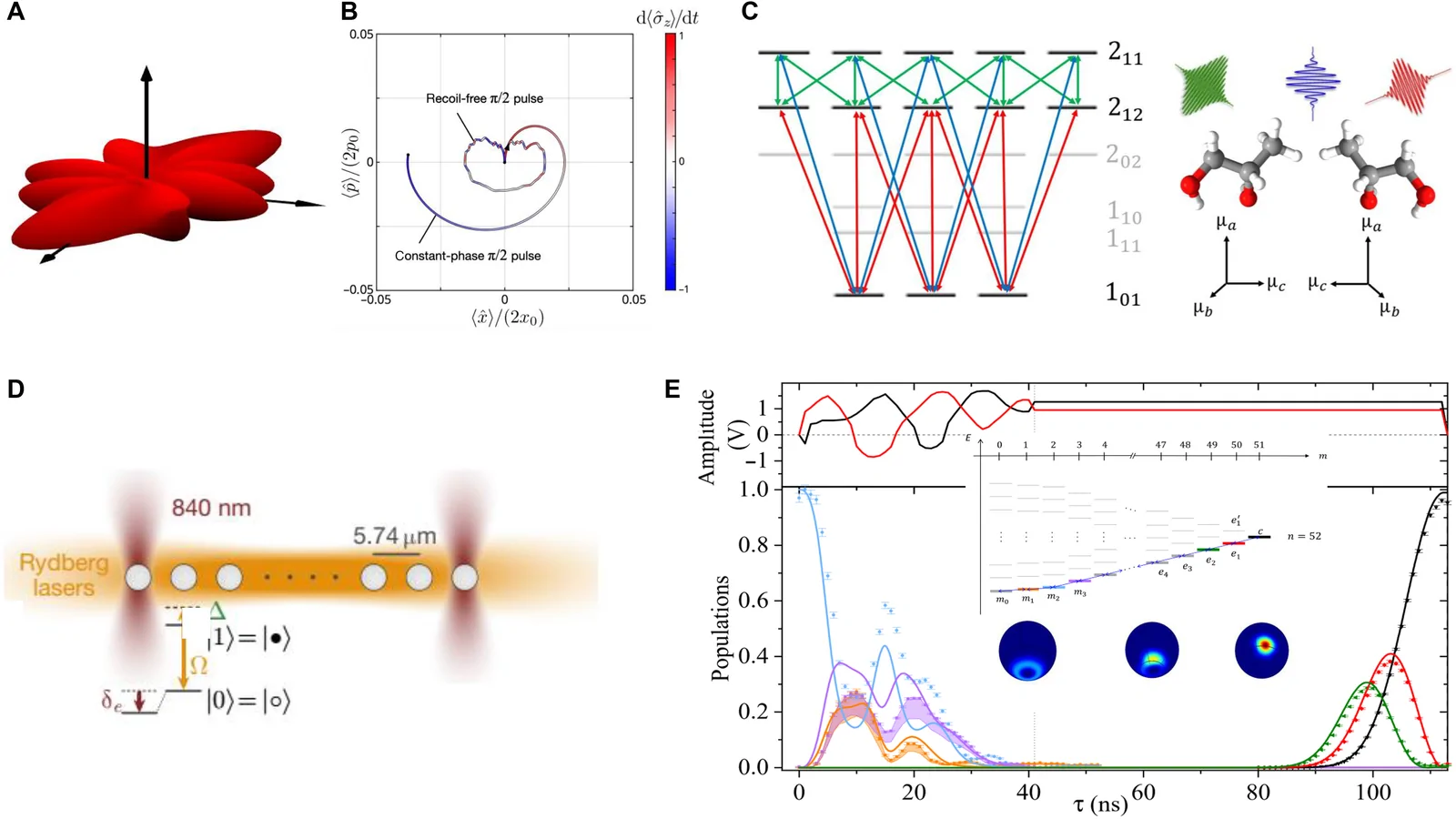
Examples of control problems in pseudo-spin systems. (**A**) Molecular rotation: Planar alignment (angular distribution) in the CO molecule at T=30 K using the control strategy proposed in (*381*) with laser intensities reduced by half. (**B**) Phase space trajectories of a trapped cold atom’s motional state while applying a laser pulse creating a $\sqrt{X}$ gate for the pseudo-spin consisting of two electronic levels, with constant amplitude and phase (open trajectory) and optimized phase modulation of a recoil-free laser pulse (closed trajectory). Adapted from (*343*) with permission. (**C**) Molecular rotation: Controlling chiral molecules to perform enantio-selective state transfer (*153*). Adapted from (*153*) under a CC BY 4.0 license (https://creativecommons.org/licenses/by/4.0/). (**D**) Atomic pseudo-spins: Experimental optimal generation of highly entangled states in Rydberg atoms (*357*). Reproduced from Omran *et al.*, *Science*, 10.1126/science.aax9743 (2019), AAAS. (**E**) Atomic pseudo-spins: Experimental implementation of optimal pulses to navigate the Stark manifold of a Rydberg atom (*353*).

The analogy between physical spins and atomic pseudo-spins works particularly well when the latter are hosted in highly excited electronic states, also referred to as Rydberg states. These states are long lived and can be very accurately modeled, allowing for an almost seamless integration of theory and experiment, much like in NMR. The key difference to physical spins is the coupling of these pseudo-spins to the electromagnetic field, which involves (in leading order) the electric, rather than the magnetic dipole moment. A control problem where numerical optimal control proved indispensable is the fast and accurate preparation of so-called circular Rydberg states (*353*, *354*). These are the states for which the projection quantum number takes its maximal value. The probability density of the electron in this state is reminiscent of a circular donut, hence the name. Experimentally, these states are prepared by laser excitation involving the absorption of two optical photons, followed by the absorption of *n* − 1 RF photons where *n* is the principal quantum number defining the Rydberg manifold. The dynamics under the RF drive were optimized with Krotov’s method (*354*). Experimental implementation of these pulse shapes allowed for improving the fidelity from about 80% obtained with a generalized π pulse to about 98% (*353*), limited by the detection accuracy. The shaped pulses and the dynamics they induce are shown in Fig. 13E, with the inset displaying the Husimi-*Q* function at the initial, intermediate, and final times, revealing that the first 40 ns are used to transfer the initial state [with projection quantum number m=2 (light blue)] into a spin-coherent state that then is rotated onto the north pole, corresponding to the desired circular state (black). In this optimization, a single Rydberg atom was modeled as a large spin (with *J* ∼ 50 for rubidium atoms). When the Rydberg atoms are subject only to laser control, they can also be modeled as two-, three-, or four-level systems, depending on the exact excitation scheme. Optimal control has been used to prepare arrays of such Rydberg atoms into highly entangled states using the dressed CRAB method (*355*), both theoretically (*356*) and in experiment (*357*), the latter producing maximally entangled states, so-called GHZ states, of about 20 atoms (see Fig. 13D).

In addition to Rydberg state preparation, optimal control has also been used to improve elementary quantum gates with Rydberg atoms (*358*–*361*). For example, optimal control showed that in the so-called Rydberg blockade regime, where the joint excitation of both atoms into the Rydberg level is strongly suppressed, the gate fidelity is limited by the movement of the atoms in the trap (*361*). Also, outside the Rydberg blockade regime, the minimal gate duration is set by the requirement to preserve the motional state of the atom in the trap (*358*). However, the duration of single-qubit gates can be reduced by orders of magnitude if the cost function for the laser pulse includes not only the effect on the pseudo-spin (formed by two electronic states) but also the effect on the motional state of the atom itself. The goal is to find a laser pulse that creates a desired quantum gate for the qubit, but which at the same time has the property that—although there is a recoil-based momentum transfer from the laser pulse to the atom—the atom returns to its initial position and is again at rest after the laser pulse. Hence, it is a coupled qubit-harmonic oscillator problem, and Fig. 13B shows a comparison of the phase-space trajectories of the atom during a laser pulse without recoil mitigation and during an optimized robust recoil-free and motion-insensitive laser pulse (*343*). When carrying out the optimization for an ensemble of system copies reflecting the uncertainty in the parameters of the Hamiltonian, gates with inherent robustness can be obtained, in contrast to gates relying on adiabatic techniques, which are robust for population transfer but not with respect to phase fluctuations (*360*). A similar conclusion on robustness for numerically optimized as compared to pulse shapes for adiabatic evolution was drawn when maximizing spin squeezing in a collection of *N* two-level atoms (*362*). Optimal control has also been used to derive shaped (classical) light fields that optimize storing a photon, i.e., an excitation of a quantized field mode, in an atomic pseudo-spin (*363*, *364*), or that drive the atomic pseudo-spin to control the state of the quantized field mode (*365*). Methods for estimating and compensating nonlinear distortions applied to Rydberg atoms have been proposed in (*366*).

Other applications have been carried out in a BEC. For example, two-level approximations are used in atom interferometry to design pulses that are robust against experimental uncertainties (*367*–*370*). Optimal control techniques have been used to manipulate a two-component BEC (bosonic Josephson junction), in which the atom operators are mapped to angular momentum coordinates (*371*–*374*). The optimal generation of squeezed (*375*) and anticoherent (*376*) spin states has also been shown.

### Control of angular momenta in molecules

The rotation of objects in 3D space is an instance of quantum angular momentum very much like spin, except that only integer quantum numbers are allowed (*377*). When the coupling between rotations in space and vibrations within the molecule can be neglected, molecules can be described as rigid linear, symmetric, or asymmetric top rotors, depending on the geometry of their nuclear scaffold. Both MW and laser pulses can be used to control molecular rotational dynamics in the gas phase (*378*, *379*). Electric fields in the RF, MW, and terahertz spectral range drive rotational transitions resonantly, provided the molecules are polar. Otherwise, the molecular electric-dipole polarizability or hyperpolarizability provides a handle for control that requires neither a “permanent” dipole moment nor resonant frequencies. For this latter type of control, typically optical pulses are used because they can be made intense enough to drive off-resonant transitions. Resonant excitation, by definition, operates on timescales that resolve the rotational eigenstates. It is at the core of rotational spectroscopy and allows the preparation of quantum superposition states with well-defined rotational energy and angular momentum (*379*). Conversely, nonresonant excitation is best suited to prepare a well-defined orientation, i.e., a superposition of many rotational eigenstates (*378*, *379*).

When controlling molecular rotation with off-resonant electric fields, a natural goal is to align or orient the molecular axes along the laboratory frame axes, leading to molecular alignment and orientation, respectively (*378*, *379*). Rather than targeting a final rotational probability distribution, an alternative approach is to manipulate the molecule’s angular momentum. In linear molecules, angular momentum is perpendicular to the molecular axis. In this case, alignment of the angular momentum along a fixed axis leads to planar alignment, i.e., confinement of the molecule to a given plane (*380*). Orientation also induces unidirectional rotation in this plane (*339*). Such dynamical processes have been observed in experiments conducted in the terahertz or optical regime (*381*–*385*), as illustrated in Fig. 13A for the CO molecule [see (*381*) for details]. Because of experimental constraints, such studies implemented only suboptimal solutions, consisting of a series of ultrashort laser pulses with specific amplitudes and polarizations. Additional descriptions and references can be found in (*379*).

In resonant excitation, the analogy between spin and molecular rotation is more apparent, as these leverage sequences of π and π/2 pulses for population transfer and the creation of coherences, but unlike in spin systems, the role of symmetry is more easily understood. Because of the isotropy of space, the rotor Hamiltonian is invariant under global rotations. As a result, all energy eigenstates form rotation-group multiplets, with multiplicity 2J+1, where *J* is the rotational quantum number, reflecting the orientational degeneracy. This degeneracy can be broken by external electric or magnetic fields. However, the magnetic moment of molecules is often very small, and it is thus important that the orientational degeneracy can also be broken with electric fields alone, as shown by proving complete controllability of linear (*152*) and asymmetric top (*151*, *153*) rotors, assuming sufficiently many electric field polarization directions are combined. In addition to the rotational symmetry, symmetric top molecules also have an axial symmetry. This symmetry cannot be broken by an external field, at least not in the rigid rotor approximation. Complete controllability guarantees the existence of control solutions even for quite complex control problems, such as the energetic separation of populations in degenerate states. This can be used as a precursor to purify a rotational mixture or also a racemic mixture of chiral molecules (*153*) (see Fig. 13C). Understanding the conditions for enantiomer-selective control allows for constructing pulse sequences that create a chiral vibrational wave packet, with net chiral signals, in ensembles of initially randomly oriented achiral molecules (*386*). Similarly, complete controllability guarantees that any rotational state can be addressed such that MW excitation in combination with a suitable dissipation mechanism allows for cooling the rotational degrees of freedom of polyatomic molecules (*387*). Preparing molecules in a selected pure rotational state is a prerequisite for their use in quantum technologies, from quantum simulation (*379*) and quantum error correction (*388*) to quantum sensing (*389*).

## OUTLOOK

As this review shows, optimal control theory is now a versatile and efficient tool for manipulating spin dynamics in a variety of platforms, ranging from fundamental research to industrial and clinical applications. Despite remarkable successes over the last 20 years, a current limitation of such techniques is that transferring them to new classes of control problems is often not straightforward. Optimal control cannot be used as a black-box approach and requires precise knowledge of the model system and accurate calibration of the Hamiltonian parameters, as well as information on the experimental constraints and limitations. To further develop the use of this approach, the optimal control community will need to make a specific effort in this direction in the coming years.

The most computationally demanding part of gradient-based algorithms, namely, computing the fitness function and its gradient for a control input, can be accomplished by the process of evolution and measurement on a quantum computer (*390*–*392*). This can be combined with a quantum sensing approach to assess the quality of the control in a closed-loop system for finding the optimal control sequence (*317*, *393*). This includes the possibility of optimizing the sensitivity of a quantum measurement protocol (*394*, *395*).

An exciting future perspective is to integrate optimal control with machine learning (quantum or classical). Both are naturally framed around a cost function that quantifies how well a target (state preparation, gate fidelity, spectroscopy objective, etc.) is reached, but they bring complementary strengths and weaknesses to that control problem. Optimal control methods exploit model knowledge and cost function gradients to converge rapidly to high-fidelity solutions, yet they rely on an accurate dynamical model and can degrade if the model or noise is wrong; machine learning methods, by contrast, are powerful at model reduction and learning effective low-dimensional representations from data, but they are challenged by the scarcity and expense of quantum measurements and by the fact that “quantum physics is not big data.” It is worth mentioning that machine learning techniques have already been used for pulse design in a series of examples in quantum control as shown by a series of examples in recent works (*318*, *396*–*400*). The most productive path forward is therefore hybrid. For example, use machine learning to learn compact surrogate models, parameterize control pulses, infer noise models, or embed symmetries and conservation laws (reducing sample complexity), and then apply gradient-based optimal control on these learned, differentiable surrogates to obtain fast, high-quality solutions. Conversely, use optimal control to provide strong priors, simulated training data, or differentiable policies that bootstrap machine learning agents. Practical tools that will accelerate this synthesis include physics-informed neural networks, Bayesian optimization, and active learning to minimize experimental queries; uncertainty-aware machine learning, such as Gaussian processes, for safe deployment; and differentiable simulators enabling end-to-end gradient flow between learned models and control objectives. Such tightly coupled, model-aware loops promise both the data efficiency needed for realistic quantum experiments and the robustness and speed required for large-scale quantum technologies.

In the prediction of the future impact of optimal control and advanced spin engineering, it is obviously important to consider methodological aspects as addressed in this review, along with future predictable combinations with fast-evolving artificial intelligence. In addition, it will be necessary to address the projections emerging from the rapidly emerging quantum “revolution.” We are currently seeing enormous resources devoted to quantum information technology and quantum sensors, with a central key component being the ability to control spins. This has sparked enormous interest in building new advanced instrumentation and merging disciplines that, for practical reasons, historically have been separated into small, almost isolated disciplines. Consider NMR, as an example, where strong communities have been built up for biological NMR, materials NMR, relaxometry, metabolomics, low-field time domain NMR, and various aspects of MRI just to mention a few. At present, and as also demonstrated above, these disciplines are perturbed by a new, potentially game-changing focus on quantum technology, as they all build on it. As exemplified above, pulse sequences developed in one area (e.g., DNP) suddenly become important in other areas (e.g., solid-state NMR). NMR and MRI researchers suddenly care about EPR as a means to boost sensitivity and information content via DNP, leading to combined EPR and NMR instrumentation. Likewise, these disciplines now take in optical excitation and detection to further enhance sensitivity and conduct measurements approaching the single-molecule level. All of this involves electron and nuclear spins, as well as atomic pseudo-spins and excitons, as the central theme. This aspect becomes even more important when addressing coupled spin systems (electron spins allow for optical access, nuclear spins provide long coherence times) gradually becoming an established platform for quantum-enhanced sensing and, with molecular spin hosts assembled on inert 2D materials or 3D framework structures, are becoming a competitor for quantum computation; such complex spin systems need optimal control and feedback. On this basis, we foresee that quantum spin engineering, including advanced design methods such as optimal control, will have a considerable impact on the future development of analytical methods and quantum devices that we today do not even dream about.

## References

[1] I. I. Rabi, Space quantization in a gyrating magnetic field. Phys. Rev. 51, 652–654 (1937).

[2] W. Gerlach, O. Stern, Der experimentelle Nachweis der Richtungsquantelung im Magnetfeld. Z. Physik 9, 349–352 (1922).

[3] B. Blümich, N. Boulant, M. Garwood, D. Parker, N. Rogers, D. Topgaard, K. Uğurbil, Magnetic resonance imaging. Sci. Adv. 12, eaeg6766 (2026).

[4] R. R. Ernst, G. Bodenhausen, A. Wokaun, *Principles of Nuclear Magnetic Resonance in One and Two Dimensions* (Clarendon Press, 1987).

[5] M. H. Levitt, *Spin Dynamics: Basics of Nuclear Magnetic Resonance* (John Wiley & Sons, 2013).

[6] D. Goldfarb, G. Jeschke, E. J. L. McInnes, T. Prisner, O. Schiemann, S. Stoll, EPR spectroscopy 80 years after its discovery. Sci. Adv. 12, eaeg0169 (2026).

[7] R. R. Ernst, Nuclear magnetic resonance Fourier transform spectroscopy (Nobel lecture). Angew. Chem. Int. Ed. Engl. 31, 805–823 (1992). 10.1007/BF01121787 1391683

[8] K. Wüthrich, NMR studies of structure and function of biological macromolecules (Nobel lecture). Angew. Chem. Int. Ed. Engl. 42, 3340–3363 (2003). 12888958 10.1002/anie.200300595

[9] P. Mansfield, Snapshot magnetic resonance imaging (Nobel lecture). Angew. Chem. Int. Ed. Engl. 43, 5456–5464 (2004). 15384128 10.1002/anie.200460078

[10] R. Sargent, Optimal control. J. Comput. Appl. Math. 124, 361–371 (2000).

[11] L. S. Pontryagin, V. Boltianski, R. Gamkrelidze, E. Mitchtchenko, *The Mathematical Theory of Optimal Processes* (John Wiley and Sons, 1962).

[12] M. M. Lee, L. Markus, *Foundations of Optimal Control Theory* (John Wiley and Sons, 1967).

[13] D. E. Kirk, *Optimal Control Theory: An Introduction* (Courier Corporation, 2004).

[14] A. E. Bryson, *Applied Optimal Control: Optimization, Estimation and Control* (CRC Press, 1975).

[15] B. Bonnard, D. Sugny, *Optimal Control with Applications in Space and Quantum Dynamics*, AIMS on Applied Mathematics (American Institute of Mathematical Sciences, 2012), vol. 2012, pp. 1–29.

[16] J.-F. Bonnans, J. C. Gilbert, C. Lemaréchal, C. A. Sagastizábal, *Numerical Optimization: Theoretical and Practical Aspects* (Springer Science & Business Media, 2006).

[17] N. F. Ramsey, A molecular beam resonance method with separated oscillating fields. Phys. Rev. 78, 695–699 (1950).

[18] E. L. Hahn, Spin echoes. Phys. Rev. 80, 580–594 (1950).

[19] H. Y. Carr, E. M. Purcell, Effects of diffusion on free precession in nuclear magnetic resonance experiments. Phys. Rev. 94, 630–638 (1954).

[20] U. Haeberlen, J. S. Waugh, Coherent averaging effects in magnetic resonance. Phys. Rev. 175, 453–467 (1968).

[21] O. Sørensen, G. Eich, M. Levitt, G. Bodenhausen, R. Ernst, Product operator formalism for the description of NMR pulse experiments. Prog. Nucl. Magn. Reson. Spectrosc. 16, 163–192 (1984).

[22] I. Kuprov, *Spin: From Basic Symmetries to Quantum Optimal Control* (Springer Nature, 2023).

[23] D. J. Tannor, V. Kazakov, V. Orlov, “Control of photochemical branching: Novel procedures for finding optimal pulses and global upper bounds,” in *Time-Dependent Quantum Molecular Dynamics* (Springer Nature, 1992).

[24] W. S. Warren, H. Rabitz, M. Dahleh, Coherent control of quantum dynamics: The dream is alive. Science 259, 1581–1589 (1993). 17733021 10.1126/science.259.5101.1581

[25] S. A. Rice, M. Zhao, *Optical Control of Molecular Dynamics* (Wiley, 2000).

[26] M. Dantus, V. V. Lozovoy, Experimental coherent laser control of physicochemical processes. Chem. Rev. 104, 1813–1860 (2004). 15080713 10.1021/cr020668r

[27] M. H. Levitt, R. Freeman, NMR population inversion using a composite pulse. J. Magn. Reson. 33, 473–476 (1979). 10.1016/j.jmr.2011.08.016 21903438

[28] M. H. Levitt, Composite pulses. Prog. Nucl. Magn. Reson. Spectrosc. 18, 61–122 (1986).

[29] M. H. Levitt, “Composite pulses,” in *Encyclopedia of Nuclear Magnetic Resonance*, D. M. Grant, R. K. Harris, Eds. (Wiley, 1996), vol. 2, pp. 1396–1404.

[30] M. H. Levitt, Symmetry principles in spin dynamics. J. Magn. Reson. 93, 145 (1991).

[31] M. H. Levitt, Symmetry in the design of NMR multiple pulse sequences. J. Chem. Phys. 128, 052205 (2008). 18266410 10.1063/1.2831927

[32] B. Luy, K. Kobzar, T. E. Skinner, N. Khaneja, S. J. Glaser, Construction of universal rotations from point-to-point transformations. J. Magn. Reson. 176, 179–186 (2005). 16009584 10.1016/j.jmr.2005.06.002

[33] W. S. Warren, Effects of arbitrary laser or NMR pulse shapes on population inversion and coherence. J. Chem. Phys. 81, 5437–5448 (1984).

[34] W. S. Warren, “The use of shaped pulses in NMR and coherent spectroscopy,” in *Advances in Magnetic and Optical Resonance* (Academic Press, 1990).

[35] R. Freeman, Shaped pulses in high-resolution NMR. Prog. Nucl. Magn. Reson. Spectrosc. 32, 59–106 (1998).

[36] C. J. Lee, N. Murali, W. S. Warren, NMR dipole dipole refocusing with shaped pulses. J. Magn. Reson. 84, 643–647 (1989).

[37] B. Ewing, S. J. Glaser, G. P. Drobny, Development and optimization of shaped NMR pulses for the study of coupled spin systems. Chem. Phys. 147, 121–129 (1990).

[38] S. J. Glaser, G. P. Drobny, The tailored TOCSY experiment-chemical-shift selective coherence transfer. Chem. Phys. Lett. 164, 456–462 (1989).

[39] S. J. Glaser, G. P. Drobny, “Assessment and optimization of pulse sequences for homonuclear isotropic mixing,” in *Advances in Magnetic Resonance*, W. S. Warren, Ed. (Academic Press, 1990), vol. 14, pp. 35–58.

[40] M. G. Schwendinger, J. Quant, J. Schleucher, S. J. Glaser, C. Griesinger, Broad-band heteronuclear Hartmann-Hahn sequences. J. Magn. Reson. Ser. A 111, 115–120 (1994).

[41] J. Quant, T. Prasch, S. Ihringer, S. J. Glaser, Tailored correlation spectroscopy for the enhancement of fingerprint cross peaks in peptides and proteins. J. Magn. Reson. Ser. B 106, 116–121 (1995). 7850181 10.1006/jmrb.1995.1021

[42] S. J. Glaser, J. J. Quant, “Homonuclear and heteronuclear Hartmann–Hahn transfer in isotropic liquids,” in *Advances in Magnetic and Optical Resonance*, W. S. Warren, Ed. (Academic Press, 1996), vol. 19, pp. 59–252.

[43] T. Carlomagno, M. Maurer, M. Sattler, M. G. Schwendinger, S. J. Glaser, C. Griesinger, PLUSH TACSY: Homonuclear planar TACSY with two-band selective shaped pulses applied to C^α^, C′ transfer and C^β^, C^aromatic^ correlations. J. Biomol. NMR 8, 161–170 (1996). 22911140 10.1007/BF00211162

[44] T. Carlomagno, B. Luy, S. J. Glaser, “Kin” HEHAHA sequences, heteronuclear Hartmann-Hahn transfer with different bandwidths for spins I and S. J. Magn. Reson. 126, 110–119 (1997). 9252280 10.1006/jmre.1997.1157

[45] B. Luy, S. J. Glaser, Broadband heteronuclear Hartmann-Hahn sequences with short cycle times. J. Magn. Reson. 142, 369–371 (2000). 10648156 10.1006/jmre.1999.1957

[46] A. Tannús, M. Garwood, Adiabatic pulses. NMR Biomed. 10, 423–434 (1997). 9542739 10.1002/(sici)1099-1492(199712)10:8<423::aid-nbm488>3.0.co;2-x

[47] M. Garwood, L. DelaBarre, The return of the frequency sweep: Designing adiabatic pulses for contemporary NMR. J. Magn. Reson. 153, 155–177 (2001). 11740891 10.1006/jmre.2001.2340

[48] D. B. Zax, G. Goelman, S. Vega, Amplitude modulated composite pulses. J. Magn. Reson. 80, 375–382 (1988).

[49] L. Emsley, G. Bodenhausen, Gaussian pulse cascades: New analytical functions for rectangular selective inversion and in-phase excitation in NMR. Chem. Phys. Lett. 165, 469–476 (1990).

[50] M. Hohwy, N. C. Nielsen, Systematic design and evaluation of multiple-pulse experiments in nuclear magnetic resonance spectroscopy using a semi continuous Baker–Campbell–Hausdorff expansion. J. Chem. Phys. 109, 3780–3791 (1998).

[51] T. S. Untidt, N. C. Nielsen, Closed solution to the Baker-Campbell-Hausdorff problem: Exact effective Hamiltonian theory for analysis of nuclear-magnetic resonance experiments. Phys. Rev. E 65, 021108 (2002). 10.1103/PhysRevE.65.021108 11863504

[52] A. Pines, M. G. Gibby, J. S. Waugh, Proton enhanced nuclear induction spectroscopy. A method for high resolution NMR of dilute spins in solids. J. Chem. Phys. 56, 1776–1777 (1972).

[53] M. H. Levitt, D. Suter, R. R. Ernst, Spin dynamics and thermodynamics in solid-state NMR cross polarization. J. Chem. Phys. 84, 4243–4255 (1986).

[54] J. S. Waugh, L. M. Huber, U. Haeberlen, Approach to high-resolution NMR in solids. Phys. Rev. Lett. 20, 180–182 (1968).

[55] D. P. Burum, W. K. Rhim, Analysis of multiple pulse NMR in solids. III. J. Chem. Phys. 71, 944–956 (1979).

[56] M. Hohwy, N. C. Nielsen, Elimination of high order terms in multiple pulse nuclear magnetic resonance spectroscopy: Application to homonuclear decoupling in solids. J. Chem. Phys. 106, 7571–7586 (1997).

[57] E. Vinogradov, P. Madhu, S. Vega, High-resolution proton solid-state NMR spectroscopy by phase-modulated Lee–Goldburg experiment. Chem. Phys. Lett. 314, 443–450 (1999).

[58] M. H. Levitt, “Symmetry-based pulse sequences in magic-angle spinning solid-state NMR,” in *Encyclopedia of Nuclear Magnetic Resonance. Volume 9, Advances in NMR*, D. M. Grant, R. K. Harris, Eds. (Wiley, 2002), pp. 165–196.

[59] J. Schaefer, R. McKay, E. Stejskal, Double-cross polarization NMR of solids. J. Magn. Reson. 34, 443–447 (1979).

[60] D. Raleigh, M. Levitt, R. Griffin, Rotational resonance in solid state NMR. Chem. Phys. Lett. 146, 71–76 (1988).

[61] N. C. Nielsen, H. Bildso/e, H. J. Jakobsen, M. H. Levitt, Double-quantum homonuclear rotary resonance: Efficient dipolar recovery in magic-angle spinning nuclear magnetic resonance. J. Chem. Phys. 101, 1805–1812 (1994).

[62] Y. Lee, N. Kurur, M. Helmle, O. Johannessen, N. Nielsen, M. Levitt, Efficient dipolar recoupling in the NMR of rotating solids. A sevenfold symmetric radiofrequency pulse sequence. Chem. Phys. Lett. 242, 304–309 (1995).

[63] A. Medek, J. S. Harwood, L. Frydman, Multiple quantum magic-angle spinning NMR: A new method for the study of quadrupolar nuclei in solids. J. Am. Chem. Soc. 117, 12779–12787 (1995).

[64] E. R. Andrew, A. Bradbury, R. G. Eades, Nuclear magnetic resonance spectra from a crystal rotated at high speed. Nature 182, 1659 (1958).

[65] S. Conolly, D. Nishimura, A. Macovski, Optimal control solutions to the magnetic resonance selective ex-citation problem. IEEE Trans. Med. Imaging 5, 106–115 (1986). 18243994 10.1109/TMI.1986.4307754

[66] A. P. Peirce, M. A. Dahleh, H. Rabitz, Optimal control of quantum-mechanical systems: Existence, numerical approximation, and applications. Phys. Rev. A 37, 4950–4964 (1988). 10.1103/physreva.37.4950 9899641

[67] N. Khaneja, T. Reiss, C. Kehlet, T. Schulte-Herbrüggen, S. J. Glaser, Optimal control of cou pled spin dynamics: Design of NMR pulse sequences by gradient ascent algorithms. J. Magn. Reson. 172, 296–305 (2005). 15649756 10.1016/j.jmr.2004.11.004

[68] S. J. Glaser, U. Boscain, T. Calarco, C. P. Koch, W. Köckenberger, R. Kosloff, I. Kuprov, B. Luy, S. Schirmer, T. Schulte-Herbrüggen, D. Sugny, F. K. Wilhelm, Training Schrödinger’s cat: Quantum optimal control. Strategic report on current status, visions and goals for research in Europe. Eur. Phys. J. D 69, 279 (2015).

[69] C. P. Koch, U. Boscain, T. Calarco, G. Dirr, S. Filipp, S. J. Glaser, R. Kosloff, S. Montangero, T. Schulte-Herbrüggen, D. Sugny, F. K. Wilhelm, Quantum optimal control in quantum technologies. Strategic report on current status, visions and goals for research in Europe. EPJ Quantum Technol. 9, 19 (2022).

[70] A. Acin, I. Bloch, H. Buhrman, T. Calarco, C. Eichler, J. Eisert, D. Esteve, N. Gisin, S. J. Glaser, F. Jelezko, S. Kuhr, M. Lewenstein, M. F. Riedel, P. O. Schmidt, R. Thew, A. Wallraff, I. Walmsley, F. K. Wilhelm, The quantum technologies roadmap: A European community view. New J. Phys. 20, 080201 (2018).

[71] K. Kobzar, B. Luy, N. Khaneja, S. J. Glaser, Pattern pulses: Design of arbitrary excitation profiles as a function of pulse amplitude and offset. J. Magn. Reson. 173, 229–235 (2005). 15780915 10.1016/j.jmr.2004.12.005

[72] F. Schilling, L. R. Warner, N. I. Gershenzon, T. E. Skinner, M. Sattler, S. J. Glaser, Next-generation het eronuclear decoupling for high-field biomolecular NMR spectroscopy. Angew. Chem. Int. Ed. Engl. 53, 4475–4479 (2014). 24623579 10.1002/anie.201400178

[73] M. Lapert, “Développement de nouvelles techniques de contrôle optimal en dynamique quantique: de la Résonance Magnétique Nucléaire à la physique moléculaire,” thesis, Université de Bourgogne (2011).

[74] W. S. Warren, S. L. Hammes, J. L. Bates, Dynamics of radiation damping in nuclear magnetic resonance. J. Chem. Phys. 91, 5895–5904 (1989).

[75] Y. Zhang, M. Lapert, D. Sugny, M. Braun, S. Glaser, Time-optimal control of spin 1/2 particles in the presence of radiation damping and relaxation. J. Chem. Phys. 134, 054103 (2011). 21303088 10.1063/1.3543796

[76] C. A. Weidner, E. A. Reed, J. Monroe, B. Sheller, S. O’Neil, E. Maas, E. A. Jonckheere, F. C. Langbein, S. Schirmer, Robust quantum control in closed and open systems: Theory and practice. Automatica 172, 111987 (2025).

[77] J.-S. Li, N. Khaneja, Control of inhomogeneous quantum ensembles. Phys. Rev. A 73, 030302 (2006).

[78] J.-S. Li, N. Khaneja, Ensemble control of Bloch equations. IEEE Trans. Automat. Contr. 54, 528–536 (2009).

[79] L. Van Damme, Q. Ansel, S. J. Glaser, D. Sugny, Robust optimal control of two-level quantum systems. Phys. Rev. A 95, 063403 (2017).

[80] K. Kobzar, T. E. Skinner, N. Khaneja, S. J. Glaser, B. Luy, Exploring the limits of broadband excitation and inversion pulses. J. Magn. Reson. 170, 236–243 (2004). 15388086 10.1016/j.jmr.2004.06.017

[81] K. Kobzar, S. Ehni, T. E. Skinner, S. J. Glaser, B. Luy, Exploring the limits of broadband 90° and 180° universal rotation pulses. J. Magn. Reson. 225, 142–160 (2012). 23142001 10.1016/j.jmr.2012.09.013

[82] J. Berkheim, D. J. Tannor, The robustness of composite pulses elucidated by classical mechanics: Stability around the globe. Phys. Chem. Chem. Phys. 28, 764–775 (2026). 41363104 10.1039/d5cp03162a

[83] M. Lapert, E. Assémat, S. J. Glaser, D. Sugny, Understanding the global structure of two-level quantum systems with relaxation: Vector fields organized through the magic plane and the steady-state ellipsoid. Phys. Rev. A 88, 033407 (2013).

[84] M. Lapert, Y. Zhang, M. Braun, S. J. Glaser, D. Sugny, Singular extremals for the time-optimal control of dissipative spin 1/2 particles. Phys. Rev. Lett. 104, 083001 (2010). 20366927 10.1103/PhysRevLett.104.083001

[85] M. Lapert, Y. Zhang, S. J. Glaser, D. Sugny, Towards the time-optimal control of dissipative spin-1/2 particles in nuclear magnetic resonance. J. Phys. B At. Mol. Opt. Phys. 44, 154014 (2011).

[86] M. Lapert, E. Assémat, Y. Zhang, S. J. Glaser, D. Sugny, Time-optimal control of spin-1/2 particles with dissipative and generalized radiation-damping effects. Phys. Rev. A 87, 043417 (2013).

[87] N. Khaneja, T. Reiss, B. Luy, S. J. Glaser, Optimal control of spin dynamics in the presence of relaxation. J. Magn. Reson. 162, 311–319 (2003). 12810014 10.1016/s1090-7807(03)00003-x

[88] N. Khaneja, B. Luy, S. J. Glaser, Boundary of quantum evolution under decoherence. Proc. Natl. Acad. Sci. U.S.A. 100, 13162–13166 (2003). 14595025 10.1073/pnas.2134111100 PMC263734

[89] N. Khaneja, J. S. Li, C. Kehlet, B. Luy, S. J. Glaser, Broadband relaxation-optimized polarization transfer in magnetic resonance. Proc. Natl. Acad. Sci. U.S.A. 101, 14742–14747 (2004). 15466716 10.1073/pnas.0404820101 PMC522028

[90] D. Huber, S. J. Glaser, BEADS: A canonical visualization of quantum states for applications in quantum information processing. New J. Phys. 27, 094509 (2025).

[91] B. Koczor, F. vom Ende, M. de Gosson, S. J. Glaser, R. Zeier, Phase spaces, parity operators, and the Born-Jordan distribution. Ann. Henri Poincare 24, 4169–4236 (2023).

[92] B. Koczor, R. Zeier, S. J. Glaser, Continuous phase spaces and the time evolution of spins: Star products and spin-weighted spherical harmonics. J. Phys. A Math. Theor. 52, 055302 (2019).

[93] B. Koczor, R. Zeier, S. J. Glaser, Time evolution of coupled spin systems in a generalized Wigner representation. Ann. Phys. 408, 1–50 (2019).

[94] A. Garon, R. Zeier, S. J. Glaser, Visualizing operators of coupled spin systems. Phys. Rev. A 91, 042122 (2015).

[95] D. Leiner, R. Zeier, S. J. Glaser, Symmetry-adapted decomposition of tensor operators and the visualization of coupled spin systems. J. Phys. A Math. Theor. 53, 495301 (2020).

[96] D. Leiner, R. Zeier, S. J. Glaser, Wigner tomography of multispin quantum states. Phys. Rev. A 96, 063413 (2017).

[97] D. Leiner, S. J. Glaser, Wigner process tomography: Visualization of spin propagators and their spinor properties. Phys. Rev. A 98, 012112 (2018).

[98] A. Devra, N. J. Glaser, D. Huber, S. J. Glaser, Wigner state and process tomography on near term quantum devices. Quantum Inf. Process. 23, 359 (2024).

[99] A. Devra, L. V. Damme, F. vom Ende, E. Malvetti, S. J. Glaser, Theory and experimental demonstration of Wigner tomography of unknown unitary quantum gates. Quantum Inf. Process. 25, 268 (2026).

[100] M. Tesch, N. J. Glaser, S. J. Glaser, *SpinDrops (Version 2.0.x) [Software application]* (Technical University of Munich, 2019); https://spindrops.org/.

[101] D. Huber, M. Tesch, N. J. Glaser, and S. J. Glaser, *QuBeads (Version 1.0.1-beta1) [Software application]* (Technical University of Munich, 2024); https://github.com/denhub97/qubeads.

[102] I. Kuprov, Spin system trajectory analysis under optimal control pulses. J. Magn. Reson. 233, 107–112 (2013). 23541031 10.1016/j.jmr.2013.02.012

[103] J. von Neumann, Wahrscheinlichkeitstheoretischer Aufbau der Quantenmechanik. Nachrichten von der Gesellschaft der Wissenschaften zu Göttingen, Mathematisch-Physikalische Klasse 1927, 245–272 (1927).

[104] S. S. Köcher, T. Heydenreich, S. J. Glaser, Visualization and analysis of modulated pulses in magnetic resonance by joint time-frequency representations. J. Magn. Reson. 249, 63–71 (2014). 25462948 10.1016/j.jmr.2014.10.004

[105] D. D’Alessandro, *Introduction to Quantum Control and Dynamics* (Chapman and Hall/CRC, ed. 2, 2021).

[106] O. W. Sørensen, Polarization transfer experiments in high-resolution NMR spectroscopy. Prog. Nucl. Magn. Reson. Spectrosc. 21, 503–569 (1989).

[107] N. C. Nielsen, O. W. Sørensen, Accessible states in Liouville space. A two-dimensional extension of the universal bound on spin dynamics applied to polarization transfer in I*_N_*S*_M_* spin-1/2 systems. J. Magn. Reson. 99, 449–465 (1992).

[108] J. Stoustrup, O. Schedletzky, S. J. Glaser, C. Griesinger, N. C. Nielsen, O. W. Sørensen, Generalized bound on quantum dynamics: Efficiency of unitary transformations between non-Hermitian states. Phys. Rev. Lett. 74, 2921–2924 (1995). 10058058 10.1103/PhysRevLett.74.2921

[109] S. J. Glaser, T. Schulte-Herbrüggen, M. Sieveking, O. Schedletzky, N. C. Nielsen, O. W. Sorensen, C. Griesinger, Unitary control in quantum ensembles: Maximizing signal intensity in coherent spectroscopy. Science 280, 421–444 (1998). 9545217 10.1126/science.280.5362.421

[110] T. S. Untidt, S. J. Glaser, C. Griesinger, N. C. Nielsen, Unitary bounds and controllability of quantum evolution in NMR spectroscopy. Mol. Phys. 96, 1739–1744 (1999).

[111] T. S. Untidt, N. C. Nielsen, Analytical unitary bounds on quantum dynamics: Design of optimum NMR experiments in two-spin-12 systems. J. Chem. Phys. 113, 8464–8471 (2000).

[112] N. Khaneja, T. Reiss, C. Kehlet, T. Schulte Herbrüggen, S. J. Glaser, Optimal control of coupled spin dynamics: Design of NMR pulse sequences by gradient ascent algorithms. J. Magn. Reson. 172, 296–305 (2005). 15649756 10.1016/j.jmr.2004.11.004

[113] T. E. Skinner, T. O. Reiss, B. Luy, N. Khaneja, S. J. Glaser, Tailoring the optimal control cost function to a desired output: Application to minimizing phase errors in short broadband excitation pulses. J. Magn. Reson. 172, 17–23 (2005). 15589403 10.1016/j.jmr.2004.09.011

[114] N. I. Gershenzon, T. E. Skinner, B. Brutscher, N. Khaneja, M. Nimbalkar, B. Luy, S. J. Glaser, Linear phase slope in pulse design: Application to coherence transfer. J. Magn. Reson. 192, 235–243 (2008). 18394937 10.1016/j.jmr.2008.02.021

[115] J. Assländer, S. J. Glaser, J. Hennig, Spin echoes in the regime of weak dephasing. Magn. Reson. Med. 75, 150–160 (2016). 25640051 10.1002/mrm.25579

[116] P. M. Lefebvre, E. Van Reeth, H. Ratiney, O. Beuf, E. Brusseau, S. A. Lambert, S. J. Glaser, D. Sugny, D. Grenier, K. T. V. Koon, Active control of the spatial MRI phase distribution with optimal control theory. J. Magn. Reson. 281, 82–93 (2017). 28558274 10.1016/j.jmr.2017.05.008

[117] M. Braun, S. J. Glaser, Concurrently optimized cooperative pulses in robust quantum control: Application to broadband Ramsey-type pulse sequence elements. New J. Phys. 16, 115002 (2014).

[118] W. Kallies, S. J. Glaser, Cooperative broadband spin echoes through optimal control. J. Magn. Reson. 286, 115–137 (2018). 29241044 10.1016/j.jmr.2017.10.011

[119] S. Asami, W. Kallies, J. C. Günther, M. Stavropoulou, S. J. Glaser, M. Sattler, Ultrashort broadband cooperative pulses for multidimensional bio-molecular NMR experiments. Angew. Chem. Int. Ed. Engl. 57, 14498–14502 (2018). 29508496 10.1002/anie.201800220

[120] J. L. Neves, B. Heitmann, N. Khaneja, S. J. Glaser, Heteronuclear decoupling by optimal tracking. J. Magn. Reson. 201, 7–17 (2009). 19695913 10.1016/j.jmr.2009.07.024

[121] T. Schulte-Herbrüggen, R. Marx, A. Fahmy, L. Kauffman, S. Lomonaco, N. Khaneja, S. J. Glaser, Control aspects of quantum computing using pure and mixed states. Philos. Trans. A Math. Phys. Eng. Sci. 370, 4651–4670 (2012). 22946034 10.1098/rsta.2011.0513 PMC3441065

[122] G. Zhang, F. Schilling, S. J. Glaser, C. Hilty, Reaction monitoring using hyperpolarized NMR with scaling of heteronuclear couplings by optimal tracking. J. Magn. Reson. 272, 123–128 (2016). 27689531 10.1016/j.jmr.2016.09.006

[123] A. Devra, E. Malvetti, N. J. Glaser, A. Agarwal, I. Rungger, S. Lujan, M. Werninghaus, S. Filipp, L. Van Damme, S. J. Glaser, Dynamical decoupling using universal optimal tracking. arXiv:2606.21762 [quant-ph] (2026).

[124] M. Lapert, E. Assémat, S. J. Glaser, D. Sugny, Optimal control of the signal-to-noise ratio per unit time for a spin-1/2 particle. Phys. Rev. A 90, 023411 (2014). 10.1063/1.4906751 25637980

[125] M. Lapert, E. Assémat, S. J. Glaser, D. Sugny, Optimal control of the signal-to-noise ratio per unit time of a spin 1/2 particle: The crusher gradient and the radiation damping cases. J. Chem. Phys. 142, 044202 (2015). 25637980 10.1063/1.4906751

[126] N. Jbili, K. Hamraoui, S. J. Glaser, J. Salomon, D. Sugny, Optimal periodic control of spin systems: Application to the maximization of the signal-to-noise ratio per unit time. Phys. Rev. A 99, 053415 (2019).

[127] T. T. Nguyen, S. J. Glaser, An optimal control approach to design entire relaxation dispersion experiments. J. Magn. Reson. 282, 142–153 (2017). 28822305 10.1016/j.jmr.2017.07.010

[128] G. Rancan, T. T. Nguyen, S. J. Glaser, Gradient ascent pulse engineering for rapid exchange saturation transfer. J. Magn. Reson. 252, 1–9 (2015). 25635353 10.1016/j.jmr.2014.12.016

[129] Q. Ansel, M. Tesch, S. J. Glaser, D. Sugny, Optimizing fingerprinting experiments for parameter identification: Application to spin systems. Phys. Rev. A 96, 053419 (2017).

[130] M. Lapert, Y. Zhang, M. A. Janich, S. J. Glaser, D. Sugny, Exploring the physical limits of saturation contrast in magnetic resonance imaging. Sci. Rep. 2, 589 (2012).

[131] A. B. Nielsen, M. Bjerring, J. T. Nielsen, N. C. Nielsen, Symmetry-based dipolar recoupling by optimal control: Band-selective experiments for assignment of solid-state NMR spectra of proteins. J. Chem. Phys. 131, 025101 (2009). 19604009 10.1063/1.3157737

[132] J. P. Carvalho, D. L. Goodwin, N. Wili, A. B. Nielsen, N. C. Nielsen, Optimal control design strategies for pulsed dynamic nuclear polarization. J. Chem. Phys. 162, 054111 (2025). 39902705 10.1063/5.0244723

[133] J. Carvalho, A. B. Nielsen, D. L. Goodwin, N. Wili, N. C. Nielsen, Longitudinal pulsed dynamic nuclear polarization transfer via periodic optimal control. J. Phys. Chem. Lett. 17, 2517–2523 (2026). 41730128 10.1021/acs.jpclett.5c03708

[134] A. B. Nielsen, J. P. Carvalho, N. Wili, F. V. Jensen, D. L. Goodwin, T. S. Untidt, Z. Tošner, N. C. Nielsen, Controlling effective Hamiltonians: Broadband pulsed dynamic nuclear polarization by constrained random walk and non-linear optimization. J. Chem. Phys. 163, 144111 (2025). 41065154 10.1063/5.0287050

[135] D. Basilewitsch, H. Yuan, C. P. Koch, Optimally controlled quantum discrimination and estimation. Phys. Rev. Res. 2, 033396 (2020).

[136] S. Qin, M. Cramer, C. P. Koch, A. Serafini, Optimal control for Hamiltonian parameter estimation in non-commuting and bipartite quantum dynamics. SciPost Phys. 13, 121 (2022).

[137] Q. Ansel, E. Dionis, D. Sugny, Optimal control strategies for parameter estimation of quantum systems. SciPost Phys. 16, 013 (2024).

[138] A. Halaski, M. G. Krauss, D. Basilewitsch, C. P. Koch, Quantum optimal control of squeezing in cavity optomechanics. Phys. Rev. A 110, 013512 (2024).

[139] L. Van Damme, Z. Zhang, A. Devra, S. J. Glaser, A. Alberti, Motion-insensitive time-optimal control of optical qubits. Quantum Sci. Technol. 10, 035025 (2025).

[140] U. Boscain, M. Sigalotti, D. Sugny, Introduction to the Pontryagin maximum principle for quantum optimal control. PRX Quantum 2, 030203 (2021).

[141] D. D’Alessandro, *Introduction to Quantum Control and Dynamics*, Applied Mathematics and Nonlinear Science Series (Chapman and Hall/CRC, 2008).

[142] F. Albertini, D. D’Alessandro, Notions of control lability for bilinear multilevel quantum systems. IEEE Trans. Automat. Contr. 48, 1399–1403 (2003).

[143] S. G. Schirmer, H. Fu, A. I. Solomon, Complete controllability of quantum systems. Phys. Rev. A 63, 063410 (2001).

[144] G. Dirr, U. Helmke, I. Kurniawan, T. Schulte Herbrüggen, Lie-semigroup structures for reachability and control of open quantum systems: Viewing Markovian quantum channels as Lie semigroups and GKS Lindblad generators as Lie wedge. Rep. Math. Phys. 64, 93–121 (2009).

[145] F. Albertini, D. D’Alessandro, The Lie algebra structure and controllability of spin systems. Linear Algebra Appl. 350, 213–235 (2002).

[146] R. Zeier, T. Schulte-Herbrüggen, Symmetry principles in quantum systems theory. J. Math. Phys. 52, 113510 (2011).

[147] F. Albertini, D. D’Alessandro, Subspace controllability of multi-partite spin networks. Syst. Control Lett. 151, 104913 (2021).

[148] J. Chen, Y. Zhou, J. Bian, J. Li, X. Peng, Subspace controllability of symmetric spin networks. Phys. Rev. A 102, 032602 (2020).

[149] K. Beauchard, J.-M. Coron, P. Rouchon, Controllability issues for continuous-spectrum systems and ensemble controllability of Bloch equations. Commun. Math. Phys. 296, 525–557 (2010).

[150] N. Augier, U. Boscain, M. Sigalotti, Adiabatic ensemble control of a continuum of quantum systems. SIAM J. Control Optim. 56, 4045–4068 (2018).

[151] E. Pozzoli, M. Leibscher, M. Sigalotti, U. Boscain, C. P. Koch, Lie algebra for rotational subsystems of a driven asymmetric top. J. Phys. A Math. Theor. 55, 215301 (2022).

[152] R. S. Judson, K. K. Lehmann, H. Rabitz, W. S. Warren, Optimal design of external fields for controlling molecular motion: Application to rotation. J. Mol. Struct. 223, 425–456 (1990).

[153] M. Leibscher, E. Pozzoli, C. Pérez, M. Schnell, M. Sigalotti, U. Boscain, C. P. Koch, Full quantum control of enantiomer-selective state transfer in chiral molecules despite degeneracy. Commun. Phys. 5, 110 (2022).

[154] A. A. Agrachev, Y. L. Sachkov, *Control Theory from the Geometric Viewpoint, Encyclopaedia of Mathematical Sciences* (Springer-Verlag, 2004), vol. 87, pp. xiv+412.

[155] H. Schättler, U. Ledzewicz, *Geometric Optimal Control*, Interdisciplinary Applied Mathematics (Springer, 2012), vol. 38, pp. xx+640.

[156] Q. Ansel, E. Dionis, F. Arrouas, B. Peaudecerf, S. Guérin, D. Guéry-Odelin, D. Sugny, Introduction to theoretical and experimental aspects of quantum optimal control. J. Phys. B At. Mol. Opt. Phys. 57, 133001 (2024).

[157] U. Boscain, G. Charlot, J.-P. Gauthier, S. Guérin, H.-R. Jauslin, Optimal control in laser-induced population transfer for two- and three-level quantum systems. J. Math. Phys. 43, 2107–2132 (2002).

[158] T. E. Skinner, T. O. Reiss, B. Luy, N. Khaneja, S. J. Glaser, Application of optimal control theory to the design of broadband excitation pulses for high resolution NMR. J. Magn. Reson. 163, 8–15 (2003). 12852902 10.1016/s1090-7807(03)00153-8

[159] D. D’Alessandro, M. Dahleh, Optimal control of two-level quantum systems. IEEE Trans. Automat. Contr. 45, 866–876 (2001).

[160] U. Boscain, P. Mason, Time minimal trajectories for a spin 1/2 particle in a magnetic field. J. Math. Phys. 47, 062101 (2006).

[161] G. C. Hegerfeldt, Driving at the quantum speed limit: Optimal control of a two-level system. Phys. Rev. Lett. 111, 260501 (2013). 24483786 10.1103/PhysRevLett.111.260501

[162] D. Stefanatos, N. Khaneja, S. J. Glaser, Optimal control of coupled spins in the presence of longitudinal and transverse relaxation. Phys. Rev. A 69, 022319 (2004).

[163] D. Stefanatos, S. J. Glaser, N. Khaneja, Relaxation optimized transfer of spin order in Ising spin chains. Phys. Rev. A 72, 062320 (2005).

[164] A. D. Boozer, Time-optimal synthesis of SU(2) transfor mations for a spin-1/2 system. Phys. Rev. A 85, 012317 (2012).

[165] A. Garon, S. J. Glaser, D. Sugny, Time-optimal control of SU(2) quantum operations. Phys. Rev. A 88, 043422 (2013).

[166] N. Khaneja, S. J. Glaser, R. Brockett, Sub Riemannian geometry and time optimal control of three spin systems: Quantum gates and coherence transfer. Phys. Rev. A 65, 032301 (2002).

[167] N. Khaneja, B. Heitmann, A. Spörl, H. Yuan, T. Schulte-Herbrüggen, S. J. Glaser, Shortest paths for efficient control of indirectly coupled qubits. Phys. Rev. A 75, 012322 (2007).

[168] P. M. Poggi, G. De Chiara, S. Campbell, A. Kiely, Universally robust quantum control. Phys. Rev. Lett. 132, 193801 (2024). 38804930 10.1103/PhysRevLett.132.193801

[169] G. Dridi, M. Mejatty, S. J. Glaser, D. Sugny, Robust control of a NOT gate by composite pulses. Phys. Rev. A 101, 012321 (2020).

[170] J. Zeng, E. Barnes, Fastest pulses that implement dynamically corrected single-qubit phase gates. Phys. Rev. A 98, 012301 (2018).

[171] M. Harutyunyan, F. Holweck, D. Sugny, S. Guérin, Digital optimal robust control. Phys. Rev. Lett. 131, 200801 (2023). 38039452 10.1103/PhysRevLett.131.200801

[172] E. Barnes, F. A. Calderon-Vargas, W. Dong, B. Li, J. Zeng, F. Zhuang, Dynamically corrected gates from geometric space curves. Quantum Sci. Technol. 7, 023001 (2022).

[173] W. Dong, F. Zhuang, S. E. Economou, E. Barnes, Doubly geometric quantum control. PRX Quantum 2, 030333 (2021).

[174] M. Harutyunyan, B. Peaudecerf, D. Sugny, S. Guérin, Qubit sensing by optimal narrowband pulses: Demonstration for Rabi frequency sensing. Phys. Rev. Res. 7, 043255 (2025).

[175] L. Van Damme, Q. Ansel, S. J. Glaser, D. Sugny, Time-optimal selective pulses of two uncoupled spin-1/2 particles. Phys. Rev. A 98, 043421 (2018).

[176] N. Khaneja, R. Brockett, S. J. Glaser, Time optimal control in spin systems. Phys. Rev. A 63, 032308 (2001).

[177] N. Khaneja, S. J. Glaser, Cartan decomposition of SU(2*^n^*) and control of spin systems. Chem. Phys. 267, 11–23 (2001).

[178] D. D’Alessandro, F. Albertini, Quantum symmetries and Cartan decompositions in arbitrary dimensions. J. Phys. A Math. Theor. 40, 2439–2453 (2007).

[179] H. N. S. Earp, J. K. Pachos, A constructive algorithm for the Cartan decomposition of SU(2*^N^*). J. Math. Phys. 46, 082108 (2005).

[180] D. J. Tannor, S. A. Rice, Control of selectivity of chemical reaction via control of wave packet evolution. J. Chem. Phys. 83, 5013–5018 (1985).

[181] P. de Fouquieres, S. G. Schirmer, S. J. Glaser, I. Kuprov, Second order gradient ascent pulse engineering. J. Magn. Reson. 212, 412–417 (2011). 21885306 10.1016/j.jmr.2011.07.023

[182] I. I. Maximov, Z. Tošner, N. C. Nielsen, Optimal control design of NMR and dynamic nuclear polarization experiments using monotonically convergent algorithms. J. Chem. Phys. 128, 184505 (2008). 18532824 10.1063/1.2903458

[183] I. I. Maximov, J. Salomon, G. Turinici, N. C. Nielsen, A smoothing monotonic convergent optimal control algorithm for nuclear magnetic resonance pulse sequence design. J. Chem. Phys. 132, 084107 (2010). 20192290 10.1063/1.3328783

[184] M. S. Vinding, I. I. Maximov, Z. Tošner, N. C. Nielsen, Fast numerical design of spatial-selective RF pulses in MRI using Krotov and quasi-Newton based op timal control methods. J. Chem. Phys. 137, 054203 (2012). 22894341 10.1063/1.4739755

[185] D. M. Reich, M. Ndong, C. P. Koch, Monotonically convergent optimization in quantum control using Krotov’s method. J. Chem. Phys. 136, 104103 (2012). 22423824 10.1063/1.3691827

[186] M. H. Goerz, D. Basilewitsch, F. Gago-Encinas, M. G. Krauss, K. P. Horn, D. M. Reich, C. P. Koch, Krotov: A Python implementation of Krotov’s method for quantum optimal control. SciPost Phys. 7, 80 (2019).

[187] P. Doria, T. Calarco, S. Montangero, Optimal control technique for many-body quantum dynamics. Phys. Rev. Lett. 106, 190501 (2011). 21668132 10.1103/PhysRevLett.106.190501

[188] S. Machnes, E. Assémat, D. Tannor, F. K. Wilhelm, Tunable, flexible, and efficient optimization of control pulses for practical qubits. Phys. Rev. Lett. 120, 150401 (2018). 29756895 10.1103/PhysRevLett.120.150401

[189] A. Borzì, G. Ciaramella, M. Sprengel, *Formulation and Numerical Solution of Quantum Control Problems*, Computational Science & Engineering (Society for Industrial and Applied Mathematics (SIAM), 2017), vol. 16, pp. x+390.

[190] H. J. Hogben, M. Krzystyniak, G. T. P. Charnock, P. J. Hore, I. Kuprov, *Spinach*—A software library for simulation of spin dynamics in large spin systems. J. Magn. Reson. 208, 179–194 (2011). 21169043 10.1016/j.jmr.2010.11.008

[191] M. Bak, J. T. Rasmussen, N. C. Nielsen, SIMPSON: A general simulation program for solid-state NMR spectroscopy. J. Magn. Reson. 147, 296–330 (2000). 11097821 10.1006/jmre.2000.2179

[192] Z. Tošner, T. Vosegaard, C. Kehlet, N. Khaneja, S. J. Glaser, N. C. Nielsen, Optimal control in NMR spectroscopy: Numerical implementation in SIMPSON. J. Magn. Reson. 197, 120–134 (2009). 19119034 10.1016/j.jmr.2008.11.020

[193] D. L. Goodwin, J. P. Carvalho, A. B. Nielsen, N. Wili, A. Brinkmann, T. Vosegaard, Z. Tošner, N. C. Nielsen, Extending numerical simulations in SIMPSON: Electron paramagnetic resonance, dynamic nuclear polarisation, propagator splitting, pulse transients, and quadrupolar cross terms. J. Magn. Reson. Open 27, 100218 (2026).

[194] J. R. Johansson, P. D. Nation, F. Nori, Qutip: An open-source python framework for the dynamics of open quantum systems. Comput. Phys. Commun. 183, 1760–1772 (2012).

[195] M. Rossignolo, T. Reisser, A. Marshall, P. Rembold, A. Pagano, P. J. Vetter, R. S. Said, M. M. Müller, F. Motzoi, T. Calarco, F. Jelezko, S. Montangero, QuOCS: The quantum optimal control suite. Comput. Phys. Commun. 291, 108782 (2023).

[196] M. S. Silver, R. I. Joseph, D. I. Hoult, Highly selective π/2 and π pulse generation. J. Magn. Reson. 59, 347 (1984).

[197] D. Rosenfeld, Y. Zur, Design of adiabatic selective pulses using optimal control theory. Magn. Reson. Med. 36, 401–409 (1996). 8875410 10.1002/mrm.1910360311

[198] D. Xu, K. F. King, Y. Zhu, G. C. McKinnon, Z.-P. Liang, Designing multichannel, multidimensional, arbitrary flip angle RF pulses using an optimal control approach. Magn. Reson. Med. 59, 547–560 (2008). 18306407 10.1002/mrm.21485 PMC2650716

[199] N. I. Gershenzon, K. Kobzar, B. Luy, S. J. Glaser, T. E. Skinner, Optimal control design of excitation pulses that accommodate relaxation. J. Magn. Reson. 188, 330–336 (2007). 17804269 10.1016/j.jmr.2007.08.007

[200] T. E. Skinner, N. I. Gershenzon, M. Nimbalkar, S. J. Glaser, Optimal control design of band-selective excitation pulses that accommodate relaxation and RF inhomogeneity. J. Magn. Reson. 217, 53–60 (2012). 22425442 10.1016/j.jmr.2012.02.007

[201] M. A. Janich, R. F. Schulte, M. Schwaiger, S. J. Glaser, Robust slice-selective broadband refocusing pulses. J. Magn. Reson. 213, 126–135 (2011). 21974997 10.1016/j.jmr.2011.09.025

[202] M. A. Janich, M. I. Menzel, F. Wiesinger, E. Weidl, O. Khegai, J. H. Ardenkjaer-Larsen, S. J. Glaser, A. Haase, R. F. Schulte, M. Schwaiger, Effects of pyruvate dose on in vivo metabolism and quantification of hyperpolarized ^13^C spectra. NMR Biomed. 25, 142–151 (2012). 21823181 10.1002/nbm.1726

[203] J. Asslaender, S. J. Glaser, J. Hennig, Application of spin echoes in the regime of weak dephasing to T1 mapping of the lung. Magn. Reson. Med. 79, 960–967 (2018). 28419591 10.1002/mrm.26719 PMC5645219

[204] E. Van Reeth, P. M. Lefebvre, H. Ratiney, S. A. Lambert, M. Tesch, E. Brusseau, D. Grenier, O. Beuf, S. J. Glaser, D. Sugny, K. Tse-Ve-Koon, Constant gradient elastography with optimal control RF pulses. J. Magn. Reson. 294, 153–161 (2018). 30053754 10.1016/j.jmr.2018.07.013

[205] C. S. Aigner, C. Clason, A. Rund, R. Stollberger, Efficient high-resolution RF pulse design applied to simultaneous multi-slice excitation. J. Magn. Reson. 263, 33–44 (2016). 26773524 10.1016/j.jmr.2015.11.013

[206] A. Rund, C. S. Aigner, K. Kunisch, R. Stollberger, Magnetic resonance RF pulse design by optimal control with physical constraints. IEEE Trans. Med. Imaging 37, 461–472 (2018). 28981407 10.1109/TMI.2017.2758391

[207] M. S. Vinding, D. Brenner, D. H. Y. Tse, S. Vellmer, T. Vosegaard, D. Suter, T. Stöcker, I. I. Maximov, Application of the limited-memory quasi-Newton algo rithm for multi-dimensional, large flip-angle RF pulses at 7T. Magn. Reson. Mater. Phys. Biol Med. 30, 29–39 (2017). 10.1007/s10334-016-0580-1 27485854

[208] C. S. Aigner, A. Rund, S. Abo Seada, A. N. Price, J. V. Hajnal, S. J. Malik, K. Kunisch, R. Stollberger, Time optimal control-based RF pulse design under gradient imperfections. Magn. Reson. Med. 83, 561–574 (2020). 31441536 10.1002/mrm.27955 PMC6899978

[209] W. A. Grissom, D. Xu, A. B. Kerr, J. A. Fessler, D. C. Noll, Fast large-tip-angle multidimensional and parallel RF pulse design in MRI. IEEE Trans. Med. Imaging 28, 1548–1559 (2009). 19447704 10.1109/TMI.2009.2020064 PMC2763429

[210] M. S. Vinding, C. Laustsen, I. I. Maximov, L. V. Søgaard, J. H. Ardenkjær-Larsen, N. C. Nielsen, Dynamic nuclear polarization and optimal control spatial-selective ^13^C MRI and MRS. J. Magn. Reson. 227, 57–61 (2013). 23298857 10.1016/j.jmr.2012.12.002

[211] I. I. Maximov, M. S. Vinding, D. H. Y. Tse, N. C. Nielsen, N. J. Shah, Real-time 2D spatially selective MRI experiments: Comparative analysis of optimal control design methods. J. Magn. Reson. 254, 110–120 (2015). 25863895 10.1016/j.jmr.2015.03.003

[212] X. Wu, S. Schmitter, E. J. Auerbach, S. Moeller, K. Uğurbil, P.-F. Van de Moortele, Simultaneous multislice multiband parallel radiofrequency excitation with independent slice-specific transmit B1 homogenization. Magn. Reson. Med. 70, 630–638 (2013). 23801410 10.1002/mrm.24828 PMC3884042

[213] J. Yang, J.-F. Nielsen, J. A. Fessler, Y. Jiang, Multidimensional RF pulse design using auto-differentiable spin-domain optimization and its application to reduced field-of-view imaging. Magn. Reson. Med. 94, 1963–1981 (2025). 40523117 10.1002/mrm.30607 PMC12228547

[214] A. Zwick, D. Suter, G. Kurizki, G. A. Álvarez, Precision limits of tissue microstructure characterization by magnetic resonance imaging. Phys. Rev. Appl. 14, 024088 (2020).

[215] B. Bonnard, O. Cots, S. J. Glaser, M. Lapert, D. Sugny, Y. Zhang, Geometric optimal control of the contrast imaging problem in nuclear magnetic resonance. IEEE Trans. Automat. Contr. 57, 1957–1969 (2012).

[216] E. Assémat, M. Lapert, D. Sugny, S. J. Glaser, On the application of geometric optimal control theory to nuclear magnetic resonance. Math. Control Relat. Fields 3, 375–396 (2013).

[217] E. V. Reeth, H. Ratiney, M. Lapert, S. J. Glaser, D. Sugny, Optimal control theory for applications in magnetic resonance imaging. Pac. J. Math. Ind. 9, 9 (2017).

[218] E. Van Reeth, H. Ratiney, M. Tesch, D. Grenier, O. Beuf, S. J. Glaser, D. Sugny, Optimal control design of preparation pulses for contrast optimization in MRI. J. Magn. Reson. 279, 39–50 (2017). 28460243 10.1016/j.jmr.2017.04.012

[219] E. V. Reeth, H. Ratiney, K. T. V. Koon, M. Tesch, D. Grenier, O. Beuf, S. J. Glaser, D. Sugny, A simplified framework to optimize MRI contrast preparation. Magn. Reson. Med. 81, 424–438 (2019). 30265759 10.1002/mrm.27417

[220] M. S. Vinding, B. Guérin, T. Vosegaard, N. C. Nielsen, Local SAR, global SAR, and power-constrained large-flip-angle pulses with optimal control and virtual observation points. Magn. Reson. Med. 77, 374–384 (2017). 26715084 10.1002/mrm.26086 PMC4929033

[221] D. O. Brunner, K. P. Pruessmann, Optimal design of multiple-channel RF pulses under strict power and SAR constraints. Magn. Reson. Med. 63, 1280–1291 (2010). 20432299 10.1002/mrm.22330

[222] C. Graf, M. Soellradl, C. S. Aigner, A. Rund, R. Stollberger, Advanced design of MRI inversion pulses for inhomogeneous field conditions by optimal control. NMR Biomed. 35, e4790 (2022). 35731240 10.1002/nbm.4790 PMC9786750

[223] M. Carravetta, M. H. Levitt, Long-lived nuclear spin states in high-field solution NMR. J. Am. Chem. Soc. 126, 6228–6229 (2004). 15149209 10.1021/ja0490931

[224] W. S. Warren, E. Jenista, R. T. Branca, X. Chen, Increasing hyperpolarized spin lifetimes through true singlet eigenstates. Science 323, 1711–1714 (2009). 19325112 10.1126/science.1167693 PMC3080756

[225] G. Pileio, S. Bowen, C. Laustsen, M. C. D. Tayler, J. T. Hill-Cousins, L. J. Brown, R. C. D. Brown, J. H. Ardenkjaer-Larsen, M. H. Levitt, Recycling and imaging of nuclear singlet hyperpolarization. J. Am. Chem. Soc. 135, 5084–5088 (2013). 23489087 10.1021/ja312333v

[226] J. H. Ardenkjær-Larsen, B. Fridlund, A. Gram, G. Hansson, L. Hansson, M. H. Lerche, R. Servin, M. Thaning, K. Golman, Increase in signal-to-noise ratio of > 10,000 times in liquid-state NMR. Proc. Natl. Acad. Sci. U.S.A. 100, 10158–10163 (2003). 12930897 10.1073/pnas.1733835100 PMC193532

[227] C. Laustsen, S. Bowen, M. S. Vinding, N. C. Nielsen, J. H. Ardenkjaer-Larsen, Storage of magnetization as singlet order by optimal control designed pulses. Magn. Reson. Med. 71, 921–926 (2014). 23554018 10.1002/mrm.24733

[228] J.-X. Xin, G. Yang, H. Zhang, J. Li, C. Fu, J. Wang, R. Tong, Y. Ren, D.-X. Wei, Y.-F. Yao, Using optimal controlled singlet spin order to accurately target molecular signal in MRI and MRS. Sci. Rep. 13, 2212 (2023). 36750607 10.1038/s41598-023-28425-2 PMC9905495

[229] M. Holbach, J. Lambert, D. Suter, Optimized multiple-quantum filter for robust selective excitation of metabolite signals. J. Magn. Reson. 243, 8–16 (2014). 24705532 10.1016/j.jmr.2014.03.007

[230] M. Holbach, J. Lambert, S. Johst, M. E. Ladd, D. Suter, Optimized selective lactate excitation with a refocused multiple-quantum filter. J. Magn. Reson. 255, 34–38 (2015). 25909643 10.1016/j.jmr.2015.03.004

[231] M. S. Vinding, C. S. Aigner, S. Schmitter, T. E. Lund, DeepControl: 2DRF pulses facilitating $B_{1}^{+}$ inhomogeneity and B_0_ off-resonance compensation in vivo at 7 T. Magn. Reson. Med. 85, 3308–3317 (2021). 33480029 10.1002/mrm.28667

[232] M. S. Vinding, D. L. Goodwin, I. Kuprov, T. E. Lund, Optimal control gradient precision trade-offs: Application to fast generation of DeepControl libraries for MRI. J. Magn. Reson. 333, 107094 (2021). 34794089 10.1016/j.jmr.2021.107094

[233] Y. Zhang, K. Jiang, W. Jiang, N. Wang, A. J. Wright, A. Liu, J. Wang, Multi-task convolutional neural network-based design of radio frequency pulse and the accompanying gradients for magnetic resonance imaging. NMR Biomed. 34, e4443 (2021). 33200468 10.1002/nbm.4443

[234] D. Shin, Y. Kim, C. Oh, H. An, J. Park, J. Kim, J. Lee, Deep reinforcement learning-designed radio frequency waveform in MRI. Nat. Mach. Intell. 3, 985–994 (2021).

[235] A. Jang, X. He, F. Liu, Physics-guided self supervised learning: Demonstration for generalized RF pulse design. Magn. Reson. Med. 93, 657–672 (2025). 39385438 10.1002/mrm.30307 PMC11604838

[236] T. E. Skinner, T. O. Reiss, B. Luy, N. Khaneja, S. J. Glaser, Reducing the duration of broadband excitation pulses using optimal control with limited RF amplitude. J. Magn. Reson. 167, 68–74 (2004). 14987600 10.1016/j.jmr.2003.12.001

[237] M. H. Levitt, R. R. Ernst, Improvement of pulse performance in N.M.R. coherence transfer experiments. Mol. Phys. 50, 1109–1124 (1983).

[238] M. Braun, S. J. Glaser, Cooperative pulses. J. Magn. Reson. 207, 114–123 (2010). 20869893 10.1016/j.jmr.2010.08.013

[239] D. Wei, Y. Chang, S. J. Glaser, X. Yang, Cooperative pulses for pseudo-pure state preparation. Appl. Phys. Lett. 104, 242409 (2014).

[240] T. E. Skinner, M. Braun, K. Woelk, N. I. Gershenzon, S. J. Glaser, Design and application of robust RF pulses for toroid cavity NMR spectroscopy. J. Magn. Reson. 209, 282–290 (2011). 21367632 10.1016/j.jmr.2011.01.026

[241] T. E. Skinner, K. Kobzar, B. Luy, M. R. Bendall, W. Bermel, N. Khaneja, S. J. Glaser, Optimal control design of constant amplitude phase-modulated pulses: Application to calibration-free broadband excitation. J. Magn. Reson. 179, 241–249 (2006). 16413802 10.1016/j.jmr.2005.12.010

[242] J.-S. Li, J. Ruths, S. J. Glaser, Exact broadband excitation of two-level systems by mapping spins to springs. Nat. Commun. 8, 446 (2017). 28874703 10.1038/s41467-017-00441-7 PMC5585281

[243] P. Coote, W. Bermel, H. Arthanari, Optimization of phase dispersion enables broadband excitation without homonuclear coupling artifacts. J. Magn. Reson. 327, 106979 (2021). 10.1016/j.jmr.2021.106928 PMC8012116 33652210

[244] P. E. Spindler, Y. Zhang, B. Endeward, N. Gershernzon, T. E. Skinner, S. J. Glaser, T. F. Prisner, Shaped optimal control pulses for increased excitation band width in EPR. J. Magn. Reson. 218, 49–58 (2012). 22578555 10.1016/j.jmr.2012.02.013

[245] Y. T. Woordes, K. Kobzar, S. Ehni, B. Görling, F. Schilling, A. Seliwjorstow, Z. L. Pianowski, P. W. Roesky, S. Bräse, J. Eppinger, S. J. Glaser, B. Luy, Ultrabroadband 1D and 2D NMR spectroscopy. Angew. Chem. Int. Ed. Engl. 65, e15467 (2025). 41307299 10.1002/anie.202515467 PMC12790362

[246] K. Kobzar, T. E. Skinner, N. Khaneja, S. J. Glaser, B. Luy, Exploring the limits of broadband excitation and inversion: II. RF-power optimized pulses. J. Magn. Reson. 194, 58–66 (2008). 18586540 10.1016/j.jmr.2008.05.023

[247] T. E. Skinner, N. I. Gershenzon, M. Nimbalkar, W. Bermel, B. Luy, S. J. Glaser, New strategies for designing robust universal rotation pulses: Application to broadband refocusing at low power. J. Magn. Reson. 216, 78–87 (2012). 22325853 10.1016/j.jmr.2012.01.005

[248] S. L. Altmann, *Rotations, Quaternions, and Double Groups* (Oxford Univ. Press, 1986).

[249] T. Schulte-Herbrüggen, A. Spörl, N. Khaneja, S. J. Glaser, Optimal control-based efficient synthesis of building blocks of quantum algorithms: A perspective from network complexity towards time complexity. Phys. Rev. A 72, 042331 (2005).

[250] M. A. Janich, M. A. McLean, R. Noeske, S. J. Glaser, R. F. Schulte, Slice-selective broadband refocusing pulses for the robust generation of crushed spin-echoes. J. Magn. Reson. 223, 129–137 (2012). 22975241 10.1016/j.jmr.2012.08.003

[251] A. Spoerl, T. Schulte-Herbrüggen, S. J. Glaser, V. Bergholm, M. J. Storcz, J. Ferber, F. K. Wilhelm, Optimal control of coupled Josephson qubits. Phys. Rev. A 75, 012302 (2007).

[252] A. Walther, B. Julsgaard, L. Rippe, Y. Ying, S. Kröll, R. Fisher, S. J. Glaser, Extracting high fidelity quantum computer hardware from random systems. Phys. Scr. T137, 014009 (2009).

[253] P. W. Coote, S. A. Robson, A. Dubey, A. Boeszoermenyi, M. Zhao, G. Wagner, H. Arthanari, Optimal control theory enables homonuclear decoupling without Bloch–Siegert shifts in NMR spectroscopy. Nat. Commun. 9, 3014 (2018). 30069002 10.1038/s41467-018-05400-4 PMC6070575

[254] M. Nimbalkar, B. Luy, T. E. Skinner, J. L. Neves, N. I. Gershenzon, K. Kobzar, W. Bermel, S. J. Glaser, The Fantastic Four: A plug ‘n’ play set of optimal control pulses for enhancing NMR spectroscopy. J. Magn. Reson. 228, 16–31 (2013). 23333616 10.1016/j.jmr.2012.12.007

[255] C. J. Buchanan, G. Bhole, G. Karunanithy, V. Casablancas-Antràs, A. W. J. Poh, B. G. Davis, J. A. Jones, A. J. Baldwin, Seedless: On-the fly pulse calculation for NMR experiments. Nat. Commun. 16, 7276 (2025). 40775231 10.1038/s41467-025-61663-8 PMC12332029

[256] S. Lim, B. Guo, A. Turner, C. Buchanan, A. J. Baldwin, J. A. Jones, Multi-spin control from one spin pulses. arXiv:2602.10861 [quant-ph] (2026). 10.1016/j.jmr.2026.108095 42349035

[257] S. Slad, W. Bermel, R. Kümmerle, D. Mathieu, B. Luy, Band-selective universal 90° and 180° rotation pulses covering the aliphatic carbon chemical shift range for triple resonance experiments on 1.2 GHz spectrometers. J. Biomol. NMR 76, 185–195 (2022). 36418752 10.1007/s10858-022-00404-1 PMC9712393

[258] D. Joseph, C. Griesinger, Optimal control pulses for the 1.2-GHz (28.2-T) NMR spectrometers. Sci. Adv. 9, eadj1133 (2023). 37948513 10.1126/sciadv.adj1133 PMC10637738

[259] E. Assémat, M. Lapert, Y. Zhang, M. Braun, S. J. Glaser, D. Sugny, Simultaneous time-optimal control of the inversion of two spin-1/2 particles. Phys. Rev. A 82, 013415 (2010).

[260] E. Assemat, L. Attar, M.-J. Penouilh, M. Picquet, A. Tabard, Y. Zhang, S. J. Glaser, D. Sugny, Optimal control of the inversion of two spins in nuclear magnetic resonance. Chem. Phys. 405, 71–75 (2012).

[261] Q. Ansel, S. J. Glaser, D. Sugny, Selective and robust time-optimal rotations of spin systems. J. Phys. A Math. Theor. 54, 085204 (2021).

[262] L. Van Damme, D. Sugny, S. J. Glaser, Application of the small-tip-angle approximation in the toggling frame for the design of analytic robust pulses in quantum control. Phys. Rev. A 104, 042226 (2021).

[263] M. Lapert, Y. Zhang, M. Braun, S. J. Glaser, D. Sugny, Geometric versus numerical optimal control of a dissipative spin-1/2 particle. Phys. Rev. A 82, 063418 (2010). 10.1103/PhysRevLett.104.083001 20366927

[264] F. Mintert, M. Lapert, Y. Zhang, S. J. Glaser, D. Sugny, Saturation of a spin-1/2 particle by generalized local control. New J. Phys. 13, 073001 (2011).

[265] B. Bonnard, S. J. Glaser, D. Sugny, A review of geometric optimal control for quantum systems in nuclear magnetic resonance. Adv. Math. Phys. 2012, 1–29 (2012).

[266] T. Untidt, T. Schulte-Herbrüggen, B. Luy, S. J. Glaser, C. Griesinger, O. W. Sørensen, N. C. Nielsen, Design of NMR pulse experiments with optimum sensitivity: Coherence-order-selective transfer in I_2_S and I_3_S spin systems. Mol. Phys. 95, 787–796 (1998).

[267] N. Khaneja, F. Kramer, S. J. Glaser, Optimal experiments for maximizing coherence transfer between coupled spins. J. Magn. Reson. 173, 116–124 (2005). 15705519 10.1016/j.jmr.2004.11.023

[268] N. Khaneja, S. J. Glaser, Time-optimal coherence-order-selective transfer of in-phase coherence in heteronuclear IS spin systems. J. Magn. Reson. 154, 192–195 (2002). 11846576 10.1006/jmre.2001.2480

[269] J. A. M. Rodríguez, A. C. R. Dutra, H. N. S. Earp, M. T. Cunha, Cartan-Khaneja-Glaser decompo sition of *SU*(2*^n^*) via involutive automorphisms. arXiv:2509.05468 [quant-ph] (2026).

[270] N. Khaneja, S. J. Glaser, R. Brockett, Erratum: Sub-Riemannian geometry and time optimal control of three spin systems: Quantum gates and coherence transfer [Phys. Rev. A 65, 032301 (2002)]. Phys. Rev. A 71, 039906 (2005).

[271] R. W. Brockett, “Control theory and analytical mechanics,” in *New Directions in Applied Mathematics*, P. J. Hilton, G. S. Young, Eds. (Springer, 1982), pp. 11–46.

[272] T. O. Reiss, N. Khaneja, S. J. Glaser, Broadband geodesic pulses for three-spin systems: Time-optimal realization of effective trilinear coupling terms and in direct SWAP gates. J. Magn. Reson. 165, 95–101 (2003). 14568520 10.1016/s1090-7807(03)00245-3

[273] C.-H. Tseng, S. Somaroo, Y. Sharf, E. Knill, R. Laflamme, T. F. Havel, D. G. Cory, Quantum simulation of a three-body-interaction Hamiltonian on an NMR quantum computer. Phys. Rev. A 61, 012302 (1999).

[274] H. Yuan, D. Wei, Y. Zhang, S. J. Glaser, N. Khaneja, Efficient synthesis of quantum gates on indirectly coupled spins. Phys. Rev. A 89, 042315 (2014).

[275] L. Van Damme, R. Zeier, S. J. Glaser, D. Sugny, Application of the Pontryagin maximum principle to the time-optimal control in a chain of three spins with unequal couplings. Phys. Rev. A 90, 013409 (2014).

[276] S. S. Köcher, T. Heydenreich, Y. Zhang, G. N. M. Reddy, S. Caldarelli, H. Yuan, S. J. Glaser, Time optimal excitation of maximum quantum coherence: Physical limits and pulse sequences. J. Chem. Phys. 144, 164103 (2016). 27131527 10.1063/1.4945781

[277] J. L. Neves, B. Heitmann, T. O. Reiss, H. H. R. Schor, N. Khaneja, S. J. Glaser, Exploring the limits of polarization transfer efficiency in homonuclear three-spin systems. J. Magn. Reson. 181, 126–134 (2006). 16644249 10.1016/j.jmr.2006.03.021

[278] M. Nimbalkar, R. Zeier, J. L. Neves, S. B. Elavarasi, H. Yuan, N. Khaneja, K. Dorai, S. J. Glaser, Multiple-spin coherence transfer in linear Ising spin chains and beyond: Numerically optimized pulses and experiments. Phys. Rev. A 85, 012325 (2012).

[279] S. Ehni, B. Luy, BEBEtr and BUBI: J-compensated concurrent shaped pulses for ^1^H–^13^C experiments. J. Magn. Reson. 232, 7–17 (2013). 23673080 10.1016/j.jmr.2013.04.007

[280] Y. T. Woordes, T. Reinsperger, S. Ehni, B. Luy, Robust bilinear rotations. Sci. Adv. 11, eadx7094 (2025). 40880465 10.1126/sciadv.adx7094 PMC12396312

[281] S. J. Glaser, Coupling topology dependence of polarization-transfer efficiency in TOCSY and TACSY experiments. J. Magn. Reson. Ser. A 104, 283–301 (1993).

[282] D. P. Frueh, T. Ito, J.-S. Li, G. Wagner, S. J. Glaser, N. Khaneja, Sensitivity enhancement in NMR of macro molecules by application of optimal control theory. J. Biomol. NMR 32, 23–30 (2005). 16041480 10.1007/s10858-005-3592-0

[283] G. A. Morris, R. Freeman, Enhancement of nuclear magnetic resonance signals by polarization transfer. J. Am. Chem. Soc. 101, 760–762 (1979).

[284] R. Bellman, *Dynamic Programming* (Princeton Univ. Press, 1957).

[285] R. Brüschweiler, R. R. Ernst, Molecular dynamics monitored by cross-correlated cross relaxation of spins quantized along orthogonal axes. J. Chem. Phys. 96, 1758–1766 (1992).

[286] R. Riek, G. Wider, K. Pervushin, K. Wüthrich, Polarization transfer by cross-correlated relaxation in solution NMR with very large molecules. Proc. Natl. Acad. Sci. U.S.A. 96, 4918–4923 (1999). 10220394 10.1073/pnas.96.9.4918 PMC21792

[287] D. Siminovitch, T. Untidt, N. C. Nielsen, Exact effective Hamiltonian theory. II. Polynomial expansion of matrix functions and entangled unitary exponential operators. J. Chem. Phys. 120, 51–66 (2004). 15267261 10.1063/1.1628216

[288] R. Shankar, M. Ernst, P. K. Madhu, T. Vosegaard, N. C. Nielsen, A. B. Nielsen, A general theoretical description of the influence of isotropic chemical shift in dipolar recoupling experiments for solid-state NMR. J. Chem. Phys. 146, 134105 (2017). 28390347 10.1063/1.4979123

[289] A. B. Nielsen, N. C. Nielsen, Accurate analysis and perspectives for systematic design of magnetic resonance experiments using single-spin vector and exact effective Hamiltonian theory. J. Magn. Reson. Open 12–13, 100064 (2022).

[290] N . C. Nielsen, L. A. Strassø, A. B. Nielsen, Dipolar recoupling, in Solid State NMR, J. C. C. Chan, Ed. (Springer Berlin Heidelberg, 2011), pp. 1–45.

[291] V. Ladizhansky, R. S. Palani, M. Mardini, R. G. Griffin, Dipolar recoupling in rotating solids. Chem. Rev. 124, 12844–12917 (2024). 39504237 10.1021/acs.chemrev.4c00373 PMC12117474

[292] C. T. Kehlet, A. C. Sivertsen, M. Bjerring, T. O. Reiss, N. Khaneja, S. J. Glaser, N. C. Nielsen, Improving solid-state NMR dipolar recoupling by optimal control. J. Am. Chem. Soc. 126, 10202–10203 (2004). 15315406 10.1021/ja048786e

[293] C. Kehlet, M. Bjerring, A. C. Sivertsen, T. Kristensen, J. J. Enghild, S. J. Glaser, N. Khaneja, N. C. Nielsen, Optimal control based NCO and NCA experiments for spectral assignment in biological solid state NMR spectroscopy. J. Magn. Reson. 188, 216–230 (2007). 17681479 10.1016/j.jmr.2007.06.011

[294] N. M. Loening, M. Bjerring, N. C. Nielsen, H. Oschkinat, A comparison of NCO and NCA transfer methods for biological solid-state NMR spectroscopy. J. Magn. Reson. 214, 81–90 (2012). 22116035 10.1016/j.jmr.2011.10.012 PMC3257381

[295] C. Kehlet, T. Vosegaard, N. Khaneja, S. J. Glaser, N. C. Nielsen, Low-power homonuclear dipolar recoupling in solid-state NMR developed using optimal control theory. Chem. Phys. Lett. 414, 204–209 (2005).

[296] J. Ø. Hansen, C. Kehlet, M. Bjerring, T. Vosegaard, S. J. Glaser, N. Khaneja, N. C. Nielsen, Optimal control based design of composite dipolar recoupling experiments by analogy to single-spin inversion pulses. Chem. Phys. Lett. 447, 154–161 (2007).

[297] Z. Tošner, M. J. Brandl, J. Blahut, S. J. Glaser, B. Reif, Maximizing efficiency of dipolar recoupling in solid-state NMR using optimal control sequences. Sci. Adv. 7, eabj5913 (2021). 34644121 10.1126/sciadv.abj5913 PMC8514097

[298] Z. Tošner, R. Sarkar, J. Becker-Baldus, C. Glaubitz, S. Wegner, F. Engelke, S. J. Glaser, B. Reif, Overcoming volume selectivity of dipolar recoupling in biological solid-state NMR spectroscopy. Angew. Chem. Int. Ed. Engl. 57, 14514–14518 (2018). 29989288 10.1002/anie.201805002

[299] T. Vosegaard, C. Kehlet, N. Khaneja, S. J. Glaser, N. C. Nielsen, Improved excitation schemes for multiple quantum magic-angle spinning for quadrupolar nuclei designed using optimal control theory. J. Am. Chem. Soc. 127, 13768–13769 (2005). 16201779 10.1021/ja054035g

[300] D. Wei, Ü. Akbey, B. Paaske, H. Oschkinat, B. Reif, M. Bjerring, N. C. Nielsen, Optimal ^2^H RF pulses and ^2^H–^13^C cross-polarization methods for solid-state ^2^H MAS NMR of perdeuterated proteins. J. Phys. Chem. Lett. 2, 1289–1294 (2011). 26295423 10.1021/jz200511b

[301] S. Jain, M. Bjerring, N. C. Nielsen, Efficient and robust heteronuclear cross-polarization for high-speed spinning biological solid-state NMR spectroscopy. J. Phys. Chem. Lett. 3, 703–708 (2012). 26286276 10.1021/jz3000905

[302] A. B. Nielsen, S. Jain, M. Ernst, B. H. Meier, N. C. Nielsen, Adiabatic rotor-echo-short-pulse irradiation mediated cross-polarization. J. Magn. Reson. 237, 147–151 (2013). 24220613 10.1016/j.jmr.2013.09.002

[303] S. K. Jain, A. B. Nielsen, M. Hiller, L. Handel, M. Ernst, H. Oschkinat, Ü. Akbey, N. C. Nielsen, Low-power polarization transfer between deuterons and spin-1/2 nuclei using adiabatic (RESPIRATION)CP in solid-state NMR. Phys. Chem. Chem. Phys. 16, 2827–2830 (2014). 24418905 10.1039/c3cp54419b

[304] S. Ray, V. S. Redrouthu, A. Equbal, S. K. Jain, Optimal control-based nuclear spin cross-polarization in the presence of complicating anisotropic interactions. Phys. Chem. Chem. Phys. 27, 7016–7027 (2025). 40047693 10.1039/d5cp00096c

[305] C. Kehlet, J. T. Nielsen, Z. Tosner, N. C. Nielsen, Resolution-enhanced solid-state NMR ^13^C-^13^C correlation spectroscopy by optimal control dipolar-driven spin-state-selective coherence transfer. J. Phys. Chem. Lett. 2, 543–547 (2011).

[306] N. B. Manson, J. P. Harrison, M. J. Sellars, Nitrogen-vacancy center in diamond: Model of the electronic structure and associated dynamics. Phys. Rev. B 74, 104303 (2006).

[307] J. Cavanagh, M. Rance, Sensitivity improvement in isotropic mixing (TOCSY) experiments. J. Magn. Reson. 88, 72–85 (1990).

[308] Z. Tošner, S. J. Glaser, N. Khaneja, N. C. Nielsen, Effective Hamiltonians by optimal control: Solid-state NMR double-quantum planar and isotropic dipolar recoupling. J. Chem. Phys. 125, 184502 (2006). 17115760 10.1063/1.2366703

[309] J. Blahut, M. J. Brandl, T. Pradhan, B. Reif, Z. Tošner, Sensitivity-enhanced multidimensional solid-state NMR spectroscopy by optimal-control-based transverse mixing sequences. J. Am. Chem. Soc. 144, 17336–17340 (2022). 36074981 10.1021/jacs.2c06568

[310] T. Kaufmann, T. J. Keller, J. M. Franck, R. P. Barnes, S. J. Glaser, J. M. Martinis, S. Han, DAC-board based X-band EPR spectrometer with arbitrary wave form control. J. Magn. Reson. 235, 95–108 (2013). 23999530 10.1016/j.jmr.2013.07.015 PMC3863685

[311] U. Rasulov, I. Kuprov, Instrumental distortions in quantum optimal control. J. Chem. Phys. 162, 164107 (2025). 40277087 10.1063/5.0264092

[312] J. Stropp, N. Wili, N. C. Nielsen, D. Klose, Increased sensitivity in electron–nuclear double resonance spectroscopy with chirped radiofrequency pulses. Magn. Reson. 6, 33–42 (2025). 10.5194/mr-6-33-2025 PMC12247087 40657611

[313] C. Bretschneider, A. Karabanov, N. C. Nielsen, W. Köckenberger, Conversion of parahydrogen induced longitudinal two-spin order to evenly distributed single spin polarisation by optimal control pulse sequences. J. Chem. Phys. 136, 094201 (2012). 22401433 10.1063/1.3691193

[314] A. Henstra, P. Dirksen, J. Schmidt, W. T. Wenckebach, Nuclear spin orientation via electron spin locking (NOVEL). J. Magn. Reson. 77, 389–393 (1988).

[315] A. B. Nielsen, J. P. A. Carvalho, D. L. Goodwin, N. Wili, N. C. Nielsen, Dynamic nuclear polarization pulse sequence engineering using single-spin vector effective Hamiltonians. Phys. Chem. Chem. Phys. 26, 28208–28219 (2024). 39498786 10.1039/d4cp03041a

[316] N. Wili, A. B. Nielsen, J. P. Carvalho, N. C. Nielsen, Observation of dynamic nuclear polarization echoes. Sci Adv. 10, eadr2420 (2024). 39423270 10.1126/sciadv.adr2420 PMC11488531

[317] A. Marshall, T. Reisser, P. Rembold, C. Müller, J. Scheuer, M. Gierse, T. Eichhorn, J. M. Steiner, P. Hautle, T. Calarco, F. Jelezko, M. B. Plenio, S. Montangero, I. Schwartz, M. M. Müller, P. Neumann, Macroscopic hyperpolarization enhanced with quantum optimal control. Phys. Rev. Res. 4, 043179 (2022).

[318] F. V. Jensen, J. P. Carvalho, N. Wili, A. H. Thomsen, D. L. Goodwin, L. Trottner, C. Strauch, A. B. Nielsen, N. C. Nielsen, In situ learning-based spin engineering of pulsed dynamic nuclear polarization. Sci. Adv. 12, eaeh4467 (2026).

[319] J. Carvalho, A. B. Nielsen, E. Baligács, N. Wili, N. C. Nielsen, Bridging dynamic nuclear polarization and solid-state NMR dipolar recoupling: From static single crystal to spinning powders. J. Phys. Chem. Lett. 16, 4363–4371 (2025). 40272255 10.1021/acs.jpclett.5c00617

[320] E. Baligács, J. P. Carvalho, N. C. Nielsen, A. B. Nielsen, Accordion-like tuning of composite pulse dipolar recoupling in solid-state NMR. J. Chem. Phys. 164, 124201 (2026). 41879187 10.1063/5.0318973

[321] C. Griesinger, M. Bennati, H. M. Vieth, C. Luchinat, G. Parigi, P. Höefer, F. Engelke, S. J. Glaser, V. Denysenkov, T. F. Prisner, Dynamic nuclear polarization at high magnetic fields in liquids. Prog. Nucl. Magn. Reson. Spectrosc. 64, 4–28 (2012). 22578315 10.1016/j.pnmrs.2011.10.002

[322] N. Pomplun, B. Heitmann, N. Khaneja, S. J. Glaser, Optimization of electron-nuclear polarization transfer. Appl. Magn. Reson. 34, 331–346 (2008).

[323] H. Yuan, R. Zeier, N. Pomplun, S. J. Glaser, N. Khaneja, Time-optimal polarization transfer from an electron spin to a nuclear spin. Phys. Rev. A 92, 053414 (2015).

[324] N. Pomplun, S. J. Glaser, Exploring the limits of electron-nuclear polarization transfer efficiency in three-spin systems. Phys. Chem. Chem. Phys. 12, 5791–5798 (2010). 20449517 10.1039/c003751f

[325] E. Knill, R. Laflamme, W. H. Zurek, Resilient quantum computation. Science 279, 342–345 (1998).

[326] J. Preskill, Reliable quantum computers. Proc. R. Soc. Lond. A 454, 385–410 (1998).

[327] D. Suter, G. A. Álvarez, Colloquium: Protecting quantum information against environmental noise. Rev. Mod. Phys. 88, 041001 (2016).

[328] M. W. Doherty, N. B. Manson, P. Delaney, L. C. L. Hollenberg, The negatively charged nitrogen-vacancy centre in diamond: The electronic solution. New J. Phys. 13, 025019 (2011).

[329] D. Suter, F. Jelezko, Single-spin magnetic resonance in the nitrogen-vacancy center of diamond. Prog. Nucl. Magn. Reson. Spectrosc. 98-99, 50–62 (2017). 28283086 10.1016/j.pnmrs.2016.12.001

[330] P. Rembold, N. Oshnik, M. M. Muller, S. Montangero, T. Calarco, E. Neu, Introduction to quantum optimal control for quantum sensing with nitrogen-vacancy centers in diamond. AVS Quantum Sci. 2, 024701 (2020).

[331] J. Zhang, D. Suter, Single NV centers as sensors for radio-frequency fields. Phys. Rev. Res. 5, L022026 (2023).

[332] N. Khaneja, Switched control of electron-nuclear spin systems. Phys. Rev. A 76, 032326 (2007).

[333] S. S. Hegde, J. Zhang, D. Suter, Efficient quantum gates for individual nuclear spin qubits by indirect control. Phys. Rev. Lett. 124, 220501 (2020). 32567913 10.1103/PhysRevLett.124.220501

[334] J. Zhang, S. S. Hegde, D. Suter, Efficient implementation of a quantum algorithm in a single nitrogen vacancy center of diamond. Phys. Rev. Lett. 125, 030501 (2020). 32745418 10.1103/PhysRevLett.125.030501

[335] J. A. Jones, Controlling NMR spin systems for quantum computation. Prog. Nucl. Magn. Reson. Spectrosc. 140-141, 49–85 (2024). 38705636 10.1016/j.pnmrs.2024.02.002

[336] I. L. Chuang, N. Gershenfeld, M. Kubinec, Experimental implementation of fast quantum searching. Phys. Rev. Lett. 80, 3408–3411 (1998).

[337] N. Linden, H. Barjat, R. Freeman, An implementation of the Deutsch–Jozsa algorithm on a three-qubit NMR quantum computer. Chem. Phys. Lett. 296, 61–67 (1998).

[338] R. Marx, A. F. Fahmy, J. M. Myers, W. Bermel, S. J. Glaser, Approaching five-bit NMR quantum computing. Phys. Rev. A 62, 012310 (2000).

[339] S. Fleischer, Y. Khodorkovsky, Y. Prior, I. Sh Averbukh, Controlling the sense of molecular rotation. New J. Phys. 11, 105039 (2009).

[340] N. Timoney, V. Elman, S. J. Glaser, C. Weiss, M. Johanning, W. Neuhauser, C. Wunderlich, Error resistant single-qubit gates with trapped ions. Phys. Rev. A 77, 052334 (2008).

[341] B. Rowland, J. A. Jones, Implementing quantum logic gates with gradient ascent pulse engineering. Philos. Trans. R. Soc. A 370, 4636–4650 (2012). 10.1098/rsta.2011.0361 22946033

[342] Y. Atia, Y. Elias, T. Mor, Y. Weinstein, Quantum computing gates via optimal control. Int. J. Quantum Inf. 12, 1450031 (2014).

[343] Z. Zhang, L. Van Damme, M. Rossignolo, L. Festa, M. Melchner, R. Eberhard, D. Tsevas, K. Mours, E. Reches, J. Zieher, S. Blatt, I. Bloch, S. J. Glaser, A. Alberti, Recoil-free quantum gates with optical qubits. arXiv:2408.04622 [quant-ph] (2024).

[344] T. Schulte-Herbrüggen, A. Spörl, N. Khaneja, S. J. Glaser, Optimal control for generating quantum gates in open dissipative systems. J. Phys. B At. Mol. Opt. Phys. 44, 154013 (2011).

[345] E. M. Wright, L. Van Damme, N. J. Glaser, A. Devra, F. A. Roy, J. Englhardt, N. Bruckmoser, L. Koch, A. Marx, J. Schirk, C. M. F. Schneider, L. Södergren, F. Wallner, S. J. Glaser, M. Werninghaus, S. Filipp, Superconducting-qubit gates robust to parameter fluctuations. Phys. Rev. Appl. 25, 054037 (2026).

[346] R. Fisher, F. Helmer, S. J. Glaser, F. Marquardt, T. Schulte-Herbrüggen, Optimal control of circuit quantum electrodynamics in one and two dimensions. Phys. Rev. B 81, 085328 (2010).

[347] Q. Ansel, S. Probst, P. Bertet, S. J. Glaser, D. Sugny, Optimal control of an inhomogeneous spin ensemble coupled to a cavity. Phys. Rev. A 98, 023425 (2018).

[348] R. Porotti, V. Peano, F. Marquardt, Gradient ascent pulse engineering with feedback. PRX Quantum 4, 030305 (2023).

[349] T. Schulte-Herbrüggen, A. Spörl, N. Khaneja, S. J. Glaser, “Quantum computing implemented via optimal control: Applications to spin and pseudospin systems,” in *Quantum Information*, Wiley-VCH Handbook of Quantum Information, D. Bruss, G. Leuchs, Eds. (Wiley-VCH, 2016), pp. 643–668.

[350] E. Knill, R. Laflamme, R. Martinez, C.-H. Tseng, An algorithmic benchmark for quantum information processing. Nature 404, 368–370 (2000). 10746718 10.1038/35006012

[351] T. Schulte-Herbrüggen, A. Spörl, N. Khaneja, S. J. Glaser, Quantum CISC compilation by optimal control and scalable assembly of complex instruction sets beyond two-qubit gates. arXiv:0712.3227 [quant-ph] (2008).

[352] D. C. McKay, C. J. Wood, S. Sheldon, J. M. Chow, J. M. Gambetta, Efficient Z-gates for quantum computing. arXiv 1612.00858 [quant-ph] (2016).

[353] A. Larrouy, S. Patsch, R. Richaud, J.-M. Raimond, M. Brune, C. P. Koch, S. Gleyzes, Fast navigation in a large Hilbert space using quantum optimal control. Phys. Rev. X 10, 021058 (2020).

[354] S. Patsch, D. M. Reich, J.-M. Raimond, M. Brune, S. Gleyzes, C. P. Koch, Fast and accurate circularization of a Rydberg atom. Phys. Rev. A 97, 053418 (2018).

[355] N. Rach, M. M. Müller, T. Calarco, S. Montangero, Dressing the chopped-random-basis optimization: A bandwidth-limited access to the trap-free landscape. Phys. Rev. A 92, 062343 (2015).

[356] J. Cui, R. van Bijnen, T. Pohl, S. Montangero, T. Calarco, Optimal control of Rydberg lattice gases. Quantum Sci. Technol. 2, 035006 (2017).

[357] A. Omran, H. Levine, A. Keesling, G. Semeghini, T. T. Wang, S. Ebadi, H. Bernien, A. S. Zibrov, H. Pichler, S. Choi, J. Cui, M. Rossignolo, P. Rembold, S. Montangero, T. Calarco, M. Endres, M. Greiner, V. Vuletić, M. D. Lukin, Generation and manipulation of Schrödinger cat states in Rydberg atom arrays. Science 365, 570–574 (2019). 31395778 10.1126/science.aax9743

[358] M. H. Goerz, T. Calarco, C. P. Koch, The quantum speed limit of optimal controlled phasegates for trapped neutral atoms. J. Phys. B At. Mol. Opt. Phys. 44, 154011 (2011).

[359] M. M. Müller, D. M. Reich, M. Murphy, H. Yuan, J. Vala, K. B. Whaley, T. Calarco, C. P. Koch, Optimizing entangling quantum gates for physical systems. Phys. Rev. A 84, 042315 (2011).

[360] M. H. Goerz, E. J. Halperin, J. M. Aytac, C. P. Koch, K. B. Whaley, Robustness of high-fidelity Rydberg gates with single-site addressability. Phys. Rev. A 90, 032329 (2014).

[361] M. M. Muller, M. Murphy, S. Montangero, T. Calarco, P. Grangier, A. Browaeys, Implementation of an experimentally feasible controlled-phase gate on two blockaded Rydberg atoms. Phys. Rev. A 89, 032334 (2014).

[362] T. Pichler, T. Caneva, S. Montangero, M. D. Lukin, T. Calarco, Noise-resistant optimal spin squeezing via quantum control. Phys. Rev. A 93, 013851 (2016).

[363] A. V. Gorshkov, T. Calarco, M. D. Lukin, A. S. Sørensen, Photon storage in Λ-type optically dense atomic media. IV. Optimal control using gradient ascent. Phys. Rev. A 77, 043806 (2008).

[364] L. Giannelli, T. Schmit, T. Calarco, C. P. Koch, S. Ritter, G. Morigi, Optimal storage of a single photon by a single intra-cavity atom. New J. Phys. 20, 105009 (2018).

[365] K. Rojan, D. M. Reich, I. Dotsenko, J.-M. Raimond, C. P. Koch, G. Morigi, Arbitrary-quantum-state preparation of a harmonic oscillator via optimal control. Phys. Rev. A 90, 023824 (2014).

[366] J. Singh, R. Zeier, T. Calarco, F. Motzoi, Compensating for nonlinear distortions in controlled quantum systems. Phys. Rev. Appl. 19, 064067 (2023).

[367] J. Saywell, M. Carey, M. Belal, I. Kuprov, T. Free-garde, Optimal control of Raman pulse sequences for atom interferometry. J. Phys. B At. Mol. Opt. Phys. 53, 085006 (2020).

[368] J. C. Saywell, M. S. Carey, P. S. Light, S. S. Szigeti, A. R. Milne, K. S. Gill, M. L. Goh, V. S. Perunicic, N. M. Wilson, C. D. Macrae, A. Rischka, P. J. Everitt, N. P. Robins, R. P. Anderson, M. R. Hush, M. J. Biercuk, Enhancing the sensitivity of atom interferometric inertial sensors using robust control. Nat. Commun. 14, 7626 (2023). 37993456 10.1038/s41467-023-43374-0 PMC10665367

[369] M. H. Goerz, M. A. Kasevich, V. S. Malinovsky, Robust optimized pulse schemes for atomic fountain interferometry. Atoms 11, 36 (2023).

[370] G. Louie, Z. Chen, T. Deshpande, T. Kovachy, Robust atom optics for Bragg atom interferometry. New J. Phys. 25, 083017 (2023).

[371] A. Micheli, D. Jaksch, J. I. Cirac, P. Zoller, Many particle entanglement in two-component Bose-Einstein condensates. Phys. Rev. A 67, 013607 (2003).

[372] M. Lapert, G. Ferrini, D. Sugny, Optimal control of quantum superpositions in a bosonic Josephson junction. Phys. Rev. A 85, 023611 (2012).

[373] G. Sorelli, M. Gessner, A. Smerzi, L. Pezzè, Fast and optimal generation of entanglement in bosonic Josephson junctions. Phys. Rev. A 99, 022329 (2019).

[374] R. G. Lena, G. M. Palma, G. De Chiara, Work fluctuations in bosonic Josephson junctions. Phys. Rev. A 93, 053618 (2016).

[375] S. C. Carrasco, M. H. Goerz, Z. Li, S. Colombo, V. Vuletić, V. S. Malinovsky, Extreme spin squeezing via optimized one-axis twisting and rotations. Phys. Rev. Appl. 17, 064050 (2022).

[376] J. Denis, C. Read, J. Martin, Coherent generation and protection of anticoherent spin states. SciPost Phys. Core 9, 001 (2026).

[377] R. N. Zare, *Angular Momentum* (Wiley, 1988).

[378] H. Stapelfeldt, T. Seideman, Colloquium: Aligning molecules with strong laser pulses. Rev. Mod. Phys. 75, 543–557 (2003).

[379] C. P. Koch, M. Lemeshko, D. Sugny, Quantum control of molecular rotation. Rev. Mod. Phys. 91, 035005 (2019).

[380] M. Lapert, S. Guérin, D. Sugny, Field-free quantum cogwheel by shaping of rotational wave packets. Phys. Rev. A 83, 013403 (2011).

[381] M. Z. Hoque, M. Lapert, E. Hertz, F. Billard, D. Sugny, B. Lavorel, O. Faucher, Observation of laser-induced field-free permanent planar alignment of molecules. Phys. Rev. A 84, 013409 (2011).

[382] K. Kitano, H. Hasegawa, Y. Ohshima, Ultrafast angular momentum orientation by linearly polarized laser fields. Phys. Rev. Lett. 103, 223002 (2009). 20366091 10.1103/PhysRevLett.103.223002

[383] G. Karras, M. Ndong, E. Hertz, D. Sugny, F. Billard, B. Lavorel, O. Faucher, Polarization shaping for unidirectional rotational motion of molecules. Phys. Rev. Lett. 114, 103001 (2015). 25815926 10.1103/PhysRevLett.114.103001

[384] K. Lin, Q. Song, X. Gong, Q. Ji, H. Pan, J. Ding, H. Zeng, J. Wu, Visualizing molecular unidirectional rotation. Phys. Rev. A 92, 013410 (2015).

[385] J. T. Stahovich, B. A. Rowe, J. Huennekens, A. M. Lyyra, E. H. Ahmed, Molecular angular momentum orientation using dressed states created by laser radiation. Phys. Rev. Lett. 133, 263601 (2024). 39879039 10.1103/PhysRevLett.133.263601

[386] M. Leibscher, E. Pozzoli, A. Blech, M. Sigalotti, U. Boscain, C. P. Koch, Quantum control of rovibrational dynamics and application to light-induced molecular chirality. Phys. Rev. A 109, 012810 (2024).

[387] M. Leibscher, C. P. Koch, Rotational cooling of large trapped molecular ions. arXiv:2506.20846 [quant-ph] (2025).

[388] V. V. Albert, J. P. Covey, J. Preskill, Robust encoding of a qubit in a molecule. Phys. Rev. X 10, 031050 (2020).

[389] N. R. Hutzler, Polyatomic molecules as quantum sensors for fundamental physics. Quantum Sci. Technol. 5, 044011 (2020).

[390] S. P. Jordan, Fast quantum algorithm for numerical gradient estimation. Phys. Rev. Lett. 95, 050501 (2005). 16090859 10.1103/PhysRevLett.95.050501

[391] T. S. Mahesh, D. Suter, Quantum-information processing using strongly dipolar coupled nuclear spins. Phys. Rev. A 74, 062312 (2006).

[392] J. Li, X. Yang, X. Peng, C.-P. Sun, Hybrid quantum-classical approach to quantum optimal control. Phys. Rev. Lett. 118, 150503 (2017). 28452527 10.1103/PhysRevLett.118.150503

[393] X. Yang, J. Thompson, Z. Wu, M. Gu, X. Peng, J. Du, Probe optimization for quantum metrology via closed-loop learning control. npj Quantum Inf. 6, 62 (2020).

[394] X. Yang, X. Chen, J. Li, X. Peng, R. Laflamme, Hybrid quantum-classical approach to enhanced quantum metrology. Sci. Rep. 11, 672 (2021). 33436795 10.1038/s41598-020-80070-1 PMC7803758

[395] R. Liu, Z. Wu, X. Yang, Y. Li, H. Zhou, Z. Li, Y. Chen, H. Yuan, X. Peng, Variational quantum metrology with the Loschmidt echo. Natl. Sci. Rev. 12, nwaf091 (2025). 40290589 10.1093/nsr/nwaf091 PMC12023863

[396] A. G. R. Day, M. Bukov, P. Weinberg, P. Mehta, D. Sels, Glassy phase of optimal quantum control. Phys. Rev. Lett. 122, 020601 (2019). 30720331 10.1103/PhysRevLett.122.020601

[397] M. Bukov, A. G. R. Day, D. Sels, P. Weinberg, A. Polkovnikov, P. Mehta, Reinforcement learning in different phases of quantum control. Phys. Rev. X 8, 031086 (2018).

[398] C. W. Duncan, P. M. Poggi, M. Bukov, N. T. Zinner, S. Campbell, Taming quantum systems: A tutorial for using shortcuts-to-adiabaticity, quantum optimal control, and reinforcement learning. PRX Quantum 6, 040201 (2025).

[399] H. Ma, B. Qi, I. R. Petersen, R.-B. Wu, H. Rabitz, D. Dong, Machine learning for estimation and control of quantum systems. Natl. Sci. Rev. 12, nwaf269 (2025). 40860018 10.1093/nsr/nwaf269 PMC12371192

[400] T. Kiermeyer, T. Heydenreich, L. Van Damme, S. Hohenemser, F. Marquardt, S. J. Glaser, Uncovering latent structures in robust pulse sequences: A model-based reinforcement learning approach for adaptable quantum control. arXiv:2606.24507 [quant-ph] (2026).

[401] L. Van Damme, D. Leiner, P. Mardešić, S. J. Glaser, D. Sugny, Linking the rotation of a rigid body to the Schrödinger equation: The quantum tennis racket effect and beyond. Sci. Rep. 7, 3998 (2017). 28638097 10.1038/s41598-017-04174-x PMC5479806

[402] S. Asami, W. Kallies, J. C. Guenther, M. Stavropoulou, S. J. Glaser, M. Sattler, Back cover: Ultrashort broadband cooperative pulses for multidimensional biomolecular NMR experiments. Angew. Chem. Int. Ed. Engl. 57, 14498–14502 (2018). 29508496 10.1002/anie.201800220

